# Synthesis of imidazo[1,2-*a*]pyridine-containing peptidomimetics by tandem of Groebke–Blackburn–Bienaymé and Ugi reactions

**DOI:** 10.3762/bjoc.19.53

**Published:** 2023-05-26

**Authors:** Oleksandr V Kolomiiets, Alexander V Tsygankov, Maryna N Kornet, Aleksander A Brazhko, Vladimir I Musatov, Valentyn A Chebanov

**Affiliations:** 1 Division of Chemistry of Functional Materials, State Scientific Institution “Institute for Single Crystals” of National Academy of Sciences of Ukraine, Nauky Ave., 60, 61072, Kharkiv, Ukraine; 2 Faculty of Chemistry, V. N. Karazin Kharkiv National University, Svobody sq., 4, 61022, Kharkiv, Ukraine; 3 National Technical University "Kharkiv Polytechnic Institute", Kyrpychova st., 2, Kharkiv, 61002, Ukraine; 4 Laboratory of Biotechnology of Physiologically Active Substances, Zaporizhzhya National University, Zhukovsky str., 66, Zaporizhzhya, 69600, Ukraine

**Keywords:** Groebke–Blackburn–Bienaymé reaction, imidazo[1,2-*a*]pyridine, isocyanide, multicomponent reaction, peptidomimetic, Ugi reaction

## Abstract

Peptidomimetics with a substituted imidazo[1,2-*a*]pyridine fragment were synthesized by a tandem of Groebke–Blackburn–Bienaymé and Ugi reactions. The target products contain substituted imidazo[1,2-*a*]pyridine and peptidomimetic moieties as pharmacophores with four diversity points introduced from readily available starting materials, including scaffold diversity. A small focused compound library of 20 Ugi products was prepared and screened for antibacterial activity.

## Introduction

**Scheme 1:**
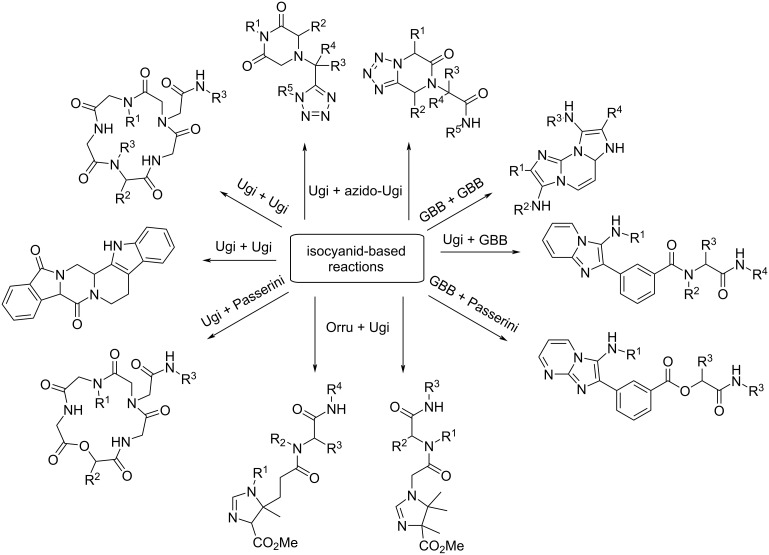
Diversity of structures synthesized by combining IMCR’s.

The use of isocyanide multicomponent reactions (IMCR) to prepare biologically active compounds is one of the most promising tools in modern organic and medicinal chemistry. Therefore, isocyanide multicomponent reactions are the way to prepare the necessary drugs "of tomorrow", and the possibility to combine such reactions gives a powerful method to rapidly and qualitatively expand the molecular diversity of biologically active compounds. The following possible combinations of IMCR have been described till today: Ugi and Ugi, azido-Ugi and Ugi, Ugi and Passerini, Groebke–Blackburn–Bienaymé (GBB-3CR) and Ugi, GBB-3CR and GBB-3CR, GBB-3CR and Passerini, Orru and Ugi, Orru and Passerini [1] (Scheme 1).

However, the issue of whether certain isocyanide multicomponent reactions can be combined is still unresolved, particularly due to the potential for varying the initial compounds involved.

**Figure 1:**
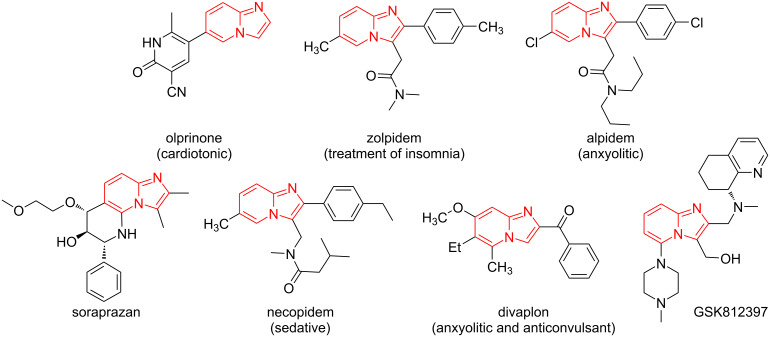
Drugs possessing imidazo[1,2-*a*]pyridine unit.

Among all IMCRs, the Groebke–Blackburn–Bienaymé reaction is noteworthy [2–4]. The compounds obtained by this transformation demonstrate a wide range of biological activity, in particular structures containing a substituted imidazo[1,2-*a*]pyridine fragment, such as anticancer [5], antibacterial [6], antifungal [7], antiviral [8], anti-inflammatory [9], antimalarial [10], antiparkinsonian [11] and antituberculous activities [12]. In addition, some derivatives show enzyme inhibition [13] and can be used as potential drug candidate against Alzheimer's disease [14]. Compounds containing the imidazo[1,2-*a*]pyridine moiety are present in many natural products and marketed drugs, e.g., alpidem (an anxiolytic) [15], necopidem (anxiolytic) [16], zolpidem (hypnotic for the treatment of insomnia) [17], olprinon (cardiotonic agent for the treatment of acute heart failure) [18], GSK812397 (with anti-human immunodeficiency virus (HIV) properties) [19] (Figure 1).

**Figure 2:**
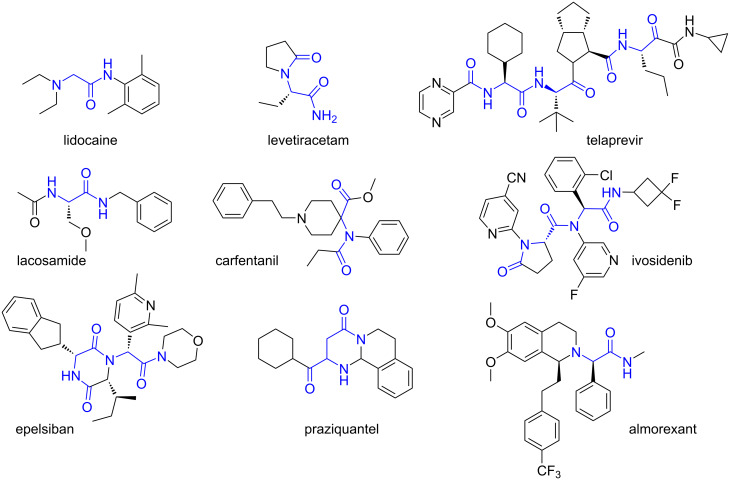
Drugs possessing peptide unit.

In light of the numerous viral epidemics and even pandemics, antiviral drugs that can inhibit the activity of proteins and enzymes encoded by viruses, including influenza, SARS-CoV, and SARS-CoV-2, are becoming extremely important. For example, peptidomimetic structures such as lopinavir and ritonavir can inhibit 3CL protease, which is one of the major protein structures encoded by the SARS-CoV-2 genome [20]. Therefore, it is necessary to pay attention to another isocyanide multicomponent reaction – the Ugi reaction [21], which is one of the most important methods for the synthesis not only of peptidomimetics but also of other types of complex organic compounds. The presence of several diversity points and modification of starting components for a given reaction makes it possible to very quickly not only to insert important pharmacophoric fragments, but also to create a wide chemical space for the search for lead structures to solve problems of medicinal chemistry. Examples of drugs synthesized by MCR clearly demonstrate the immense advantages in this context, e.g., lidocaine [22], praziquantel [23–24], telaprevir [25], lacosamide [26], carfentanil [27], ivosidenib [28], and levetiracetam [29] (Figure 2). Epelsiban [30] and almorexant [31] are examples of agents currently or recently in clinical trials that have been synthesized using the MCR repertoire.

Therefore, an urgent problem is to synthesize structurally complex compounds, molecular hybrids containing pharmacophoric and peptidomimetic fragments, to explore their biological activity.

**Scheme 2:**
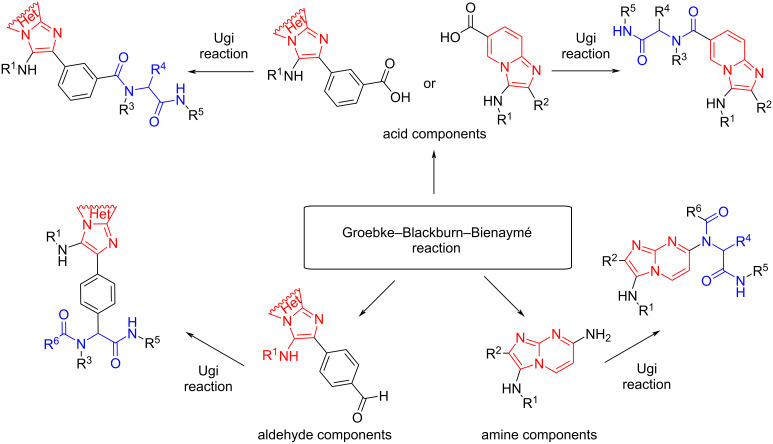
Diversity of GBB reaction products as precursors for Ugi reaction.

The possibility of inserting an unsubstituted imidazo[1,2-*a*]pyridine fragment into the peptidomimetic chain was already described in 2010 [32]. Thus, the corresponding acid components were synthesized with GBB-3CR and used in Ugi and Paserini reactions with various aldehydes, amines (for Ugi) and isocyanides. Moreover, in 2016 [33], an alternative route to use GBB-3CR products in Ugi reaction as carbonyl component was proposed, but these aldehydes did not have the structure of imidazo[1,2-*a*]pyridine. Interestingly, in 2019 [1], the synthesis of the amine component using GBB-3CR and the modification of the imidazo-pyrimidine scaffold by a peptidomimetic chain was carried out using the Ugi reaction (Scheme 2).

It is obvious that the exploration of the possibility to create hybrid molecules by combining GBB-3CR and Ugi is successful, so one of the promising areas is to increase the diversity of relevant compounds using substituted aminopyridines in GBB-3CR and the synthesis of new peptidomimetics.

Taking into account all these facts, we solved the task of developing a new approach for the synthesis of hybrid molecules containing substituted pharmacophoric heterocyclic and peptidomimetic fragments. An approach based on the preparation of an imidazo[1,2-*a*]pyridine-containing heterocyclic acid and its introduction as an acid component in the Ugi reaction was chosen. The route to the synthesis of the target molecules can be divided into two stages, with GBB-3CR in the first stage and the Ugi reaction in the second stage using the product of the first stage.

## Results and Discussion

**Scheme 3:**
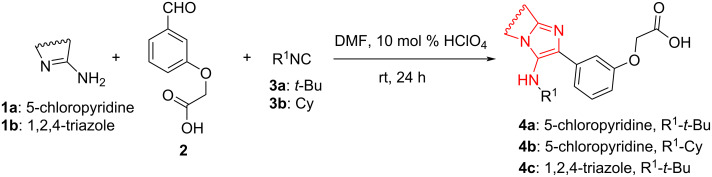
Synthesis of new acids containing a substituted imidazo[1,2-*a*]pyridine fragment.

A model reaction between 2-amino-5-chloropyridine (**1a**), 2-(3-formylphenoxy)acetic acid (**2**) and *tert*-butyl isocyanide (**3a**) was chosen for the first step (Scheme 3). In one of the previous works [34], our group has shown that the optimal conditions for this type of reactions are stirring the starting materials for 24 hours at room temperature in DMF with catalytic amounts of HClO_4_. The yield of compound **4a** in this case was 76%.

**Scheme 4:**
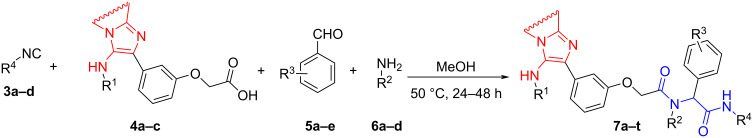
Synthesis of new peptidomimetics containing a substituted imidazo[1,2-*a*]pyridine fragment.

Then new heterocyclic acids were used as reagents for the Ugi four-component reaction. Due to the rather low solubility of the acids in methanol, it was necessary to increase the temperature and the reaction time. After a series of experiments, it was found that stirring the isocyanides **3a–d**, heterocyclic acids **4a–c**, aldehydes **5a–e**, and primary amines **6a–d** at 50 °C in methanol for 24–48 hours (depending on the nature of the starting materials) allowed obtaining the Ugi target products **7a–t** in 28–72% yield (Scheme 4, Table 1).

**Table 1:** Library of peptidomimetics **7a–t** containing a substituted imidazo[1,2-*a*]pyridine fragment.

**7**	Amidine	R^1^	R^2^	R^3^	R^4^	Yield

**a**	5-chloropyridine	*t-*Bu	4-MeOPh	H	*t-*Bu	63%
**b**	5-chloropyridine	*t-*Bu	4-MeOPh	4-Cl	*t-*Bu	71%
**c**	5-chloropyridine	*t-*Bu	4-MeOPh	4-MeO	*t-*Bu	61%
**d**	5-chloropyridine	*t-*Bu	4-MeOPh	methylenedioxy	*t-*Bu	72%
**e**	5-chloropyridine	*t-*Bu	4-MeOPh	4-CF_3_	*t-*Bu	45%
**f**	5-chloropyridine	*t-*Bu	4-MeOPh	4-Cl	Cy	51%
**g**	5-chloropyridine	*t-*Bu	4-MeOPh	4-MeO	Cy	56%
**h**	5-chloropyridine	*t-*Bu	4-MeOPh	methylenedioxy	Cy	58%
**i**	5-chloropyridine	*t-*Bu	4-MeOPh	4-CF_3_	Cy	62%
**j**	5-chloropyridine	*t-*Bu	4-MeOPh	4-Cl	ethyloxycarbonyl	61%
**k**	5-chloropyridine	*t-*Bu	4-MeOPh	4-OMe	ethyloxycarbonyl	40%
**l**	5-chloropyridine	*t-*Bu	4-MeOPh	methylenedioxy	ethyloxycarbonyl	28%
**m**	5-chloropyridine	*t-*Bu	4-MeOPh	4-Cl	*o*-NO_2_Bn	53%
**n**	5-chloropyridine	*t-*Bu	4-MeOPh	4-OMe	*o*-NO_2_Bn	43%
**o**	5-chloropyridine	*t-*Bu	Ph	4-Cl	*t-*Bu	63%
**p** ^a^	5-chloropyridine	*t-*Bu	5-methylisoxazol-3-yl	4-Cl	*t-*Bu	30%
**q**	5-chloropyridine	*t-*Bu	5-methylisoxazol-3-yl	4-OMe	*t-*Bu	37%
**r**	5-chloropyridine	Cy	4-MeOPh	4-Cl	*t-*Bu	54%
**s**	5-chloropyridine	Cy	4-MeOPh	4-MeO	*t-*Bu	46%
**t**	1,2,4-triazole	*t-*Bu	4-MeOPh	4-Cl	*t-*Bu	30%

^a^Ugi 3-CR reaction – azomethine was inserted into the reaction.

Having obtained several new compounds containing substituted imidazo[1,2-*a*]pyridine and peptidomimetic fragments linked by a CH_2_O linker, we further explored the possibility of synthesizing similar hybrids without a CH_2_O linker between the two moieties. The heterocyclic acid **8a** was obtained by a three-component reaction of 2-amino-5-chloropyridine (**1a**), 4-formylbenzoic acid (**5f**), and *tert*-butyl isocyanide (**3a**) according to the procedure described above (Scheme 3). In another step, we attempted to introduce the acid **8a** into the Ugi four-component reaction with 4-chlorobenzaldehyde (**5b**), 4-methoxyaniline (**6a**) and *tert*-butyl isocyanide (**3a**). It should be especially noted that the solubility of compound **8a** is very low (soluble in DMSO and *N*-methyl-2-pyrrolidone (NMP), slightly soluble in methanol, 1- and 2-propanol, acetone, DMF and insoluble in water, ethanol, acetonitrile, chloroform and some other typical solvents). Therefore, it was not surprising to us that the usual stirring of the starting materials in methanol at 50 °C was not successful for this multicomponent reaction. Increasing the temperature to 75 °C also did not give any positive results. Therefore, we tried to use DMSO, DMF and NMP as solvents for this Ugi treatment.

**Scheme 5:**
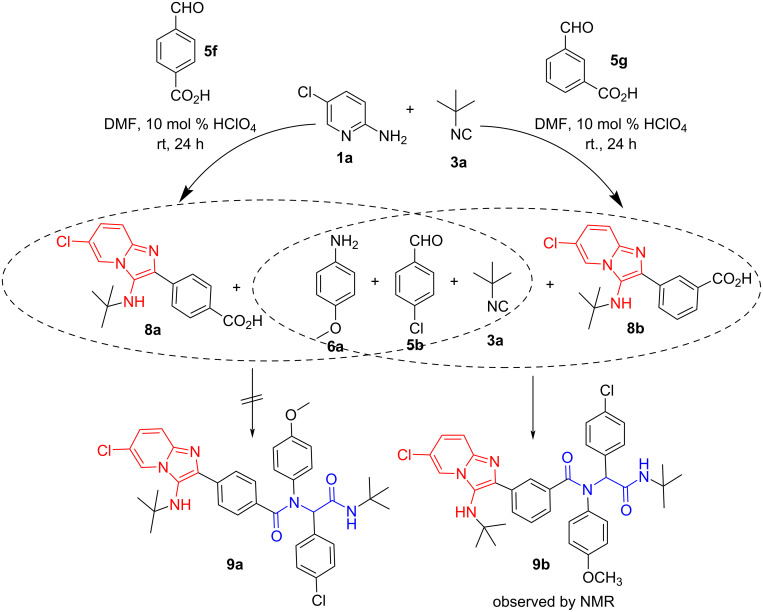
Synthesis and reactivity of new acids containing a substituted imidazo[1,2-*a*]pyridine fragment without CH_2_O linker fragment.

However, we found that the application of the solvent systems based on DMF and DMSO described in the literature [35–36] did not lead to the target compound **9a** (Scheme 5, Table 2). Stirring at elevated temperatures or reaction in NMP also proved to be inefficient.

In addition, we also tested the one-pot method for the synthesis of similar structures [32]. Aminopyridine, aldehyde and Sc(OTf)_3_ as catalyst were stirred in MeOH/DCM for 45 min at room temperature, then the isocyanide was added and the mixture was stirred for another 8 h. Without isolation, the corresponding aldehyde, amine and isocyanide were added to the resulting iminopyridine product and the mixture was then stirred at room temperature for 12 h. But again, the expected compounds could not be obtained.

Following this trend and based on published data, we synthesized heterocyclic acid **8b** (Scheme 5) containing a carboxyl group at position 3 of the phenyl ring, which we introduced into the Ugi reaction with 4-methoxyaniline (**6a**), 4-chlorobenzaldehyde (**5b**), and *tert*-butyl isocyanide (**3a**) under various conditions (Table 2). In two cases (entries 1 and 6), the formation of the target reaction product was observed by ^1^H NMR spectroscopy of the crude reaction mixture. It was found that in the case of Table 2, entry 6, the ratio of product **9b** to acid **8b** was approximately 1:3.

**Table 2:** Screening conditions for synthesis of peptidomimetic **9а** and **9b**.

Entry	Conditions	Ratio **8a**/**9a**^a^	Ratio **8b**/**9b**^a^

1^b^	MeOH/DMF 1:2, 72 h, 75 °С	only **8a**	9:1
2^b^	MeOH, 72 h, 75 °С	only **8a**	only **8b**
3	DMF/DCM 1:2, 48 h, rt	only **8a**	–
4^b^	DMSO/DCM 1:2, 48 h, rt	only **8a**	only **8b**
5	NMP, 72 h, 75 °С	only **8a**	–
6^b^	DCM/MeOH 3:2, Sc(OTf)_3_ – 10 mol %, 48 h, rt (one pot)	only **8a**	3:1

^a^Determined by ^1^H NMR; ^b^conditions that were checked for the synthesis of both compounds.

The obtained results show that for the synthesis of new compounds containing precisely substituted imidazo[1,2-*a*]pyridine and peptidomimetic fragments it is better to use acids with a linker between the carboxyl group and aryl ring. This molecular fragment increases the solubility of the whole molecule, which leads to an improvement in the reactivity of the acids, whereas the reactivity of acids **8a** and **8b** is rather low in Ugi reactions.

### Antibacterial activity

In the following experiment, we tested a specific group of compounds for their ability to act as antibacterial agents against *Bacillus subtilis* (strain 1211), *Staphylococcus aureus* (strain 2231) (Gram-positive), *Escherichia coli* (strain 1257), *Pseudomonas aeruginosa* (strain 1111) (Gram-negative). Compared to the reference substance, nitroxoline, the compounds generally showed lower levels of activity. However, some of the compounds demonstrated weak antimicrobial effects, as evidenced by their ability to inhibit the growth of the test microorganisms (see Table 3 for details).

**Table 3:** Antibacterial activity results.

Entry	Compound	MIC^a^/MBC^b^, mg/L	Strains of test cultures			

			*Escherichia coli*	*Pseudomonas aeruginosa*	*Bacillus subtilis*	*Staphylococcus aureus*

1	**7d**	MIC	500	500	500	500
		MBC	–	–	–	–
2	**7e**	MIC	500	500	500	500
		MBC	–	–	–	–
3	**7f**	MIC	–	–	–	–
		MBC	–	–	–	–
4	**7g**	MIC	–	–	250	–
		MBC	–	–	–	–
5	**7i**	MIC	500	–^c^	500	–
		MBC	–	–	–	–
6	**7o**	MIC	500	–	125	250
		MBC	–	–	–	–
7	**7p**	MIC	500	–	–	–
		MBC	–	–	–	–
8	**7q**	MIC	500	500	500	500
		MBC	–	–	–	–
9	**7t**	MIC	–	–	–	–
		MBC	–	–	–	–
10	nitroxoline	MIC	15.6	62.5	32.25	1.9
		MBC	15.6	62.5	31.25	1.9

^a^MIC – minimum inhibitory concentration; ^b^MBC – minimum bactericidal concentration; “–“ - the substance at concentration ≤ 500 mg/L does not inhibit culture growth; ^c^compounds improve the growth and development of bacteria compared to the control group.

Both Gram-positive and Gram-negative bacteria (specifically, strains of *E. coli* and *B. subtilis*) were equally inhibited in their growth. Compound **7o**, in particular, was able to inhibit the growth of *B. subtilis* at a concentration of 125 mg/L. Bacteriostatic activity against *E. coli* was only observed at higher concentrations of 500 mg/L. This was also observed with the tested *S. aureus* strain. However, the Gram-negative bacterium *P. aeruginosa* showed resistance to a large number of synthesized compounds, with some compounds even promoting its growth and development at the given concentration range. Despite the low level of antibacterial activity observed with the synthesized heterocycles, this could be beneficial for screening these compounds for other types of activity, such as anticancer or antidiabetic effects, since it would minimize any negative impact on the organism's microflora.

## Conclusion

To summarize, the study involved the combination of two multicomponent reactions – Groebke–Blackburn–Bienaymé and Ugi-type – to produce substituted 6-chloroimidazo[1,2-*a*]pyridin-2-ylphenoxy acetamides and 1*H*-imidazo[1,2-*b*][1,2,4]triazol-5-yl-phenoxy acetamide in a flexible manner. A method for the synthesis of the desired compounds was developed, and a small library of 20 Ugi products was prepared. The study also demonstrated that the use of a CH_2_O linker between the carboxyl group and the aryl ring in the Ugi reaction enhanced the reactivity of the acid component. The target compounds, which contained two pharmacophores – [1,2-*a*]pyridine (1*H*-imidazo[1,2-*b*][1,2,4]triazole) and peptidomimetic moieties – were evaluated for their antibacterial activity and exhibited a weak effect.

## Supporting Information

File 1 Experimental part.
