# Supplementary material for: Synthesis of imidazo[1,2-a]pyridine-containing peptidomimetics by tandem of Groebke–Blackburn–Bienaymé and Ugi reactions
Source: Beilstein J Org Chem. 2023 May 26;19:727–35. doi: 10.3762/bjoc.19.53 (PMC10241102; doi:10.3762/bjoc.19.53)
Supplement: File 1 — Experimental part. [file Beilstein_J_Org_Chem-19-727-s001.pdf]

BEILSTEIN JOURNAL OF ORGANIC CHEMISTRY

Supporting Information

for

Synthesis of imidazo[1,2-*a*]pyridine-containing peptidomimetics by tandem of Groebke–Blackburn–Bienaymé and Ugi reactions

Oleksandr V. Kolomiiets, Alexander V. Tsygankov, Maryna N. Kornet, Aleksander A. Brazhko, Vladimir I. Musatov and Valentyn A. Chebanov

Beilstein J. Org. Chem. **2023,** *19,* 727–735. doi:10.3762/bjoc.19.53

Experimental part

License and Terms: This is a supporting information file under the terms of the Creative Commons Attribution License (https://creativecommons.org/licenses/by/4.0). Please note that the reuse, redistribution and reproduction in particular requires that the author(s) and source are credited and that individual graphics may be subject to special legal provisions.
The license is subject to the *Beilstein Journal of Organic Chemistry* terms and conditions: (https://www.beilstein-journals.org/bjoc/terms)

Table of contents

1. General S3

2. Synthetic aspects and procedures S3

3. ^{1}H and ^{13}C NMR spectra S29

4. Measurement of antibacterial activity S54

1.General

The starting 2-(3-formylphenoxy)acetic acid (**2**) [1], *o*-nitrobenzyl isocyanide (**6d**) [2] were synthesized according to the known literature procedures. *tert*-Butyl-, cyclohexyl-, ethyl aceto-2-yl isocyanides and substituted aldehydes were commercially available. Melting points of all compounds synthesized were determined with a Kofler melting point apparatus and were uncorrected. The NMR spectra were recorded in DMSO-d_6 and $CDCl_3$ at 300 MHz (75 MHz for ^{13}C) with a Bruker Avance 300 spectrometer, at 400 MHz (100 MHz for ^{13}C) with a Varian MR-400 (Note: Some ^{1}H NMR spectra contains rotamers) Mass spectra (ESI) were recorded in both positive and negative ion detection modes on Shimadzu LCMS-2020 spectrometer.

2. Synthetic aspects and procedures

Synthesis of imidazo[1,2-*a*]containing heterocyclic acids 4a–c.

Amidine (2 mmol) and aldehyde (2 mmol) were added to a 10 ml round-bottom heat-resistant flask and dissolved in 4 mL of DMF. In addition, a 70% aqueous solution of $HClO_4$ in a catalytic amount (20 mol %) was added. Isocyanide was added to the resulting solution and left to stir for 24 hours. After 24 hours, the reaction mixture was poured onto ice, the precipitate obtained was filtered off and washed with cold methyl alcohol, then dried in a vacuum and analyzed.

2-(3-(3-(*tert*-Butylamino)-6-chloroimidazo[1,2-*a*]pyridin-2-yl)phenoxy)acetic acid (4a). Yield 76%, yellowish solid. T_m = 252-253 ^{o}C. ^{1}H NMR (400 MHz, DMSO-d$_6$) δ 8.48 (s, 1H), 7.77 – 7.63 (m, 2H), 7.50 (d, J = 9.5 Hz, 1H), 7.28 (t, J = 8.0 Hz, 1H), 7.18 (dd, J = 9.5, 2.0 Hz, 1H), 6.81 (dd, J = 8.3, 2.7 Hz, 1H), 4.69 (s, 3H), 0.97 (s, 9H); ^{13}C NMR (100 MHz, DMSO-d$_6$) δ 167.5, 158.5, 141.1, 136.5, 135.5, 131.5, 130.6, 128.5, 128.1, 127.7, 124.3, 124.2, 116.6, 111.2, 55.8, 39.9, 39.9, 39.7, 39.6, 39.5, 39.5, 39.4, 39.3, 39.1, 38.9, 30.1, 13.9.

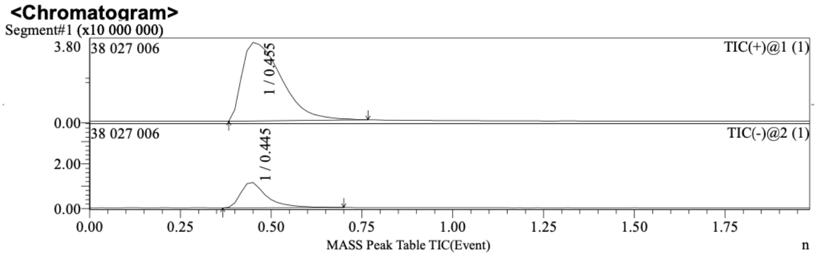

TIC(1-1)

Peak#	Ret. Time	m/z	Area	Compound Name	A/H	Event#	Area%	Height%
1	0.455	TIC	266082446		7.542	1-1	100.000	100.000
Total			266082446				100.000	100.000

TIC(1-2)

Peak#	Ret. Time	m/z	Area	Compound Name	A/H	Event#	Area%	Height%
1	0.445	TIC	56940259		5.035	1-2	100.000	100.000
Total			56940259				100.000	100.000

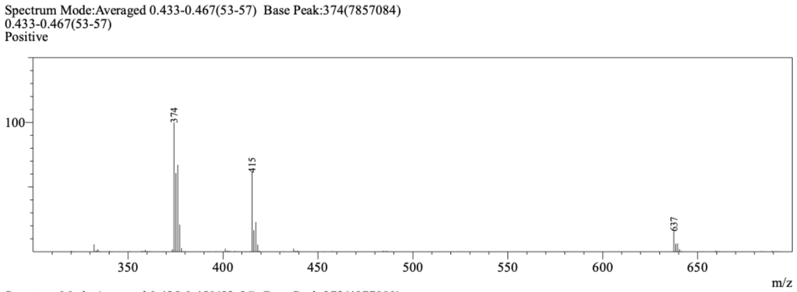

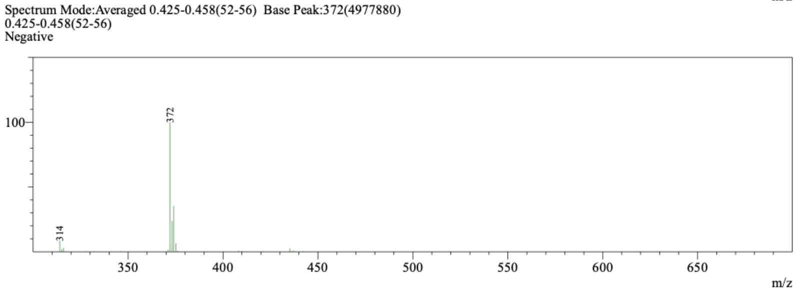

2-(3-(6-Chloro-3-(cyclohexylamino)imidazo[1,2-*a*]pyridin-2-yl)phenoxy)acetic acid (4b). Yield 78%, yellowish solid. T_m = 248-249 ^{o}C. ^{1}H NMR (400 MHz, DMSO-d_6) δ 8.45 (d, J = 2.1 Hz, 1H), 7.79 (d, J = 7.8 Hz, 1H), 7.72 (s, 1H), 7.49 (d, J = 9.4 Hz, 1H), 7.31 (t, J = 8.0 Hz, 1H), 7.16 (dd, J = 9.5, 2.0 Hz, 1H), 6.81 (dd, J = 8.1, 2.7 Hz, 1H), 4.82 (d, J = 6.5 Hz, 1H), 4.69 (s, 2H), 2.83 (d, J = 15.6 Hz, 1H), 1.73 – 1.54 (m, 4H), 1.46 (s, 1H), 1.24 (d, J = 11.9 Hz, 2H), 1.06 (s, 3H); ^{13}C NMR (100 MHz, DMSO-d_6) δ 170.2, 157.9, 138.6, 135.6, 135.0, 129.3, 126.7, 124.5, 121.0, 119.3, 118.7, 117.7, 113.6, 111.9, 64.4, 56.5, 40.1, 39.9, 39.9, 39.8, 39.7, 39.6, 39.6, 39.5, 39.4, 39.3, 39.1, 38.9, 33.4, 25.3, 24.5.

<Chromatogram>

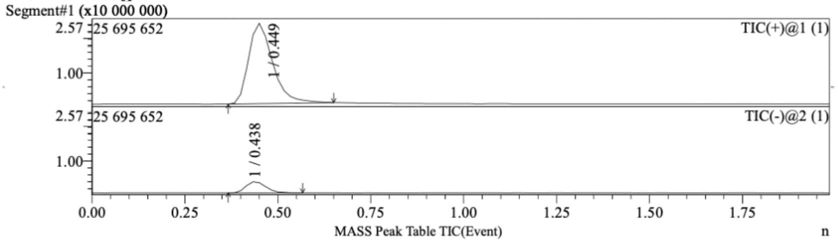

TIC(1-1)

Peak#	Ret. Time	m/z	Area	Compound Name	A/H	Event#	Area%	Height%
1	0.449	TIC	105169257		4.522	1-1	100.000	100.000
Total			105169257				100.000	100.000

TIC(1-2)

Peak#	Ret. Time	m/z	Area	Compound Name	A/H	Event#	Area%	Height%
1	0.438	TIC	11933967		3.731	1-2	100.000	100.000
Total			11933967				100.000	100.000

Spectrum Mode:Averaged 0.417-0.450(51-55) Base Peak:400(4699156)
0.417-0.450(51-55)
Positive

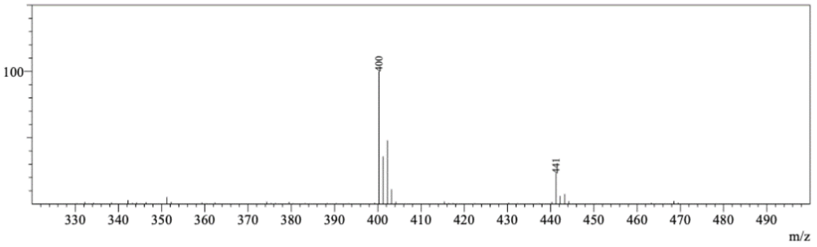

2-(3-(6-(*tert*-Butylamino)-1*H*-imidazo[1,2-*b*][1,2,4]triazol-5-yl)phenoxy)acetic acid (4c). Yield 78%, yellowish solid. T_m = 209-210 ^oC. ^1H NMR (400 MHz, DMSO-d_6) δ 7.78 (s, 1H), 7.56 (d, J = 6.9 Hz, 2H), 7.29 (t, J = 8.1 Hz, 1H), 6.84 – 6.75 (m, 1H), 4.68 (s, 2H), 1.08 (s, 9H); ^{13}C NMR (100 MHz, DMSO-d_6) δ 170.6, 158.3, 153.6, 148.5, 132.4, 129.9, 123.9, 121.3, 119.3, 113.9, 112.3, 64.9, 54.9, 40.6, 40.4, 40.2, 40.2, 39.9, 39.8, 39.6, 39.3, 30.6, 28.7.

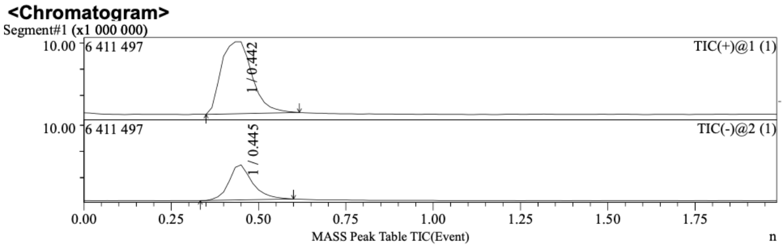

MASS Peak Table TIC(Event)

TIC(1-1)

Peak#	Ret. Time	m/z	Area	Compound Name	A/H	Event#	Area%	Height%
1	0.442	TIC	33943446		6.182	1-1	100.000	100.000
Total			33943446				100.000	100.000

TIC(1-2)

Peak#	Ret. Time	m/z	Area	Compound Name	A/H	Event#	Area%	Height%
1	0.445	TIC	12639407		4.734	1-2	100.000	100.000
Total			12639407				100.000	100.000

Spectrum Mode:Averaged 0.417-0.450(51-55) Base Peak:371(605797)
0.417-0.450(51-55)
Positive

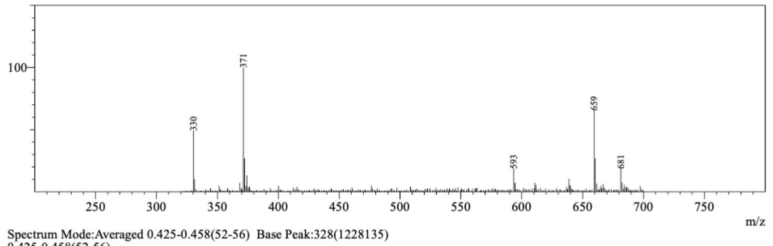

Spectrum Mode:Averaged 0.425-0.458(52-56) Base Peak:328(1228135)
0.425-0.458(52-56)
Negative

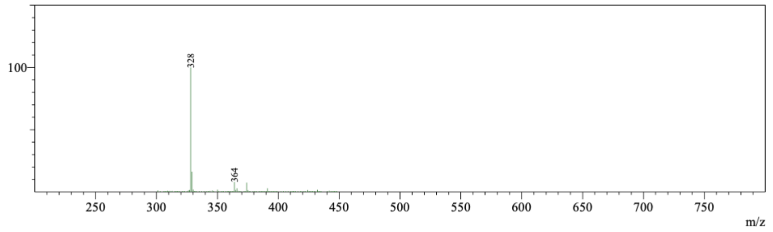

General procedure for the synthesis of peptidomimetics 7a–t.

Amine (0.55 mmol), aldehyde (0.55 mmol), and 1 mL of MeOH were added to a 5 mL capped vial. The resulting solution was stirred for 1 hour. After that, imidazo[1,2-*a*]-containing heterocyclic acid (0.5 mmol) and isocyanide (0.55 mmol) were added. The reaction mixture in a screw cap vial was hermetically closed and placed in an oil bath (50 °C) for 24–48 hours. Then the mixture was cooled for 30 minutes at a temperature of −15 °C. The cooled solution was stirred at room temperature until a precipitate formed. The resulting precipitate was filtered, washed with a cold solution of MeOH, and dried in air. The dried precipitate was recrystallized from hexane and filtered again, then dried in a vacuum and analyzed.

***N*-(*tert*-Butyl)-2-(2-(3-(3-(*tert*-butylamino)-6-chloroimidazo[1,2-*a*]pyridin-2-yl)phenoxy)-*N*-(4-methoxyphenyl)acetamido)-2-phenylacetamide (7a).** Yield 63%, white solid. T_m = 131-134 ^oC. ^1H NMR (400 MHz, $CDCl_3$) δ 8.25 (d, J = 2.0 Hz, 1H), 7.45 (t, J = 9.6 Hz, 2H), 7.28 (dd, J = 15.8, 7.9 Hz, 3H), 7.22 – 7.03 (m, 6H), 6.96 – 6.47 (m, 3H), 5.99 (s, 1H), 5.53 (s, 1H), 4.40 (d, J = 2.9 Hz, 2H), 3.72 (s, 3H), 1.24 (s, 9H), 1.00 (s, 9H); ^{13}C NMR (100 MHz, $CDCl_3$) δ 168.6, 168.5, 159.4, 158.2, 134.2, 131.4, 130.6, 130.5, 129.5, 128.4, 128.4, 124.5, 121.7, 121.4, 116.9, 115.3, 114.5, 114.2, 77.3, 76.9, 76.7, 66.9, 65.6, 56.5, 55.3, 51.6, 30.3, 28.5.

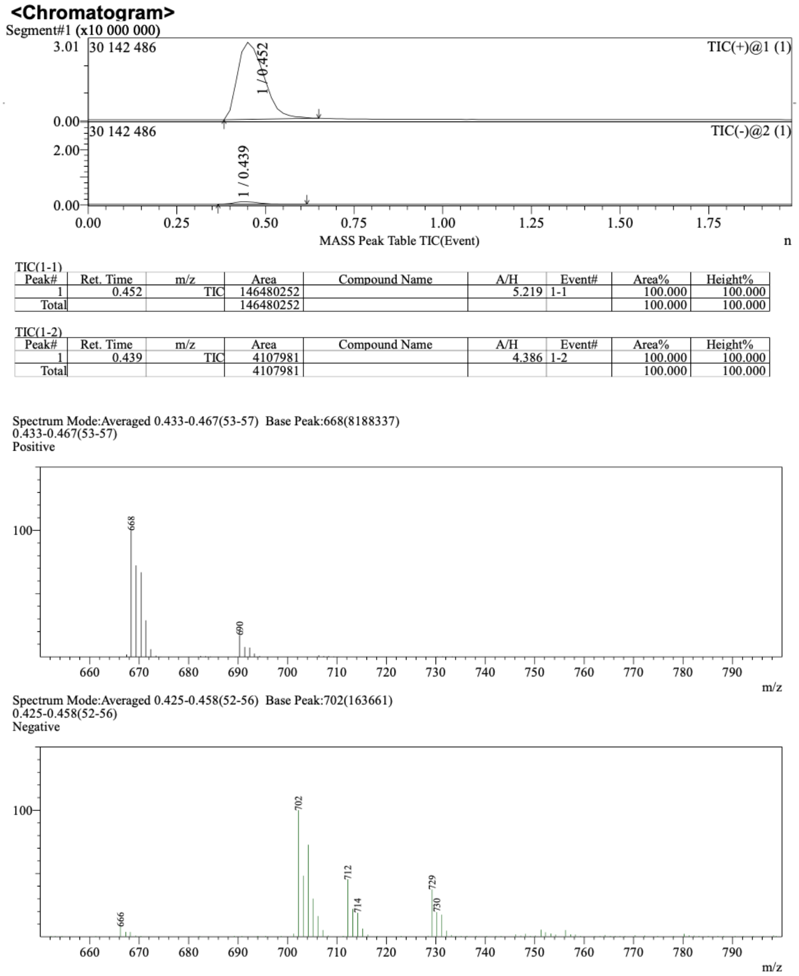

TIC(1-1)

Peak#	Ret. Time	m/z	Area	Compound Name	A/H	Event#	Area%	Height%
1	0.452	TIC	146480252		5.219	1-1	100.000	100.000
Total			146480252				100.000	100.000

TIC(1-2)

Peak#	Ret. Time	m/z	Area	Compound Name	A/H	Event#	Area%	Height%
1	0.439	TIC	4107981		4.386	1-2	100.000	100.000
Total			4107981				100.000	100.000

***N*-(*tert*-butyl)-2-(2-(3-(3-(*tert*-butylamino)-6-chloroimidazo[1,2-*a*]pyridin-2-yl)phenoxy)-*N*-(4-methoxyphenyl)acetamido)-2-(4-chlorophenyl)acetamide (7b).** Yield 71%, white solid. T_m = 122-123 ^oC. ^1H NMR (400 MHz, DMSO-d_6) δ 8.50 (s, 1H),

7.86 – 7.62 (m, 3H), 7.57 (s, 1H), 7.47 (d, J = 9.4 Hz, 1H), 7.29 – 7.11 (m, 4H), 7.04 (d, J = 8.2 Hz, 2H), 6.68 (d, J = 8.4 Hz, 4H), 5.98 (s, 1H), 4.61 (s, 1H), 4.38 (d, J = 15.2 Hz, 1H), 4.26 (d, J = 15.3 Hz, 1H), 3.65 (s, 3H), 1.14 (s, 9H), 0.95 (s, 9H); ^{13}C NMR (100 MHz, DMSO-d_6) δ 168.9, 167.7, 159.0, 158.1, 139.8, 139.2, 136.6, 134.8, 132.7, 132.2, 130.7, 129.3, 128.2, 125.3, 125.2, 122.2, 120.9, 118.9, 118.1, 114.2, 113.9, 66.4, 63.6, 56.4, 55.6, 50.8, 40.6, 40.4, 40.2, 40.2, 40.0, 39.9, 39.8, 39.5, 39.3, 30.5, 28.7.

<Chromatogram>

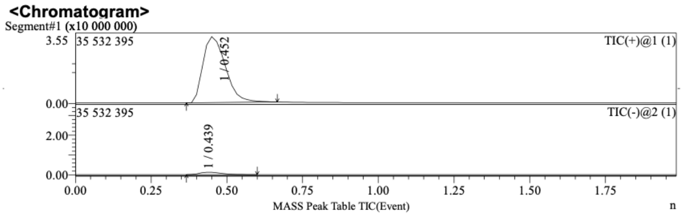

MASS Peak Table TIC(Event)

TIC(1-1)

Peak#	Ret. Time	m/z	Area	Compound Name	A/H	Event#	Area%	Height%
1	0.452	TIC	171592920		5.165	1-1	100.000	100.000
Total			171592920				100.000	100.000

TIC(1-2)

Peak#	Ret. Time	m/z	Area	Compound Name	A/H	Event#	Area%	Height%
1	0.439	TIC	5501845		4.618	1-2	100.000	100.000
Total			5501845				100.000	100.000

Spectrum Mode:Averaged 0.433-0.467(53-57) Base Peak:702(7824642)
0.433-0.467(53-57)
Positive

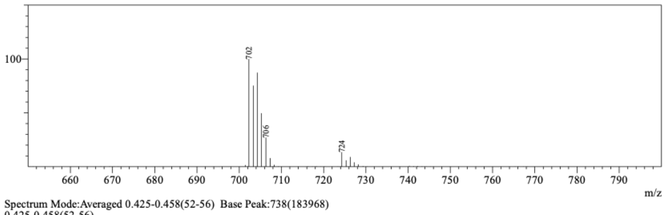

Spectrum Mode:Averaged 0.425-0.458(52-56) Base Peak:738(183968)
0.425-0.458(52-56)
Negative

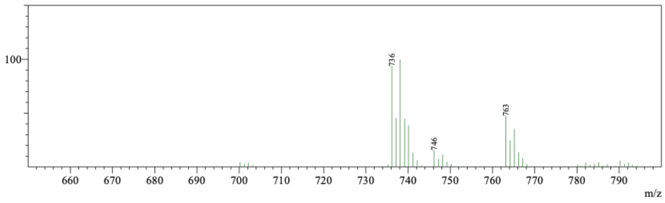

***N*-(*tert*-Butyl)-2-(2-(3-(3-(*tert*-butylamino)-6-chloroimidazo[1,2-*a*]pyridin-2-yl)phenoxy)-*N*-(4-methoxyphenyl)acetamido)-2-(4-methoxyphenyl)acetamide (7c).** Yield 61%, white solid. T_m = 114-115 ^oC. ^1H NMR (400 MHz, $CDCl_3$) δ 8.26 (s, 1H), 7.45 (dd, J = 13.1, 8.4 Hz, 2H), 7.38 – 7.21 (m, 3H), 7.04 (dd, J = 29.6, 8.9 Hz, 3H), 6.73 (dd, J = 43.9, 8.7 Hz, 6H), 5.97 (s, 1H), 5.52 (s, 1H), 4.38 (d, J = 2.7 Hz, 2H), 3.73 (d, J = 5.3 Hz, 6H), 1.24 (s, 9H), 1.00 (s, 9H); ^{13}C NMR (100 MHz, DMSO-d_6) δ

169.8, 158.9, 158.2, 139.8, 139.3, 136.7, 131.8, 129.4, 127.6, 125.3, 125.2, 122.3, 120.9, 118.9, 118.2, 114.3, 113.9, 113.7, 66.4, 63.8, 56.5, 55.6, 55.4, 50.7, 49.1, 40.6, 40.4, 40.2, 40.0, 40.0, 39.8, 39.6, 39.4, 30.5, 28.8.

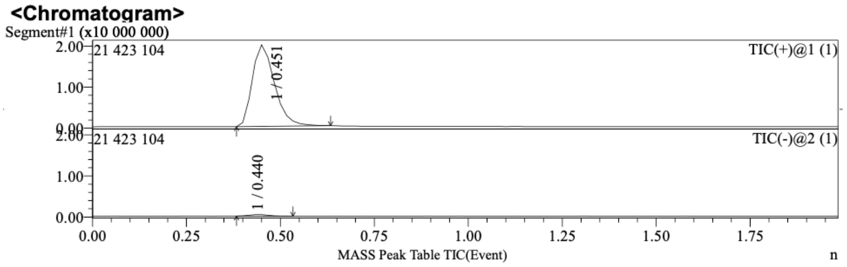

TIC(1-1)

Peak#	Ret. Time	m/z	Area	Compound Name	A/H	Event#	Area%	Height%
1	0.451	TIC	81917581		4.118	1-1	100.000	100.000
Total			81917581				100.000	100.000

TIC(1-2)

Peak#	Ret. Time	m/z	Area	Compound Name	A/H	Event#	Area%	Height%
1	0.440	TIC	1456110		3.643	1-2	100.000	100.000
Total			1456110				100.000	100.000

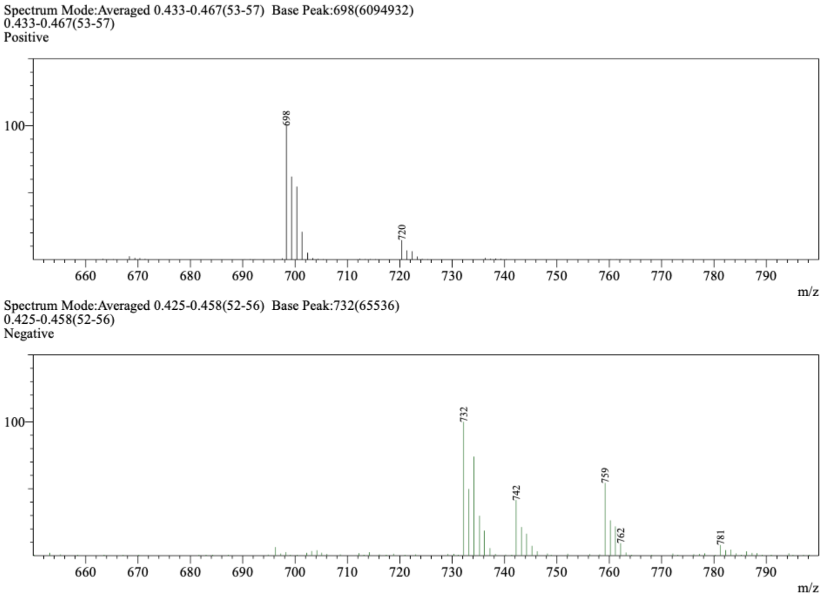

2-(Benzo[*d*][1,3]dioxol-5-yl)-*N*-(*tert*-butyl)-2-(2-(3-(3-(*tert*-butylamino)-6-chloroimidazo[1,2-*a*]pyridin-2-yl)phenoxy)-*N*-(4-methoxyphenyl)acetamido)acetamide (7d). Yield 72%, white solid. T_m = 125-126 ^oC. ^1H NMR (400 MHz, $CDCl_3$) δ 8.27 (s, 1H), 7.46 (t, J = 10.5 Hz, 2H), 7.26 (d, J = 13.9 Hz, 3H), 7.09 (d, J = 9.5 Hz, 1H), 6.99 – 6.56 (m, 6H), 5.88 (dd, J = 4.0, 1.7 Hz, 3H), 5.56 (s, 1H), 4.38 (s, 2H), 3.75 (s, 3H), 1.25 (s, 9H), 1.01 (s, 9H); ^{13}C NMR (100 MHz, $CDCl_3$) δ 168.6, 168.5, 159.5, 158.2, 147.6, 147.5, 131.4, 130.5, 129.5, 127.8,

124.4, 124.3, 121.7, 121.4, 117.1, 115.2, 114.4, 114.3, 110.6, 108.0, 101.1, 77.3, 76.9, 76.7, 66.9, 65.2, 56.5, 55.4, 51.6, 30.3, 28.5.

<Chromatogram>

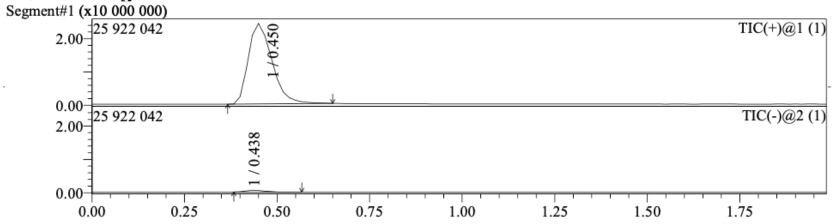

MASS Peak Table TIC(Event)

TIC(1-1)

Peak#	Ret. Time	m/z	Area	Compound Name	A/H	Event#	Area%	Height%
1	0.450	TIC	107397688		4.444	1-1	100.000	100.000
Total			107397688				100.000	100.000

TIC(1-2)

Peak#	Ret. Time	m/z	Area	Compound Name	A/H	Event#	Area%	Height%
1	0.438	TIC	1909912		3.812	1-2	100.000	100.000
Total			1909912				100.000	100.000

Spectrum Mode:Averaged 0.417-0.450(51-55) Base Peak:712(6190591)
0.417-0.450(51-55)
Positive

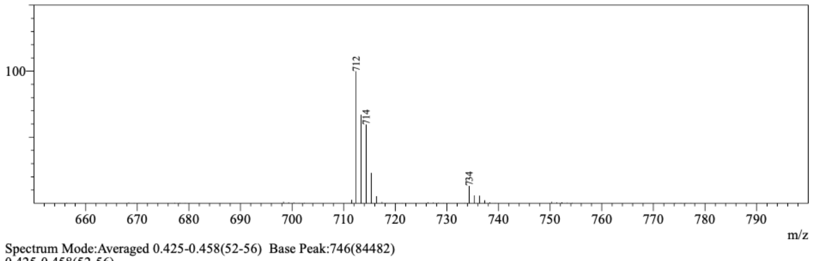

Spectrum Mode:Averaged 0.425-0.458(52-56) Base Peak:746(84482)
0.425-0.458(52-56)
Negative

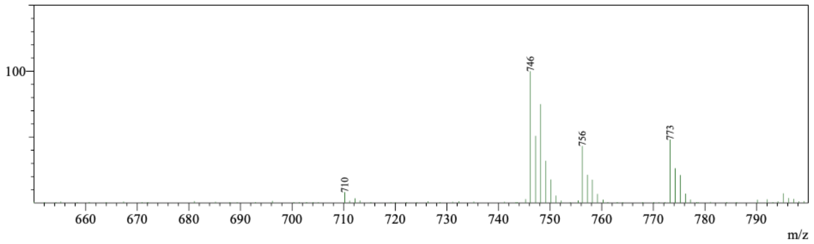

***N*-(*tert*-butyl)-2-(2-(3-(3-(*tert*-butylamino)-6-chloroimidazo[1,2-*a*]pyridin-2-yl)phenoxy)-*N*-(4-methoxyphenyl)acetamido)-2-(4-(trifluoromethyl)phenyl)acetamide (7e).** Yield 45%, white solid. T_m = 155-156 °C. ^{1}H NMR (400 MHz, $CDCl_3$) δ 8.27 (s, 1H), 7.56 – 7.38 (m, 5H), 7.29 (dd, J = 23.4, 15.7 Hz, 5H), 7.11 (d, J = 9.5 Hz, 1H), 6.89 – 6.57 (m, 3H), 6.03 (s, 1H), 5.78 (s, 1H), 4.42 (s, 2H), 3.74 (s, 3H), 1.26 (s, 9H), 1.01 (s, 9H); ^{13}C NMR (100 MHz, DMSO-d_6) δ 167.9, 167.4, 158.6, 158.5, 157.6, 140.3, 139.3, 138.7, 136.1, 131.7, 130.6, 130.2, 128.8, 124.8, 124.7, 124.6, 121.7, 120.4, 118.4, 117.7, 113.8, 113.3, 65.8, 63.5, 55.9, 55.1,

50.4, 40.1, 39.9, 39.9, 39.8, 39.8, 39.7, 39.6, 39.5, 39.4, 39.3, 39.2, 38.9, 30.9, 29.9, 28.2, 22.0, 13.9.

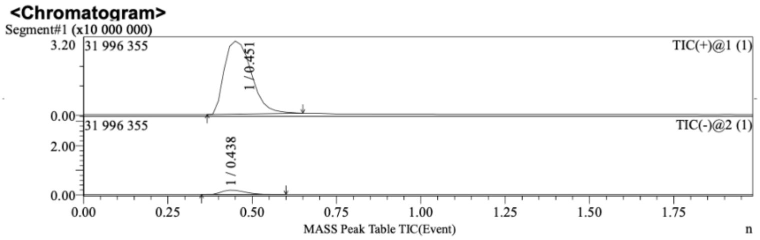

TIC(1-1)

Peak#	Ret. Time	m/z	Area	Compound Name	A/H	Event#	Area%	Height%
1	0.451	TIC	166950476		5.597	1-1	100.000	100.000
Total			166950476				100.000	100.000

TIC(1-2)

Peak#	Ret. Time	m/z	Area	Compound Name	A/H	Event#	Area%	Height%
1	0.438	TIC	8080163		4.641	1-2	100.000	100.000
Total			8080163				100.000	100.000

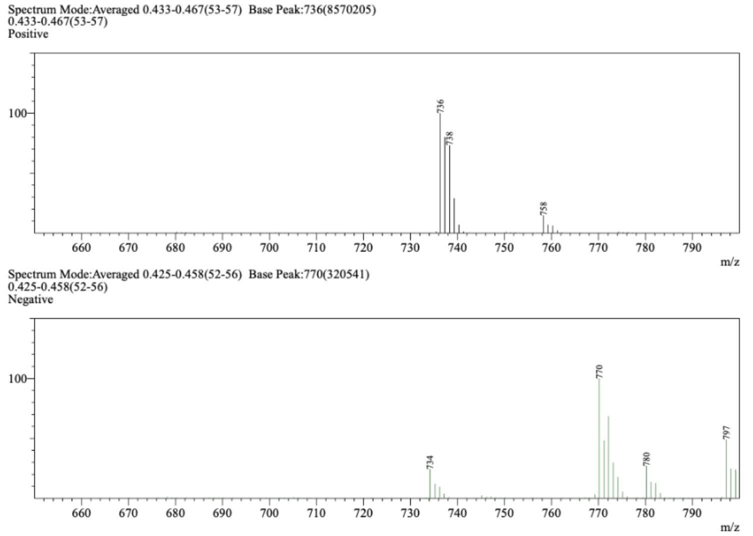

2-(3-(3-(*tert*-Butylamino)-6-chloroimidazo[1,2-*a*]pyridin-2-yl)phenoxy)-*N*-(1-(4-chlorophenyl)-2-(cyclohexylamino)-2-oxoethyl)-*N*-(4-methoxyphenyl)acetamide (7f). Yield 51%, white solid. T_m = 154-155 ^oC. ^1H NMR (400 MHz, $CDCl_3$) δ 8.23 (s, 1H), 7.42 (t, J = 7.7 Hz, 3H), 7.33 – 7.22 (m, 3H), 7.11 (q, J = 7.5 Hz, 6H), 6.95 – 6.47 (m, 5H), 6.21 (s, 1H), 4.40 (s, 2H), 4.13 (dd, J = 7.0, 2.7 Hz, 2H), 4.01 (d, J = 5.5 Hz, 3H), 3.74 (d, J = 3.0 Hz, 4H), 1.30 – 1.15 (m, 6H), 0.98 (d, J = 3.0 Hz, 13H), 0.85 (dd, J = 10.5, 6.6 Hz, 5H); ^{13}C NMR (100 MHz, DMSO-d_6) δ 167.9, 167.4, 158.6 158.5, 157.6, 139.3, 138.7, 136.1, 134.2, 132.3, 131.8, 130.2, 128.8, 127.8, 124.8, 124.7, 121.8, 120.5, 118.4, 117.7, 113.8, 113.6, 113.4, 65.8, 62.8, 55.9, 55.1, 48.6, 47.9,

40.1, 39.9, 39.9, 39.8, 39.7, 39.7, 39.6, 39.5, 39.4, 39.3, 39.2, 38.9, 32.1, 32.0, 29.9, 25.1, 24.5, 24.4.

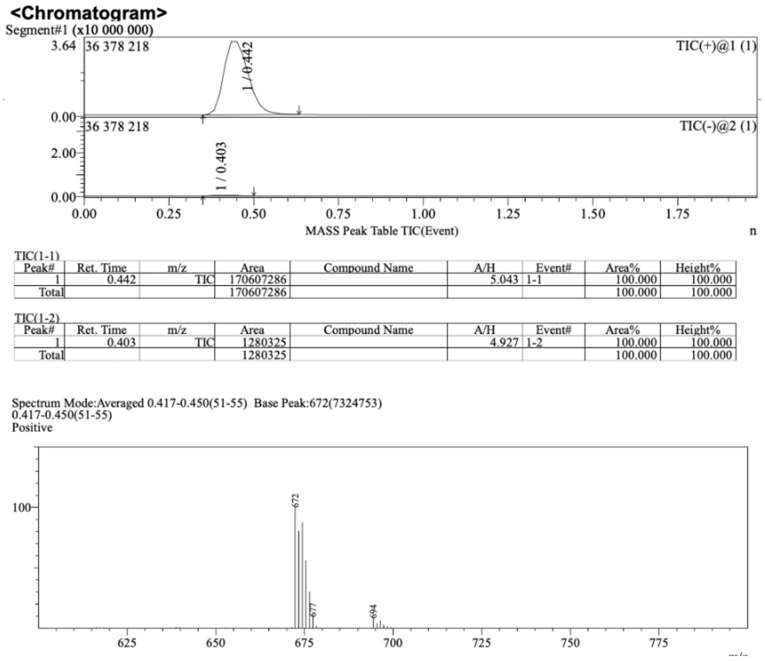

2-(3-(3-(*tert*-Butylamino)-6-chloroimidazo[1,2-*a*]pyridin-2-yl)phenoxy)-*N*-(2-(cyclohexylamino)-1-(4-methoxyphenyl)-2-oxoethyl)-*N*-(4-methoxyphenyl)acetamide (7g). Yield 56%, white solid. T_m = 116-117 °C. ^1H NMR (400 MHz, $CDCl_3$) δ 8.26 (s, 1H), 7.43 (t, J = 8.9 Hz, 3H), 7.26 (d, J = 10.8 Hz, 3H), 7.07 (d, J = 9.5 Hz, 1H), 6.99 (d, J = 8.2 Hz, 2H), 6.73 (dd, J = 42.9, 8.3 Hz, 6H), 6.09 (s, 1H), 5.53 (d, J = 8.1 Hz, 1H), 4.37 (s, 2H), 3.73 (s, 9H), 1.95 – 1.72 (m, 4H), 1.27 (s, 3H), 0.99 (d, J = 2.0 Hz, 16H); ^{13}C NMR (100 MHz, DMSO-d_6) δ 168.7, 167.2, 158.5, 158.4, 157.7, 139.3, 138.7, 136.1, 131.8, 131.3, 130.4, 128.9, 126.9, 124.8, 124.7, 121.8, 120.4, 118.4, 117.7, 113.6, 113.5, 113.2, 65.9, 62.9, 55.9, 55.1, 54.9, 48.6, 47.9, 40.1, 39.9, 39.9, 39.8, 39.7, 39.7, 39.6, 39.5, 39.4, 39.3, 39.2, 38.9, 32.2, 32.1, 29.9, 25.1, 24.6, 24.4.

<Chromatogram>

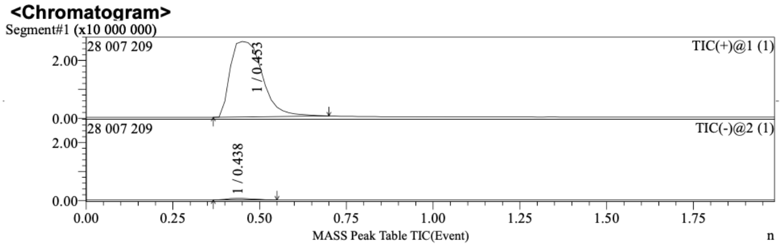

TIC(1-1)

Peak#	Ret. Time	m/z	Area	Compound Name	A/H	Event#	Area%	Height%
1	0.453	TIC	167896719		6.431	1-1	100.000	100.000
Total			167896719				100.000	100.000

TIC(1-2)

Peak#	Ret. Time	m/z	Area	Compound Name	A/H	Event#	Area%	Height%
1	0.438	TIC	2626902		4.978	1-2	100.000	100.000
Total			2626902				100.000	100.000

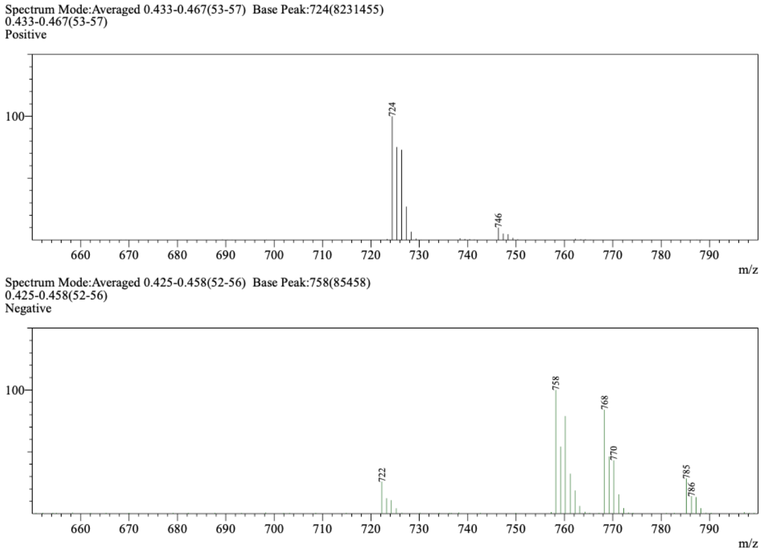

2-(Benzo[*d*][1,3]dioxol-5-yl)-2-(2-(3-(3-(*tert*-butylamino)-6-chloroimidazo[1,2-*a*]pyridin-2-yl)phenoxy)-*N*-(4-methoxyphenyl)acetamido)-*N*-cyclohexylacetamide (7h). Yield 58%, white solid. T_m = 147-118 ^oC. ^1H NMR (400 MHz, $CDCl_3$) δ 8.27 (s, 1H), 7.45 (dd, J = 14.5, 8.8 Hz, 2H), 7.37 – 7.20 (m, 3H), 7.15 – 7.01 (m, 1H), 6.69 (d, J = 77.8 Hz, 6H), 6.00 (d, J = 3.2 Hz, 1H), 5.88 (d, J = 3.9 Hz, 2H), 5.56 (d, J = 8.1 Hz, 1H), 4.37 (s, 2H), 3.74 (d, J = 3.4 Hz, 4H), 1.97 – 1.72 (m, 1H), 1.70 – 1.47 (m, 3H), 1.25 (s, 3H), 1.15 – 0.70 (m, 18H); ^{13}C NMR (100 MHz, $CDCl_3$) δ 168.7, 168.4, 159.5, 158.2, 147.7, 147.6, 139.9, 135.7, 131.4, 130.5, 129.4, 127.7, 125.6, 124.5, 124.3, 121.5, 119.9, 117.5, 114.9, 114.5, 114.3, 110.7, 108.1, 101.2, 77.3, 77.2, 76.9, 76.7, 67.0, 64.7, 56.4, 55.6, 48.9, 32.8, 30.2, 25.4, 24.8, 24.7.

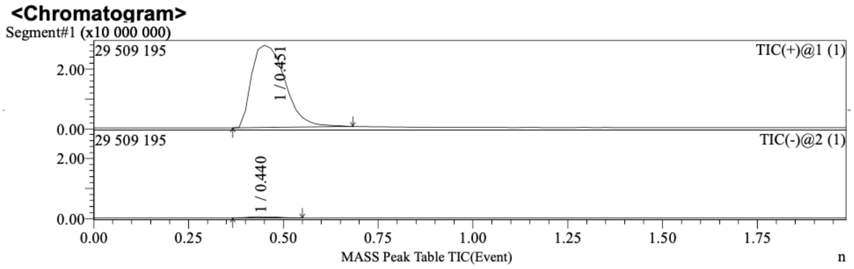

TIC(1-1)

Peak#	Ret. Time	m/z	Area	Compound Name	A/H	Event#	Area%	Height%
1	0.451	TIC	169053432		6.147	1-1	100.000	100.000
Total			169053432				100.000	100.000

TIC(1-2)

Peak#	Ret. Time	m/z	Area	Compound Name	A/H	Event#	Area%	Height%
1	0.440	TIC	1937320		5.341	1-2	100.000	100.000
Total			1937320				100.000	100.000

Spectrum Mode:Averaged 0.433-0.467(53-57) Base Peak:738(8466708)
0.433-0.467(53-57)
Positive

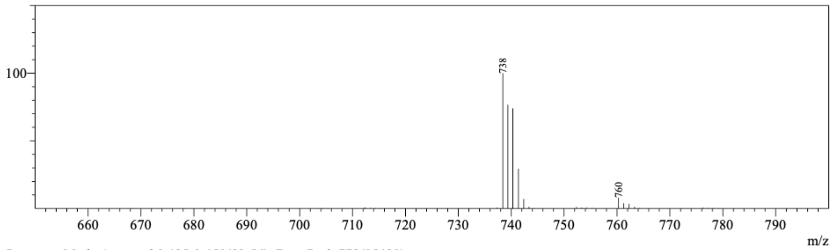

Spectrum Mode:Averaged 0.425-0.458(52-56) Base Peak:772(85420)
0.425-0.458(52-56)
Negative

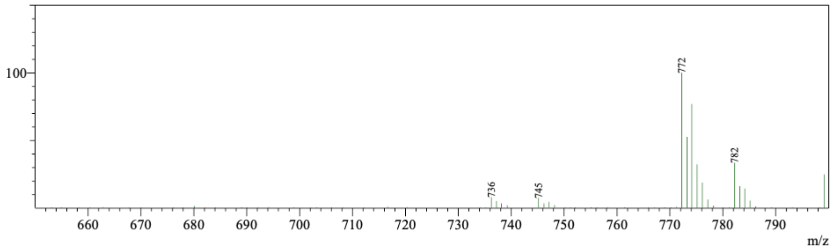

2-(3-(3-(*tert*-Butylamino)-6-chloroimidazo[1,2-*a*]pyridin-2-yl)phenoxy)-*N*-(2-(cyclohexylamino)-2-oxo-1-(4-(trifluoromethyl)phenyl)ethyl)-*N*-(4-methoxyphenyl)acetamide (7i). Yield 62%, white solid. T_m = 193-194 ^oC. ^1H NMR (400 MHz, DMSO-d_6) δ 8.53 (s, 1H), 8.04 (d, J = 7.6 Hz, 1H), 7.75 (d, J = 7.7 Hz, 1H), 7.63 (s, 1H), 7.50 (t, J = 8.2 Hz, 3H), 7.32 (s, 1H), 7.28 (dd, J = 13.5, 5.2 Hz, 2H), 7.22 (dd, J = 9.4, 2.0 Hz, 1H), 6.78 (s, 2H), 6.73 (dd, J = 8.0, 2.6 Hz, 1H), 6.14 (s, 1H), 4.64 (s, 1H), 4.45 (d, J = 15.2 Hz, 1H), 4.34 (d, J = 15.2 Hz, 1H), 3.68 (s, 3H), 3.55 (s, 1H), 2.50 (d, J = 4.6 Hz, 3H), 1.64 (dt, J = 32.1, 14.7 Hz, 5H), 1.50 (d, J = 12.2 Hz, 1H), 1.24 – 1.13 (m, 3H), 0.99 (s, 10H); ^{13}C NMR (100 MHz, DMSO-d_6) δ 167.5, 158.7, 158.5, 157.6, 140.0, 139.3, 138.6, 136.1, 131.7, 130.7, 130.1, 128.9, 125.1, 124.8,

124.7, 124.6, 121.8, 120.5, 118.5, 117.7, 113.8, 113.7, 113.3, 65.8, 63.1, 55.9, 55.2, 47.9, 40.1, 39.9, 39.9, 39.8, 39.7, 39.7, 39.6, 39.5, 39.4, 39.3, 39.2, 38.9, 32.1, 31.9, 29.9, 25.1, 24.5, 24.4.

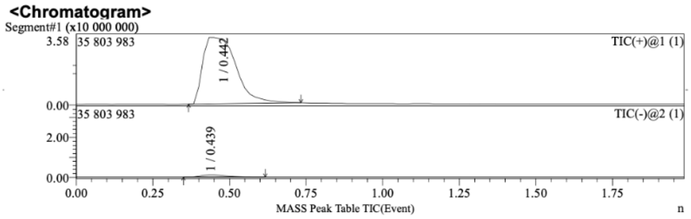

TIC(1-1)

Peak#	Ret. Time	m/z	Area	Compound Name	A/H	Event#	Area%	Height%
1	0.442	TIC	250615208		7.507	1-1	100.000	100.000
Total			250615208				100.000	100.000

TIC(1-2)

Peak#	Ret. Time	m/z	Area	Compound Name	A/H	Event#	Area%	Height%
1	0.439	TIC	5399378		5.257	1-2	100.000	100.000
Total			5399378				100.000	100.000

Spectrum Mode:Averaged 0.417-0.450(51-55) Base Peak:762(8535988)
0.417-0.450(51-55)
Positive

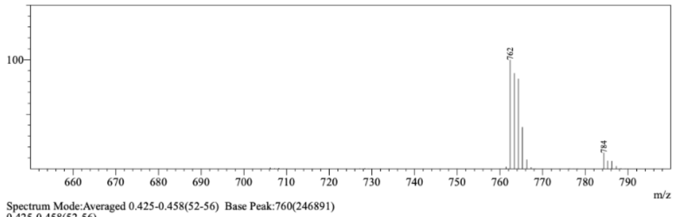

Spectrum Mode:Averaged 0.425-0.458(52-56) Base Peak:760(246891)
0.425-0.458(52-56)
Negative

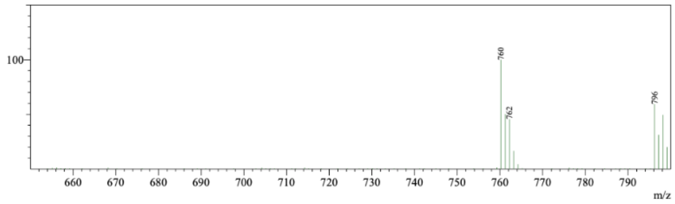

Ethyl (2-(2-(3-(3-(*tert*-butylamino)-6-chloroimidazo[1,2-*a*]pyridin-2-yl)phenoxy)-*N*-(4-methoxyphenyl)acetamido)-2-(4-chlorophenyl)acetyl)glycinate (7j). Yield 61%, yelowish solid. T_m = 98-99 °C. ^{1}H NMR (400 MHz, $CDCl_3$) δ 8.23 (s, 1H), 7.34 (dt, J = 66.6, 6.8 Hz, 6H), 7.10 (p, J = 7.8 Hz, 5H), 6.97 – 6.47 (m, 4H), 6.21 (s, 1H), 4.40 (s, 2H), 4.19 – 4.08 (m, 2H), 4.01 (d, J = 5.5 Hz, 2H), 3.74 (d, J = 3.0 Hz, 3H), 1.33 – 1.16 (m, 5H), 0.98 (d, J = 3.0 Hz, 9H), 0.85 (dd, J = 10.5, 6.6 Hz, 3H); ^{13}C NMR (100 MHz, DMSO-d_6) δ 169.5, 169.5, 167.5, 158.7, 158.5, 157.6, 139.3, 138.6, 136.1, 133.5, 132.5, 132.3, 131.7, 129.9, 128.9, 127.8, 124.8, 124.7, 121.8, 120.5, 118.4, 117.7, 113.9, 113.6, 113.5, 65.8, 62.6, 60.4, 55.9, 55.2, 40.9, 40.1, 39.9, 39.9, 39.8, 39.7, 39.6, 39.6, 39.5, 39.4, 39.3, 39.2, 38.9, 29.9, 13.9.

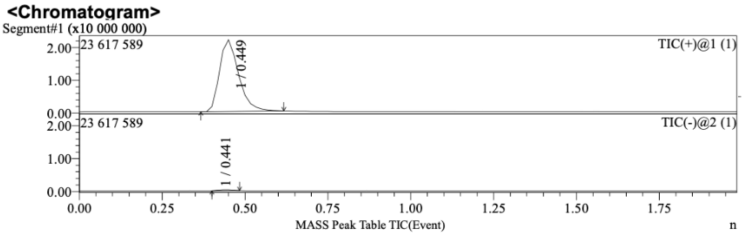

TIC(1-1)

Peak#	Ret. Time	m/z	Area	Compound Name	A/H	Event#	Area%	Height%
1	0.449	TIC	87689470		4.006	1-1	100.000	100.000
Total			87689470				100.000	100.000

TIC(1-2)

Peak#	Ret. Time	m/z	Area	Compound Name	A/H	Event#	Area%	Height%
1	0.441	TIC	708073		3.094	1-2	100.000	100.000
Total			708073				100.000	100.000

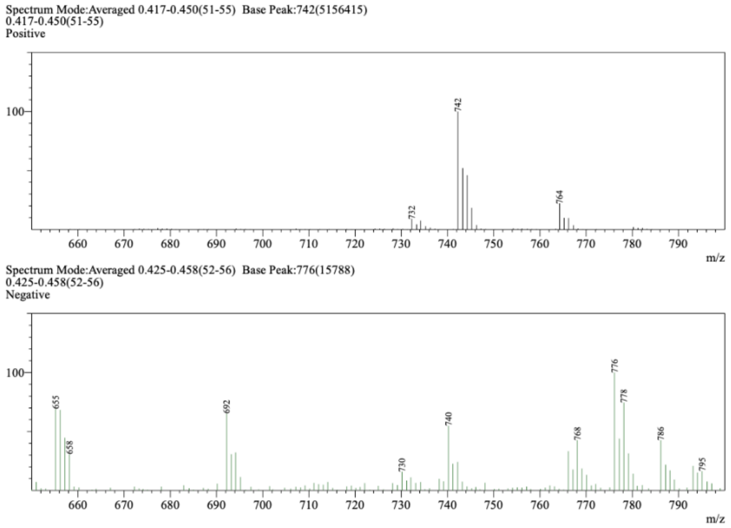

Ethyl (2-(2-(3-(3-(*tert*-butylamino)-6-chloroimidazo[1,2-*a*]pyridin-2-yl)phenoxy)-*N*-(4-methoxyphenyl)acetamido)-2-(4-methoxyphenyl)acetyl)glycinate (7k). Yield 40%, yelowish solid. T_m = 123-124 ^oC. ^1H NMR (400 MHz, $CDCl_3$) δ 8.26 (s, 1H), 7.44 (dd, J = 18.1, 8.5 Hz, 3H), 7.34 – 7.19 (m, 3H), 7.13 – 6.99 (m, 3H), 6.85 – 6.60 (m, 5H), 6.39 (s, 1H), 6.21 (s, 1H), 4.48 – 4.30 (m, 2H), 4.13 (d, J = 7.3 Hz, 2H), 4.03 (d, J = 4.9 Hz, 2H), 3.72 (s, 4H), 3.71 (s, 3H), 1.29 – 1.12 (m, 3H), 0.98 (d, J = 1.7 Hz, 11H); ^{13}C NMR (100 MHz, DMSO-d_6) δ 170.6, 170.1, 167.9, 159.2, 159.0, 158.2, 139.8, 139.1, 136.7,` 132.2, 130.7, 129.4, 126.8, 125.4, 125.2, 122.3, 120.9, 120.9, 118.9, 118.2, 114.3, 114.1, 114.0, 113.7, 66.3, 63.3, 60.9, 56.5, 55.6, 55.5, 41.5, 40.6, 40.4, 40.2, 40.0, 39.8, 39.6, 39.4, 30.5, 14.5.

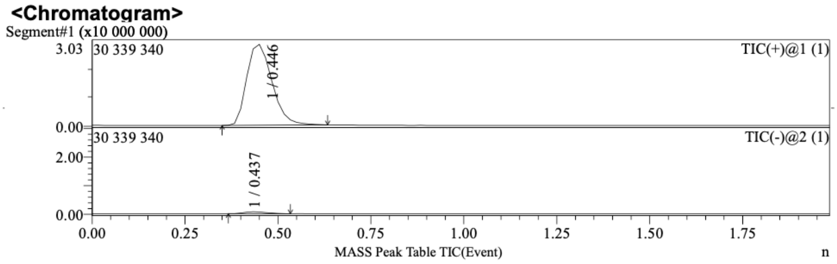

TIC(1-1)

Peak#	Ret. Time	m/z	Area	Compound Name	A/H	Event#	Area%	Height%
1	0.446	TIC	134204318		4.729	1-1	100.000	100.000
Total			134204318				100.000	100.000

TIC(1-2)

Peak#	Ret. Time	m/z	Area	Compound Name	A/H	Event#	Area%	Height%
1	0.437	TIC	2825455		4.299	1-2	100.000	100.000
Total			2825455				100.000	100.000

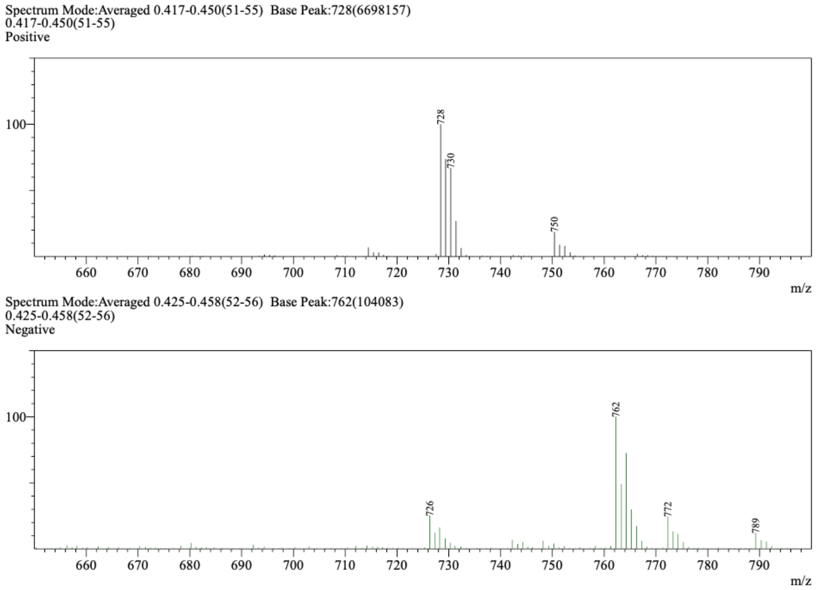

Ethyl (2-(benzo[*d*][1,3]dioxol-5-yl)-2-(2-(3-(3-(*tert*-butylamino)-6-chloroimidazo[1,2-*a*]pyridin-2-yl)phenoxy)-*N*-(4-methoxyphenyl)acetamido)acetyl)glycinate (7l). Yield 28%, yelowish solid. T_m = 108-110°C. ^{1}H NMR (400 MHz, DMSO-d_6) δ 8.47 (d, J = 15.7 Hz, 3H), 7.71 (d, J = 7.8 Hz, 1H), 7.58 (s, 1H), 7.50 – 7.41 (m, 1H), 7.36 (d, J = 7.8 Hz, 1H), 7.22 (q, J = 9.4 Hz, 3H), 6.97 (dd, J = 30.7, 8.3 Hz, 2H), 6.87 – 6.52 (m, 6H), 5.87 (s, 2H), 4.59 (s, 1H), 4.44 – 4.18 (m, 2H), 4.03 (q, J = 7.3 Hz, 2H), 3.70 (d, J = 28.6 Hz, 5H), 1.13 (t, J = 7.1 Hz, 3H), 0.95 (s, 9H); ^{13}C NMR (100 MHz, DMSO-d_6) δ 169.9, 169.5, 158.5, 157.7, 146.6, 139.3, 138.6, 136.1, 131.7, 128.9, 127.9, 124.8, 124.7, 124.4, 121.8, 120.4, 118.4, 117.7, 113.8, 113.6, 113.5, 110.7, 107.6, 100.9, 65.8, 63.0, 60.4, 55.9, 55.1, 41.0, 39.8, 39.6, 39.5, 39.3, 39.1, 38.9, 30.9, 29.9, 22.0, 13.9, 13.9.

<Chromatogram>

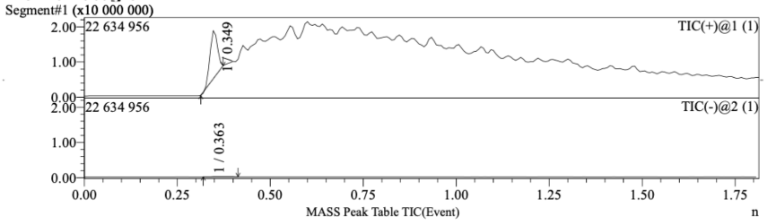

MASS Peak Table TIC(Event)

TIC(1-1)

Peak#	Ret. Time	m/z	Area	Compound Name	A/H	Event#	Area%	Height%
1	0.349	TIC	20759471		1.553	1-1	100.000	100.000
Total			20759471				100.000	100.000

TIC(1-2)

Peak#	Ret. Time	m/z	Area	Compound Name	A/H	Event#	Area%	Height%
1	0.363	TIC	184241		2.543	1-2	100.000	100.000
Total			184241				100.000	100.000

Spectrum Mode:Averaged 0.340-0.353(103-107) Base Peak:742(4615667)
0.340-0.353(103-107)
Positive

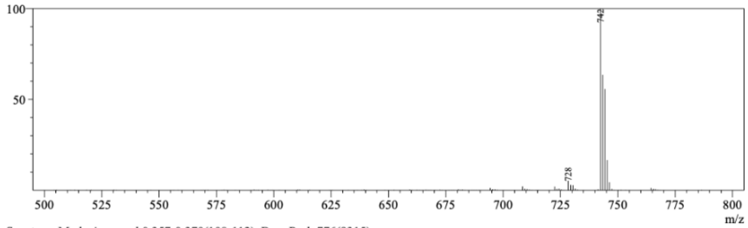

Spectrum Mode:Averaged 0.357-0.370(108-112) Base Peak:776(9315)
0.357-0.370(108-112)
Negative

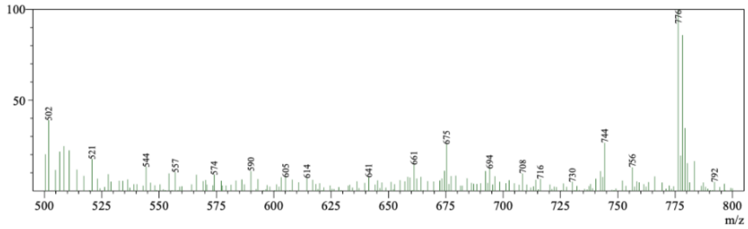

2-(3-(3-(*tert*-Butylamino)-6-chloroimidazo[1,2-*a*]pyridin-2-yl)phenoxy)-*N*-(1-(4-chlorophenyl)-2-((2-nitrobenzyl)amino)-2-oxoethyl)-*N*-(4-methoxyphenyl)acetamide (7m). Yield 53%, yelowish solid. T_m = 207-208 ^oC. ^1H NMR (400 MHz, DMSO-d_6) δ 8.75 (d, J = 6.4 Hz, 1H), 8.48 (s, 1H), 7.96 (d, J = 8.0 Hz, 1H), 7.72 (d, J = 7.7 Hz, 1H), 7.60 (s, 1H), 7.47 (tq, J = 14.1, 7.3 Hz, 5H), 7.22 (dd, J = 21.7, 8.3 Hz, 4H), 7.08 (d, J = 8.1 Hz, 2H), 6.89 – 6.64 (m, 3H), 6.10 (s, 1H), 4.58 (dd, J = 12.2, 5.6 Hz, 3H), 4.47 – 4.27 (m, 2H), 3.66 (s, 3H), 0.96 (s, 9H); ^{13}C NMR (100 MHz, DMSO-d_6) δ 170.42, 168.21, 159.24, 158.16, 148.14, 139.82, 139.13, 136.66, 134.51, 134.08, 133.89, 133.19, 132.56, 132.18, 130.62, 130.45, 129.70, 129.44, 128.6, 128.5, 125.3, 124.9, 122.3, 121.0, 118.9, 118.2, 114.9, 114.5, 114.2, 114.1, 66.4, 63.9, 56.5, 55.7, 40.6, 40.4, 40.2, 40.0, 39.8, 39.6, 39.4, 30.5.

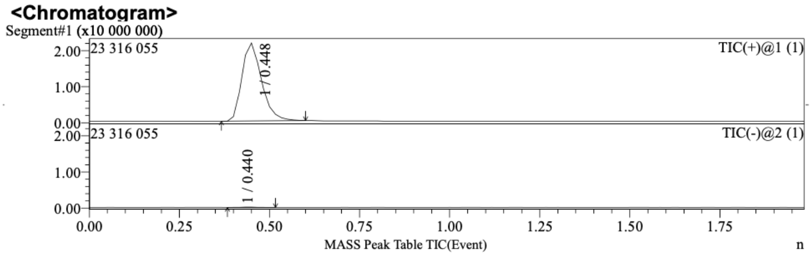

TIC(1-1)

Peak#	Ret. Time	m/z	Area	Compound Name	A/H	Event#	Area%	Height%
1	0.448	TIC	81476277		3.774	1-1	100.000	100.000
Total			81476277				100.000	100.000

TIC(1-2)

Peak#	Ret. Time	m/z	Area	Compound Name	A/H	Event#	Area%	Height%
1	0.440	TIC	410544		3.437	1-2	100.000	100.000
Total			410544				100.000	100.000

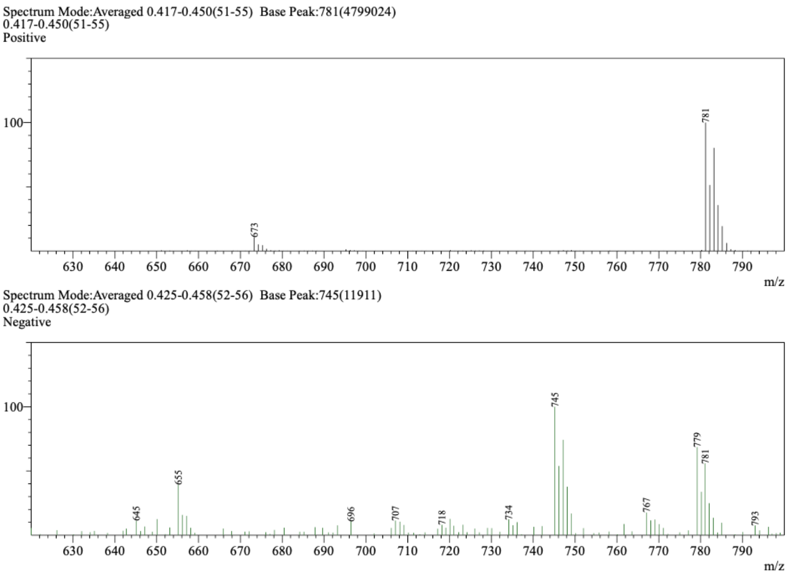

2-(3-(3-(*tert*-Butylamino)-6-chloroimidazo[1,2-*a*]pyridin-2-yl)phenoxy)-*N*-(4-methoxyphenyl)-*N*-(1-(4-methoxyphenyl)-2-((2-nitrobenzyl)amino)-2-oxoethyl)acetamide (7n). Yield 43%, yelowish solid. T_m = 234-235 °C. ^{1}H NMR (400 MHz, $CDCl_3$) δ 8.19 (s, 1H), 7.85 (d, J = 8.1 Hz, 1H), 7.53 (d, J = 7.7 Hz, 1H), 7.39 (dt, J = 13.8, 7.8 Hz, 3H), 7.29 (s, 1H), 7.27 – 7.19 (m, 2H), 7.02 (dd, J = 24.1, 8.8 Hz, 3H), 6.82 – 6.58 (m, 7H), 6.02 (s, 1H), 4.66 (qd, J = 16.0, 6.3 Hz, 2H), 4.37 (d, J = 3.0 Hz, 2H), 3.71 (s, 4H), 3.70 (s, 3H), 0.99 (s, 9H); ^{13}C NMR (100 MHz, $CDCl_3$) δ 170.4, 168.9, 159.7, 159.4, 158.2, 147.9, 140.2, 139.9, 135.8, 133.9, 133.5, 131.7, 131.3, 130.6, 129.3, 128.1, 125.5, 124.6, 124.2, 121.5, 121.4, 119.9, 117.4, 115.0, 114.3, 114.3, 113.9, 77.3, 77.0, 76.7, 67.1, 65.2, 56.4, 55.3, 55.2, 41.4, 30.3.

<Chromatogram>

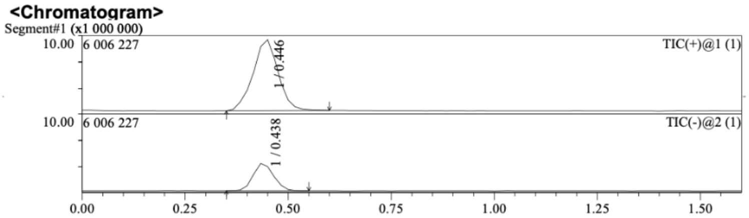

MASS Peak Table TIC(Event)

TIC(1-1)

Peak#	Ret. Time	m/z	Area	Compound Name	A/H	Event#	Area%	Height%
1	0.446	TIC	22917974		4.250	1-1	100.000	100.000
Total			22917974				100.000	100.000

TIC(1-2)

Peak#	Ret. Time	m/z	Area	Compound Name	A/H	Event#	Area%	Height%
1	0.438	TIC	7191122		3.480	1-2	100.000	100.000
Total			7191122				100.000	100.000

Spectrum Mode:Averaged 0.417-0.450(51-55) Base Peak:747(900294)
0.417-0.450(51-55)
Positive

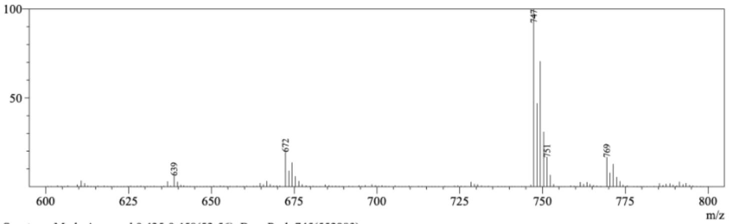

Spectrum Mode:Averaged 0.425-0.458(52-56) Base Peak:745(552983)
0.425-0.458(52-56)
Negative

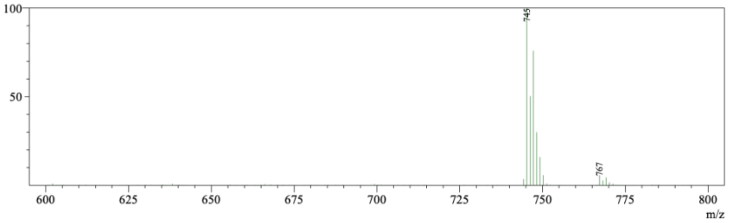

***N*-(*tert*-Butyl)-2-(2-(3-(3-(*tert*-butylamino)-6-chloroimidazo[1,2-*a*]pyridin-2-yl)phenoxy)-*N*-phenylacetamido)-2-(4-chlorophenyl)acetamide (7o).** Yield 63%, yelowish solid. T_m = 134-135 ^oC. ^1H NMR (400 MHz, $CDCl_3$) δ 8.25 (d, J = 2.0 Hz, 1H), 7.47 – 7.40 (m, 3H), 7.34 – 7.22 (m, 7H), 7.15 – 7.04 (m, 6H), 6.76 (d, J = 8.4 Hz, 1H), 5.93 (s, 1H), 5.65 (s, 1H), 4.40 (s, 2H), 3.45 (s, 3H), 3.27 (s, 1H), 1.24 (s, 9H), 1.00 (s, 9H); ^{13}C NMR (100 MHz, DMSO-d_6) δ 168.9, 167.4, 158.1, 139.8, 139.2, 138.3, 136.7, 134.7, 132.8, 132.3, 131.3, 129.4, 129.2, 128.3, 125.3, 125.2, 122.3, 120.9, 118.9, 118.2, 113.9, 66.4, 63.7, 56.5, 50.9, 40.6, 40.4, 40.2, 40.0, 39.8, 39.6, 39.4, 30.5, 28.8.

<Chromatogram>

Segment#1 (x10 000 000)

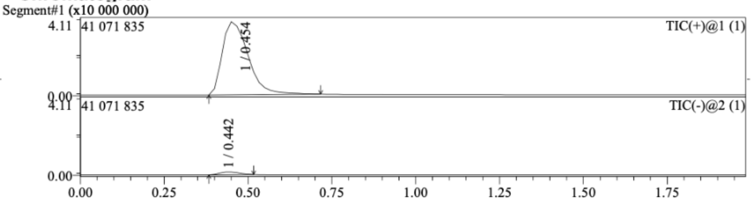

MASS Peak Table TIC(Event)

TIC(1-1)

Peak#	Ret. Time	m/z	Area	Compound Name	A/H	Event#	Area%	Height%
1	0.454	TIC	205660424		5.352	1-1	100.000	100.000
Total			205660424				100.000	100.000

TIC(1-2)

Peak#	Ret. Time	m/z	Area	Compound Name	A/H	Event#	Area%	Height%
1	0.442	TIC	5706296		4.145	1-2	100.000	100.000
Total			5706296				100.000	100.000

Spectrum Mode:Averaged 0.433-0.467(53-57) Base Peak:672(8458506)
0.433-0.467(53-57)
Positive

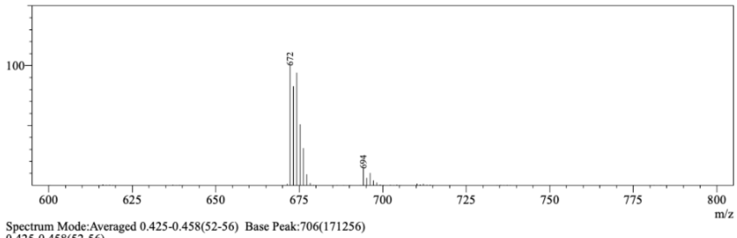

Spectrum Mode:Averaged 0.425-0.458(52-56) Base Peak:706(171256)
0.425-0.458(52-56)
Negative

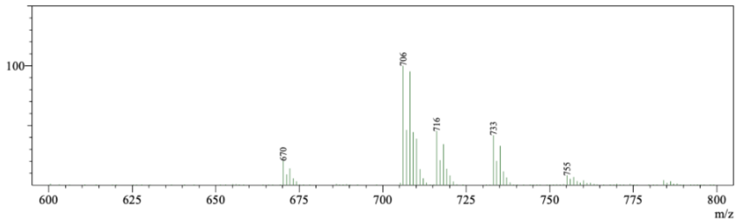

***N*-(*tert*-Butyl)-2-(2-(3-(3-(*tert*-butylamino)-6-chloroimidazo[1,2-*a*]pyridin-2-yl)phenoxy)-*N*-(5-methylisoxazol-3-yl)acetamido)-2-(4-chlorophenyl)acetamide (7p).** Yield 30%, yelowish solid. T_m = 218-220 ºC. ^{1}H NMR (400 MHz, $CDCl_3$) δ 8.25 (s, 1H), 7.53 – 7.41 (m, 3H), 7.37 – 7.22 (m, 4H), 7.22 – 7.12 (m, 5H), 7.08 (d, J = 9.2 Hz, 1H), 6.81 (d, J = 8.0 Hz, 1H), 6.05 (dd, J = 18.3, 5.6 Hz, 2H), 5.90 (s, 1H), 4.82 (d, J = 15.3 Hz, 1H), 4.59 (d, J = 15.5 Hz, 1H), 3.30 (s, 1H), 2.36 – 2.23 (m, 4H), 1.25 (d, J = 2.5 Hz, 9H), 1.04 – 0.92 (m, 9H); ^{13}C NMR (100 MHz, DMSO-d_6) δ 171.2, 167.4, 167.1, 159.6, 157.4, 139.3, 138.7, 136.2, 133.7, 132.7, 131.2, 128.9, 128.1, 124.8, 124.7, 121.8, 120.7, 118.4, 117.7, 113.6, 113.5, 102.5, 65.8, 61.5, 55.9, 50.5, 40.1, 39.9, 39.9, 39.8, 39.7, 39.7, 39.6, 39.5, 39.4, 39.3, 39.2, 38.9, 29.9, 28.2, 12.2.

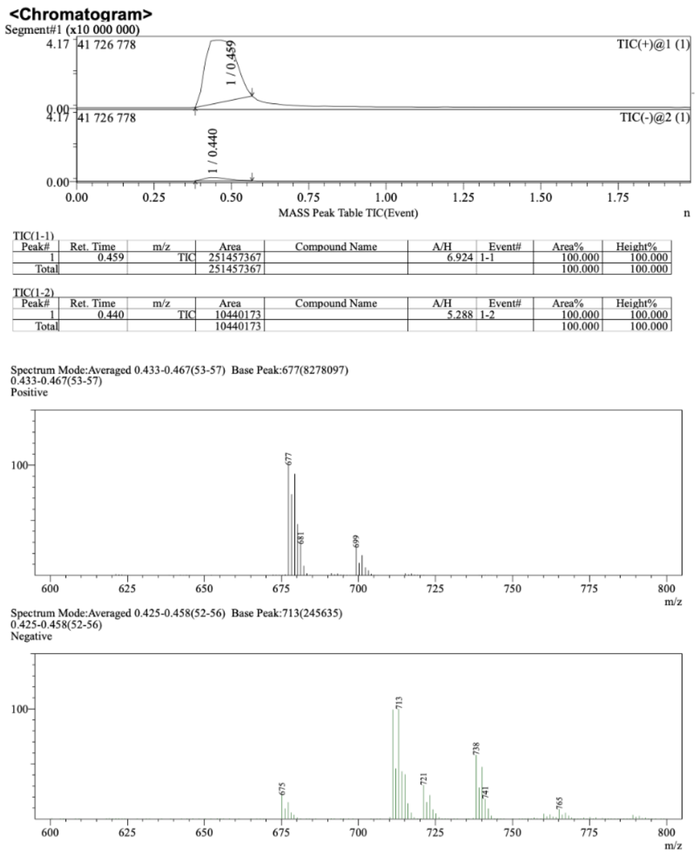

***N*-(*tert*-Butyl)-2-(2-(3-(3-(*tert*-butylamino)-6-chloroimidazo[1,2-*a*]pyridin-2-yl)phenoxy)-*N*-(5-methylisoxazol-3-yl)acetamido)-2-(4-methoxyphenyl)acetamide (7q).** Yield 37%, yelowish solid. T_m = 141-147 °C. ^{1}H NMR (400 MHz, DMSO-d_6) δ 8.49 (s, 1H), 7.82 (s, 1H), 7.69 (d, J = 7.3 Hz, 1H), 7.59 (s, 1H), 7.49 (d, J = 9.5 Hz, 1H), 7.33 – 7.14 (m, 2H), 7.05 (d, J = 8.3 Hz, 2H), 6.73 (dd, J = 19.3, 8.8 Hz, 3H), 6.25 (s, 1H), 6.03 (s, 1H), 4.73 (d, J = 16.1 Hz, 1H), 4.60 (s, 1H), 4.48 (d, J = 15.6 Hz, 1H), 3.65 (s, 3H), 2.25 (s, 3H), 1.16 (s, 9H), 0.96 (s, 9H); ^{13}C NMR (100 MHz, DMSO-d_6) δ 170.8, 167.9, 167.3, 159.8, 158.8, 158.5, 157.4, 139.3, 138.8, 136.2, 130.8, 128.9, 126.2, 124.8, 124.7, 121.8, 120.7, 118.4, 117.7, 113.6, 113.5, 113.5, 102.5, 65.8, 61.8, 55.9, 54.9, 50.4, 40.1, 39.9, 39.9, 39.8, 39.7, 39.7, 39.6, 39.5, 39.4, 39.3, 39.2, 38.9, 29.9, 28.2, 12.2.

<Chromatogram>

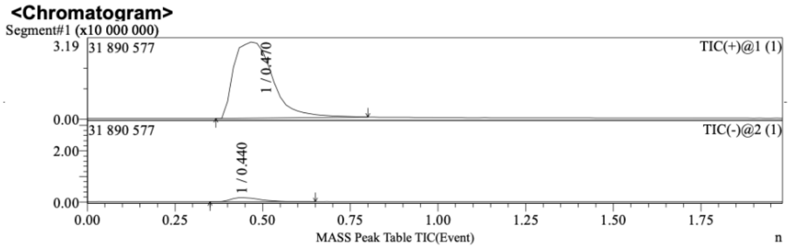

MASS Peak Table TIC(Event)

TIC(1-1)

Peak#	Ret. Time	m/z	Area	Compound Name	A/H	Event#	Area%	Height%
1	0.470	TIC	230261753		7.735	1-1	100.000	100.000
Total			230261753				100.000	100.000

TIC(1-2)

Peak#	Ret. Time	m/z	Area	Compound Name	A/H	Event#	Area%	Height%
1	0.440	TIC	8775205		5.692	1-2	100.000	100.000
Total			8775205				100.000	100.000

Spectrum Mode:Averaged 0.450-0.483(55-59) Base Peak:673(8661393)
0.450-0.483(55-59)
Positive

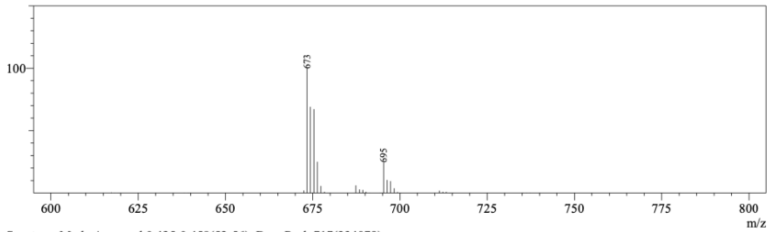

Spectrum Mode:Averaged 0.425-0.458(52-56) Base Peak:717(234079)
0.425-0.458(52-56)
Negative

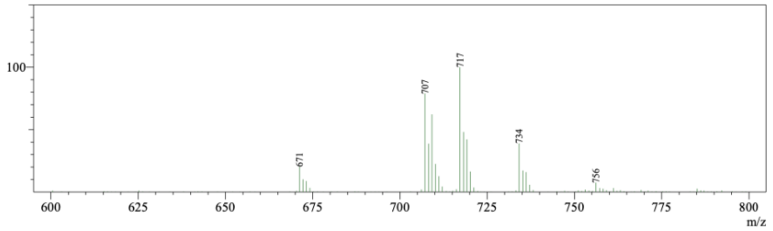

***N*-(*tert*-Butyl)-2-(2-(3-(6-chloro-3-(cyclohexylamino)imidazo[1,2-*a*]pyridin-2-yl)phenoxy)-*N*-(4-methoxyphenyl)acetamido)-2-(4-chlorophenyl)acetamide (7r).** Yield 54%, white solid. T_m = 187-188 ^oC. ^1H NMR (400 MHz, $CDCl_3$) δ 8.16 (s, 1H), 7.66 – 7.41 (m, 4H), 7.29 (t, J = 8.0 Hz, 2H), 7.11 (q, J = 8.1 Hz, 6H), 6.79 (d, J = 8.7 Hz, 4H), 5.94 (s, 1H), 5.67 (s, 1H), 4.43 (s, 2H), 3.75 (s, 4H), 1.65 (d, J = 73.9 Hz, 6H), 1.26 (s, 18H); ^{13}C NMR (100 MHz, DMSO-d_6) δ 168.5, 167.2, 158.6, 157.9, 138.6, 135.5, 135.1, 134.4, 132.2, 131.8, 130.2, 129.2, 127.8, 126.7, 124.5, 120.9, 119.1, 118.6, 117.7, 113.8, 113.4, 112.2, 65.7, 63.1, 56.5, 55.1, 50.3, 39.9, 39.9, 39.8, 39.8, 39.7, 39.6, 39.5, 39.4, 39.3, 39.2, 38.9, 33.5, 33.4, 28.3, 25.3, 24.5.

<Chromatogram>

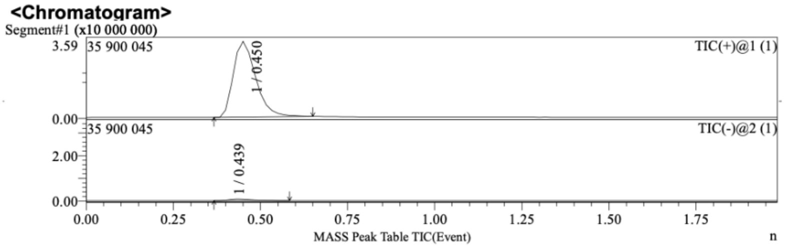

MASS Peak Table TIC(Event)

TIC(1-1)

Peak#	Ret. Time	m/z	Area	Compound Name	A/H	Event#	Area%	Height%
1	0.450	TIC	149687811		4.473	1-1	100.000	100.000
Total			149687811				100.000	100.000

TIC(1-2)

Peak#	Ret. Time	m/z	Area	Compound Name	A/H	Event#	Area%	Height%
1	0.439	TIC	2698073		4.188	1-2	100.000	100.000
Total			2698073				100.000	100.000

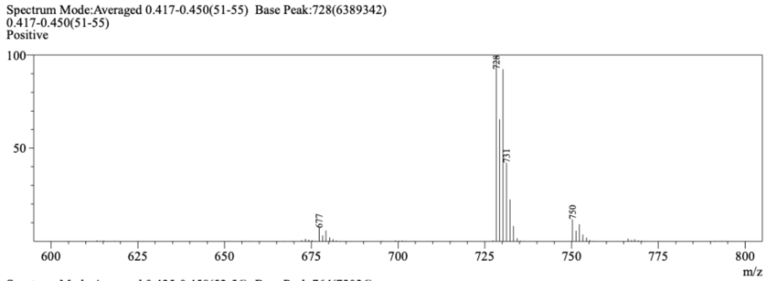

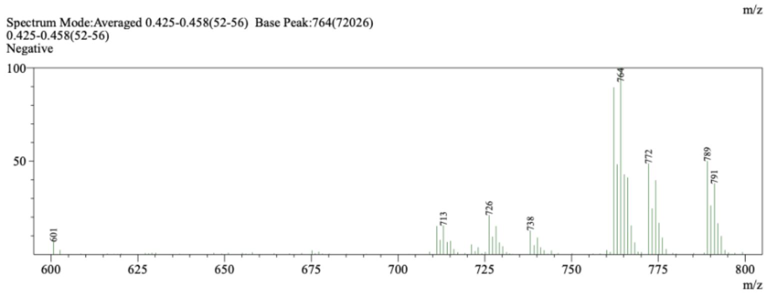

***N*-(*tert*-Butyl)-2-(2-(3-(6-chloro-3-(cyclohexylamino)imidazo[1,2-*a*]pyridin-2-yl)phenoxy)-*N*-(4-methoxyphenyl)acetamido)-2-(4-methoxyphenyl)acetamide (7s).** Yield 46%, white solid. T_m = 146-147 ^oC. ^1H NMR (400 MHz, $CDCl_3$) δ 8.11 (d, J = 6.9 Hz, 1H), 7.51 (t, J = 8.7 Hz, 2H), 7.44 (d, J = 3.3 Hz, 3H), 7.27 (dd, J = 16.8, 8.9 Hz, 2H), 7.03 (dd, J = 13.2, 9.2 Hz, 4H), 6.86 – 6.56 (m, 6H), 5.95 (s, 1H), 5.56 (s, 1H), 4.40 (d, J = 4.0 Hz, 2H), 3.73 (d, J = 7.0 Hz, 8H), 1.64 (d, J = 78.6 Hz, 7H), 1.28 – 1.03 (m, 20H); ^{13}C NMR (100 MHz, DMSO-d_6) δ 169.7, 167.6, 158.9, 158.5, 139.1, 135.9, 135.6, 132.3, 131.7, 130.9, 129.7, 127.6, 127.2, 124.9, 121.5, 119.6, 119.1, 118.2, 114.1, 113.9, 113.6, 112.8, 66.3, 63.7, 56.9, 55.4, 55.4, 50.7, 40.5, 40.4, 40.3, 40.1, 40.0, 39.9, 39.7, 39.6, 34.0, 33.9, 28.8, 25.8, 24.9.

<Chromatogram>

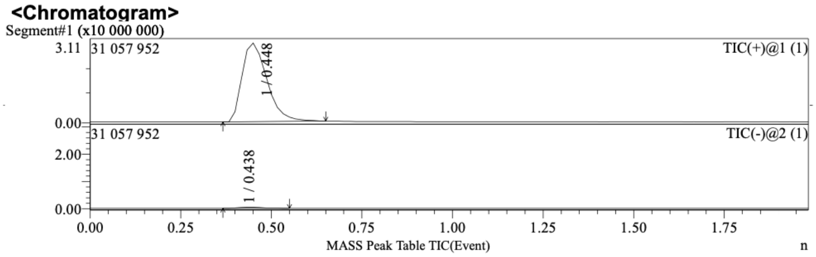

TIC(1-1)

Peak#	Ret. Time	m/z	Area	Compound Name	A/H	Event#	Area%	Height%
1	0.448	TIC	138319417		4.783	1-1	100.000	100.000
Total			138319417				100.000	100.000

TIC(1-2)

Peak#	Ret. Time	m/z	Area	Compound Name	A/H	Event#	Area%	Height%
1	0.438	TIC	1567095		4.250	1-2	100.000	100.000
Total			1567095				100.000	100.000

Spectrum Mode:Averaged 0.417-0.450(51-55) Base Peak:724(7263704)
0.417-0.450(51-55)
Positive

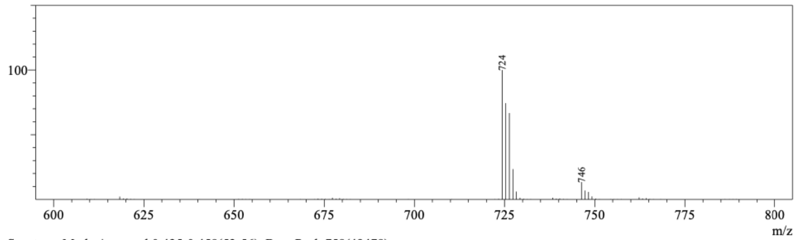

Spectrum Mode:Averaged 0.425-0.458(52-56) Base Peak:758(49478)
0.425-0.458(52-56)
Negative

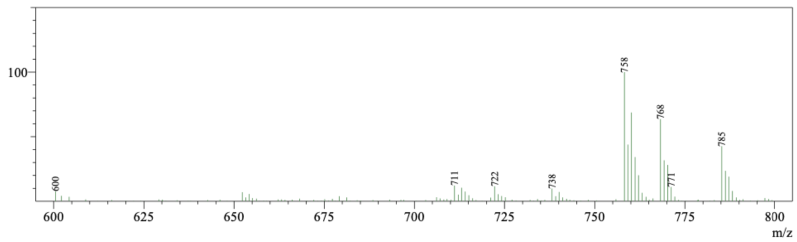

***N*-(*tert*-Butyl)-2-(2-(3-(6-(*tert*-butylamino)-1*H*-imidazo[1,2-*b*][1,2,4]triazol-5-yl)phenoxy)-*N*-(4-methoxyphenyl)acetamido)-2-(4-methoxyphenyl)acetamide (7t).** Yield 30%, yelowish solid. T_m = 200-201 ^oC. ^1H NMR (400 MHz, DMSO-d_6) δ 11.80 (s, 1H), 7.76 (d, J = 15.5 Hz, 2H), 7.54 – 7.35 (m, 2H), 7.23 (dd, J = 28.9, 8.1 Hz, 3H), 7.05 (d, J = 8.1 Hz, 2H), 6.67 (d, J = 8.5 Hz, 4H), 5.98 (s, 1H), 4.44 – 4.16 (m, 3H), 3.64 (s, 3H), 1.17 (s, 9H), 1.05 (s, 9H); ^{13}C NMR (100 MHz, DMSO-d_6) δ 168.6, 167.1, 158.5, 157.8, 153.1, 148.0, 134.2, 132.2, 131.9, 131.8, 130.1, 129.2, 127.8, 123.4, 120.7, 118.7, 113.8, 113.3, 112.3, 65.8, 63.2, 55.1, 54.4, 50.4, 48.6, 39.9, 39.9, 39.8, 39.7, 39.7, 39.6, 39.5, 39.4, 39.3, 39.1, 38.9, 30.1, 28.3.

<Chromatogram>

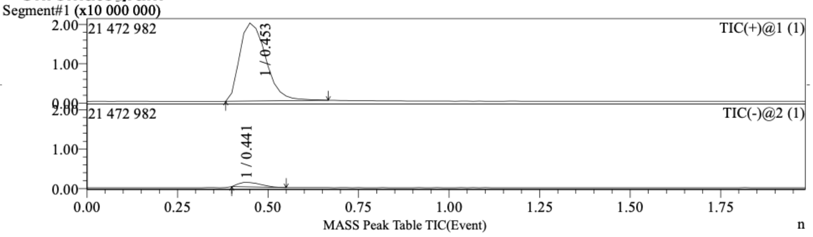

TIC(1-1)

Peak#	Ret. Time	m/z	Area	Compound Name	A/H	Event#	Area%	Height%
1	0.453	TIC	100254709		5.047	1-1	100.000	100.000
Total			100254709				100.000	100.000

TIC(1-2)

Peak#	Ret. Time	m/z	Area	Compound Name	A/H	Event#	Area%	Height%
1	0.441	TIC	4696636		4.169	1-2	100.000	100.000
Total			4696636				100.000	100.000

Spectrum Mode:Averaged 0.433-0.467(53-57) Base Peak:658(6467887)
0.433-0.467(53-57)
Positive

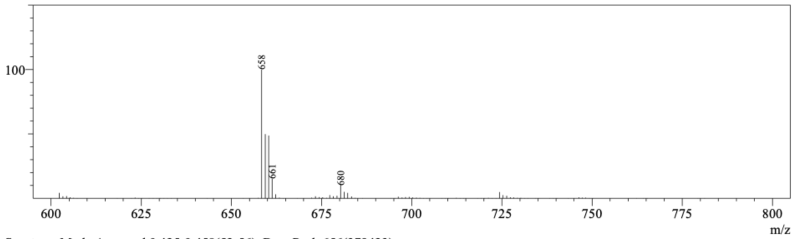

Spectrum Mode:Averaged 0.425-0.458(52-56) Base Peak:656(379423)
0.425-0.458(52-56)
Negative

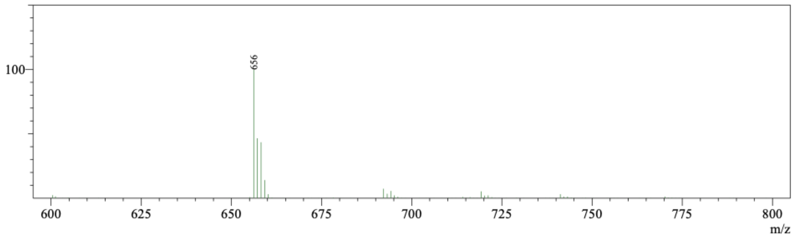

Synthesis of imidazo[1,2-a]containing heterocyclic acids 8a and 8b.

Amidine (2 mmol) and aldehyde (2 mmol) were added to a 10 mL round-bottom heat-resistant flask and dissolved in 4 mL of DMF. Then a 70% solution of $HClO_4$ in a catalytic amount (20 mol %) was added. Isocyanide was added to the resulting solution and left to stir for 24 hours. After 24 hours, the reaction mixture was poured onto ice, the precipitate obtained was filtered off and washed with cold methyl alcohol then dried in a vacuum and analyzed.

4-(3-(*tert*-Butylamino)-6-chloroimidazo[1,2-*a*]pyridin-2-yl)benzoic acid (8a). Yield 81%, yellowish solid. T_m = 275-276 ^oC. ^1H NMR (400 MHz, DMSO-d_6) δ 12.91 (s, 1H), 8.55 (d, J = 2.1 Hz, 1H), 8.31 (d, J = 8.1 Hz, 2H), 8.01 – 7.93 (m, 3H), 7.55 (d, J = 9.5 Hz, 1H), 7.24 (dd, J = 9.5, 2.1 Hz, 1H), 4.79 (s, 1H), 1.00 (s, 9H); ^{13}C NMR (100 MHz,

DMSO-d_6) δ 167.7, 162.7, 140.1, 139.7, 138.2, 129.6, 129.5, 127.9, 126.3, 125.7, 122.4, 119.2, 118.4, 56.7, 40.5, 40.4, 40.3, 40.1, 39.9, 39.8, 39.7, 39.6, 36.2, 31.2, 30.4.

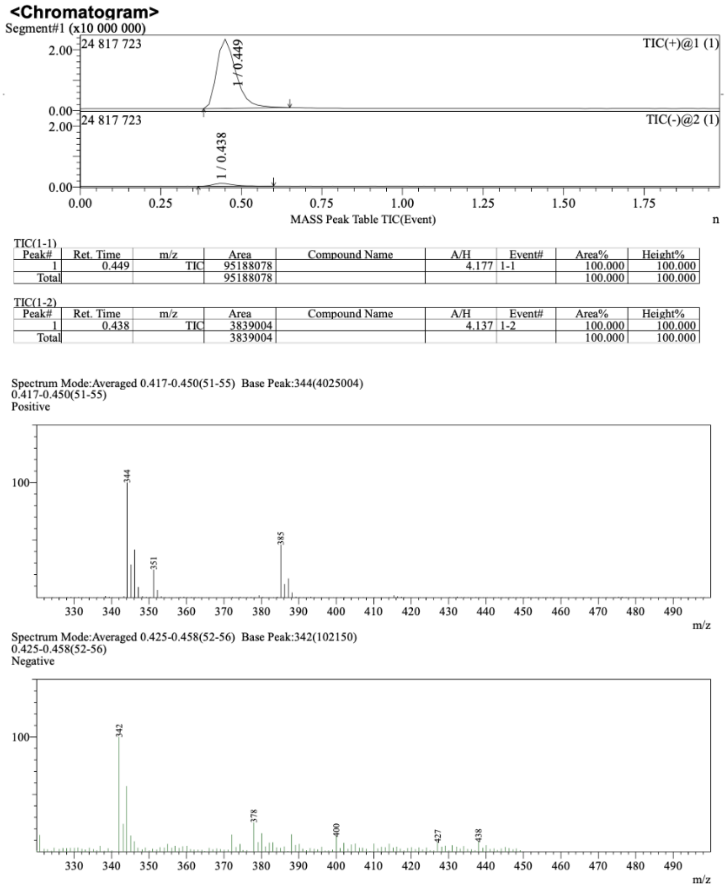

3-(3-(*tert*-Butylamino)-6-chloroimidazo[1,2-*a*]pyridin-2-yl)benzoic acid (8b). Yield 78%, yellowish solid. T_m = 279-280 ^{o}C. ^{1}H NMR (400 MHz, DMSO-d_6) δ 12.99 (s, 1H), 8.89 (s, 1H), 8.54 (s, 1H), 8.45 (d, J = 7.8 Hz, 1H), 7.86 (d, J = 7.7 Hz, 1H), 7.53 (q, J = 8.4 Hz, 2H), 7.23 (dd, J = 9.6, 2.1 Hz, 1H), 4.75 (s, 1H), 1.02 (s, 9H); ^{13}C NMR (100 MHz, DMSO-d_6) δ 167.41, 158.54, 139.52, 137.7, 135.1, 131.5, 130.6, 128.5, 128.2, 127.9, 125.2, 124.9, 121.9, 118.6, 117.78, 56.1, 39.9, 39.9, 39.8, 39.7, 39.6, 39.6, 39.5, 39.3, 39.1, 38.9, 29.9.

<Chromatogram>

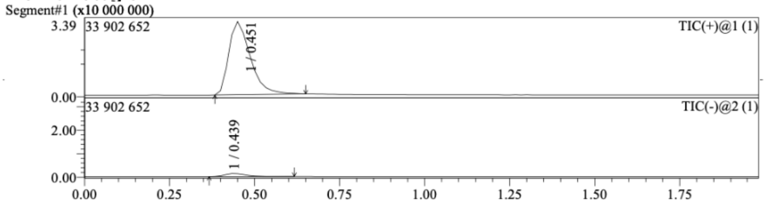

MASS Peak Table TIC(Event)

TIC(1-1)

Peak#	Ret. Time	m/z	Area	Compound Name	A/H	Event#	Area%	Height%
1	0.451	TIC	138050314		4.407	1-1	100.000	100.000
Total			138050314				100.000	100.000

TIC(1-2)

Peak#	Ret. Time	m/z	Area	Compound Name	A/H	Event#	Area%	Height%
1	0.439	TIC	5455836		4.340	1-2	100.000	100.000
Total			5455836				100.000	100.000

Spectrum Mode:Averaged 0.433-0.467(53-57) Base Peak:344(6048902)
0.433-0.467(53-57)
Positive

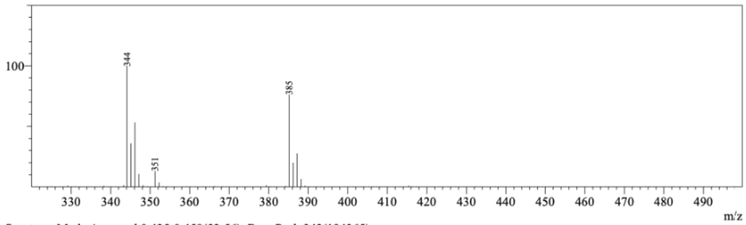

Spectrum Mode:Averaged 0.425-0.458(52-56) Base Peak:342(194365)
0.425-0.458(52-56)
Negative

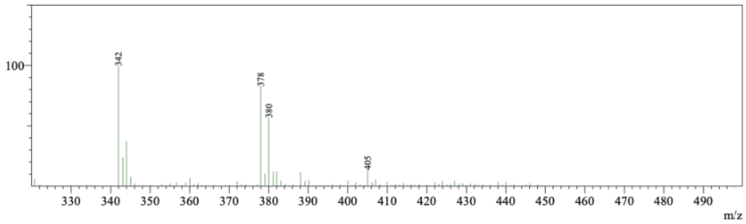

Procedure for the synthesis of peptidomimetics 9a and 9b.

A mixture of aminopyridine (1.0 mmol) in MeOH/DCM (2:3, 5.0 mL) and aldehyde (1.0 mmol) containing $Sc(OTf)_3$ (5 mol %) in a screw cap vial was stirred for 45 min at room temperature followed by the addition of the desired isocyanide (1.05 mmol), and the mixture was stirred for another 8 h. Without workup, to the resulting iminopyridine product was added the desired aldehyde (1.0 mmol), amine (1.0 mmol), and isocyanide (1.0 mmol), and the mixture was then stirred at room temperature for 12 h. The resulting reaction mixture was concentrated under reduced pressure and analyze with ^{1}H NMR.

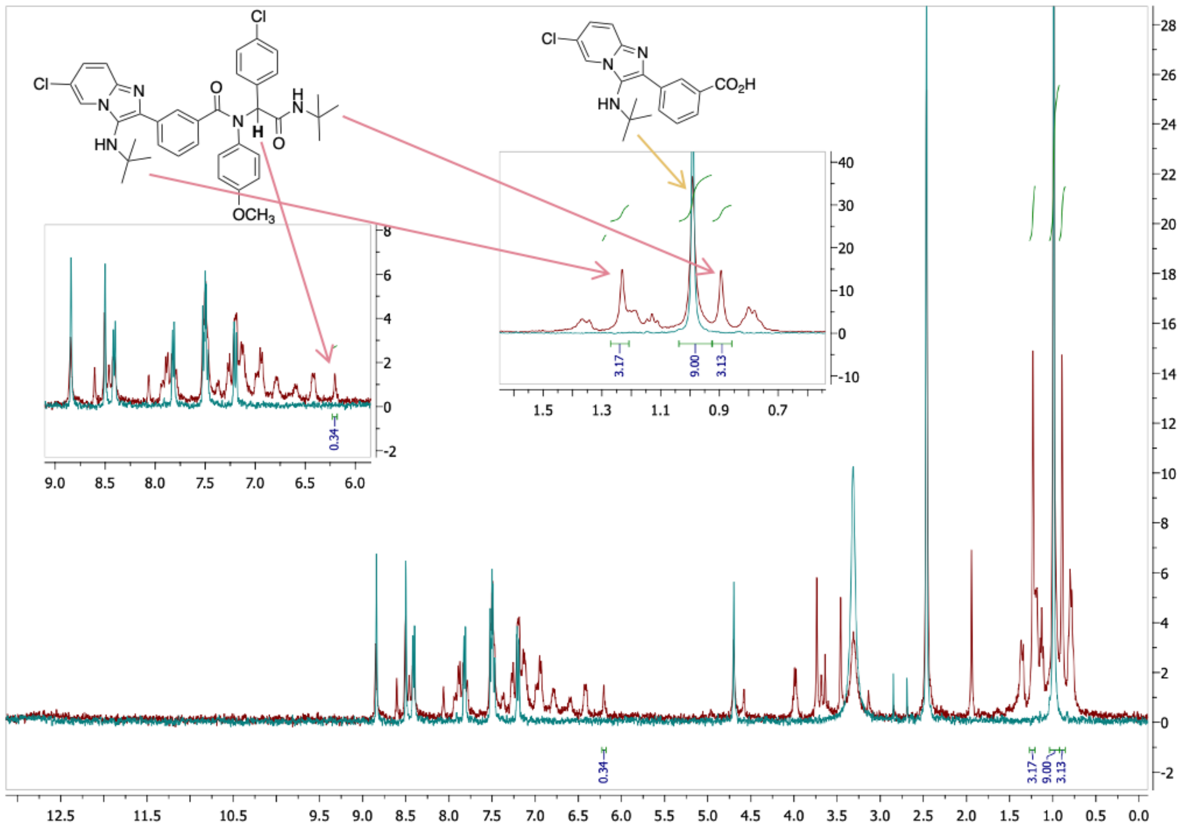

Fig.1: NMR spectra of the reaction mixture **9b** and pure compound **8b** (Red - **9b**, Green - **8b**). Integrated signals show ratio between starting acid **8b** and product **9b**.

3. ^{1}H and ^{13}C NMR spectra

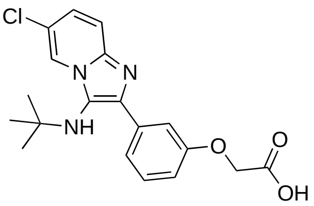

4a

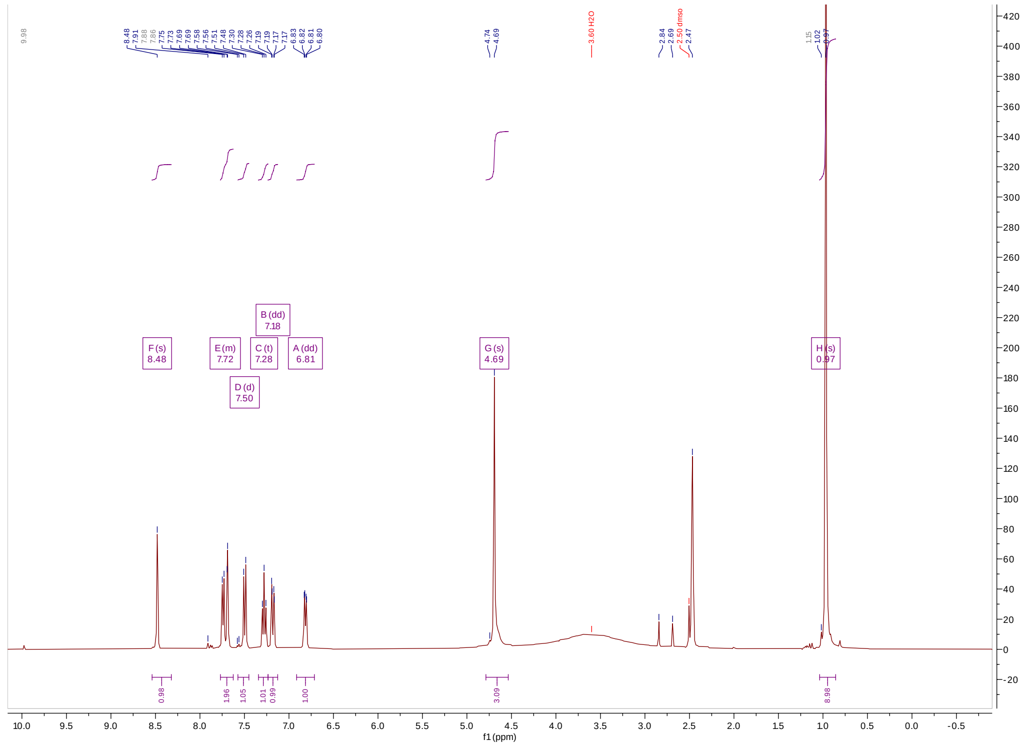

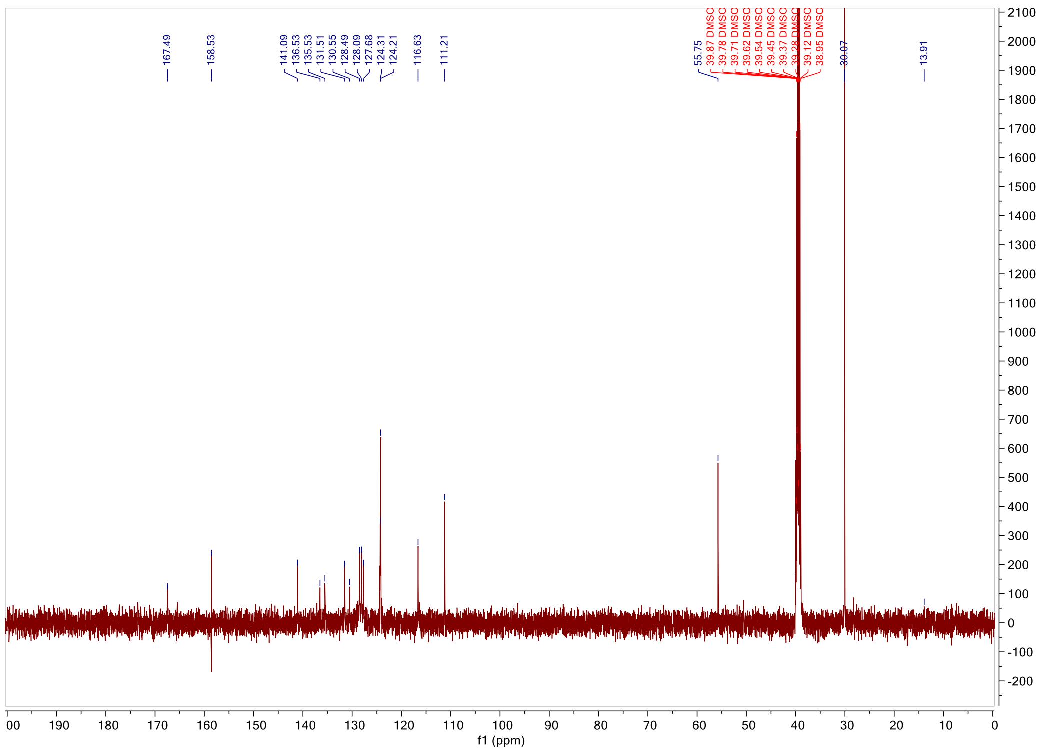

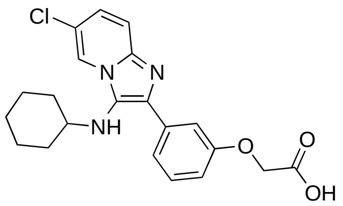

4b

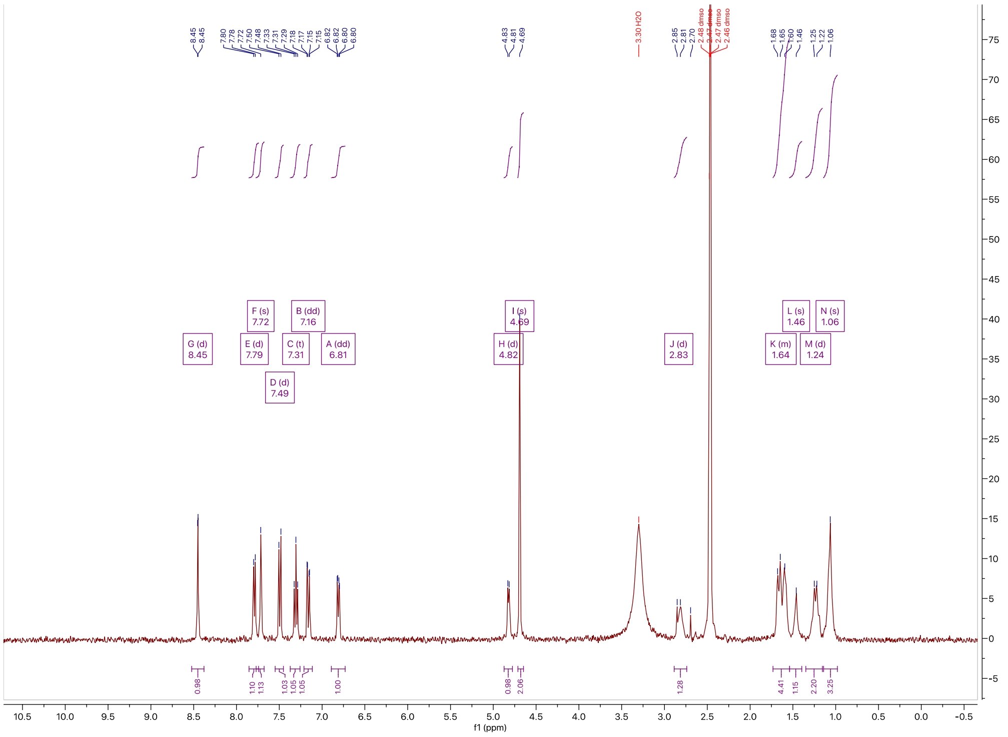

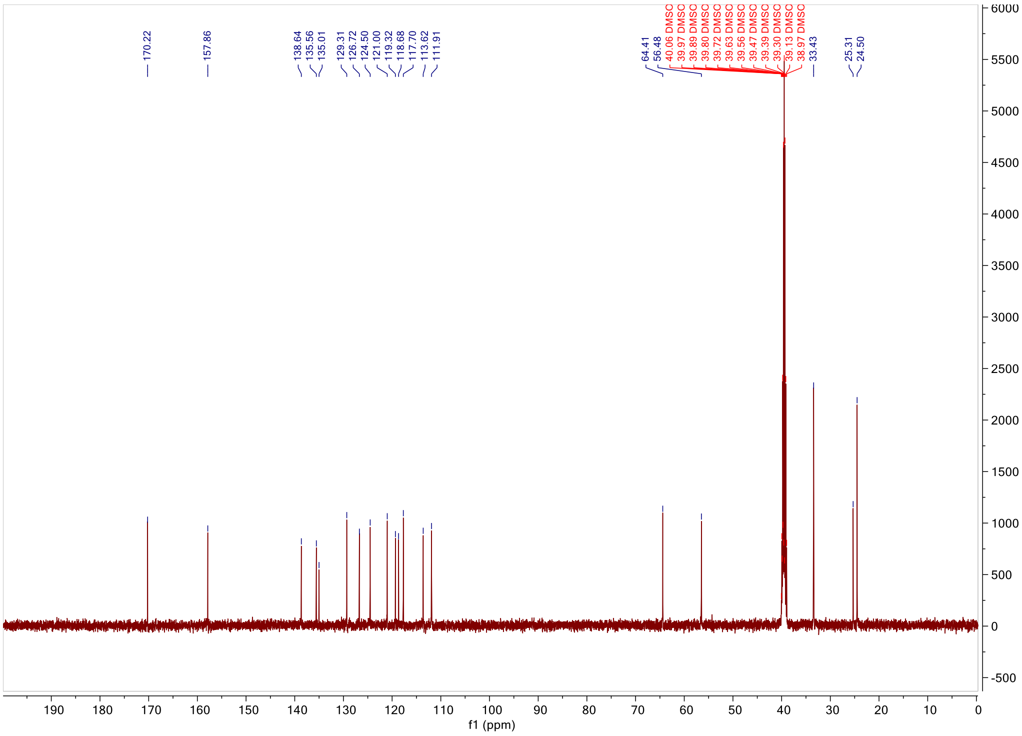

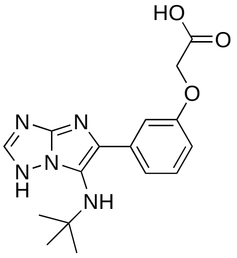
HO
O
O
N
N
N
N
H
NH

4c

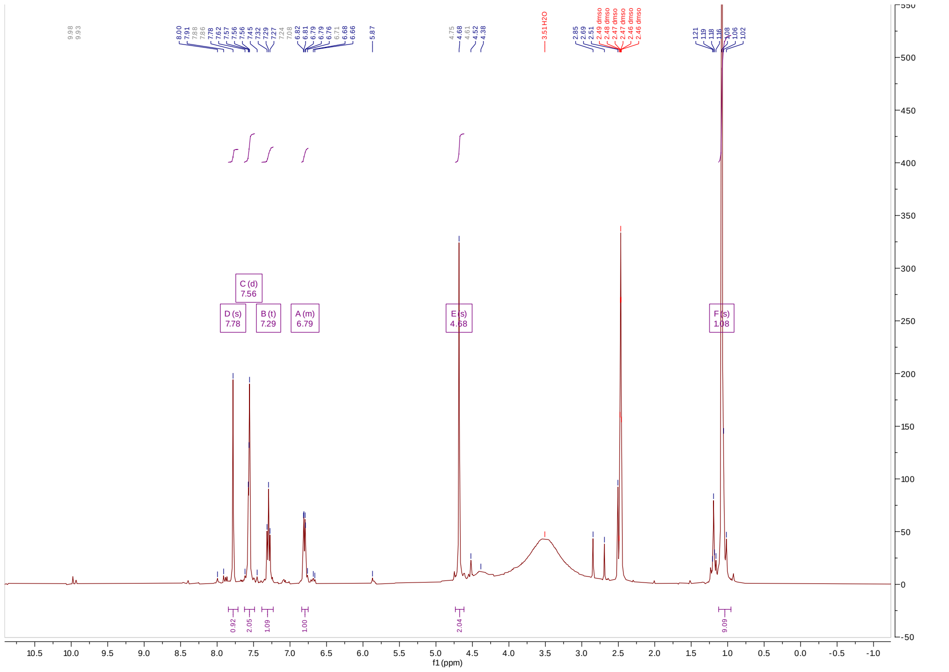
D (s)
7.78
C (d)
7.56
B (t)
7.29
A (m)
6.79
E (s)
4.68
F (s)
1.08
3.51 H2O
f1 (ppm)

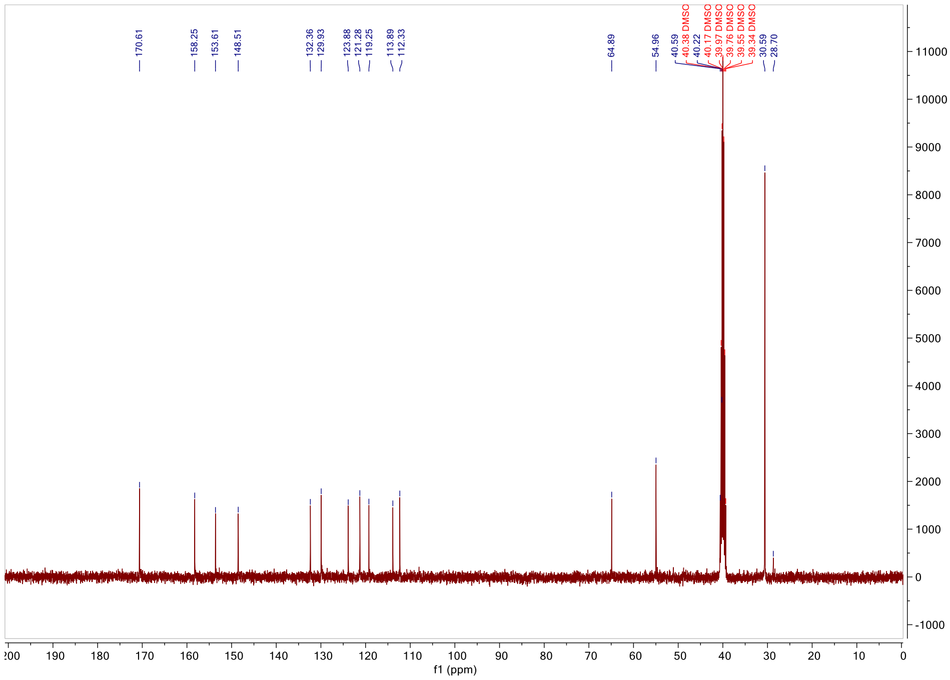
170.61
158.25
153.61
148.51
132.36
129.93
123.88
121.28
119.25
113.89
112.33
64.89
54.96
40.59
40.38 DMSO
40.22
40.17 DMSO
39.97 DMSO
39.76 DMSO
39.55 DMSO
39.34 DMSO
30.59
28.70
f1 (ppm)

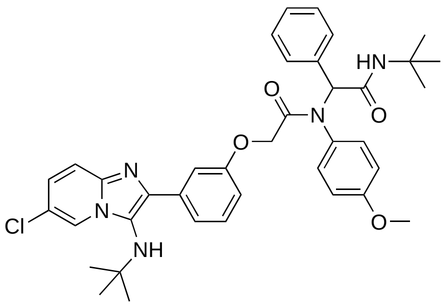

7a

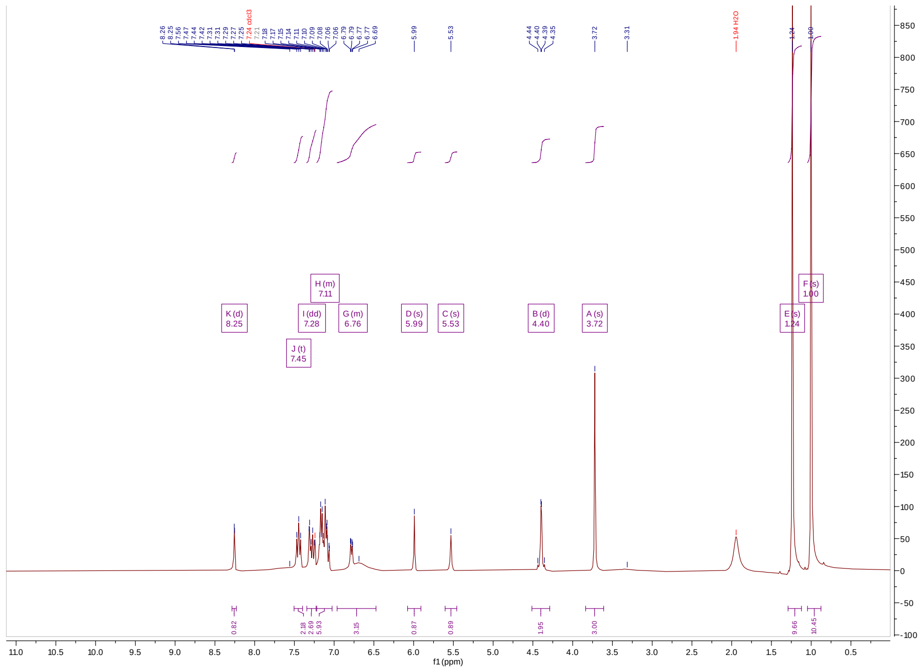
K (d)
8.25
J (t)
7.45
I (dd)
7.28
H (m)
7.11
G (m)
6.76
D (s)
5.99
C (s)
5.53
B (d)
4.40
A (s)
3.72
E (s)
1.24
F (s)
1.00
1.94 H2O
f1 (ppm)

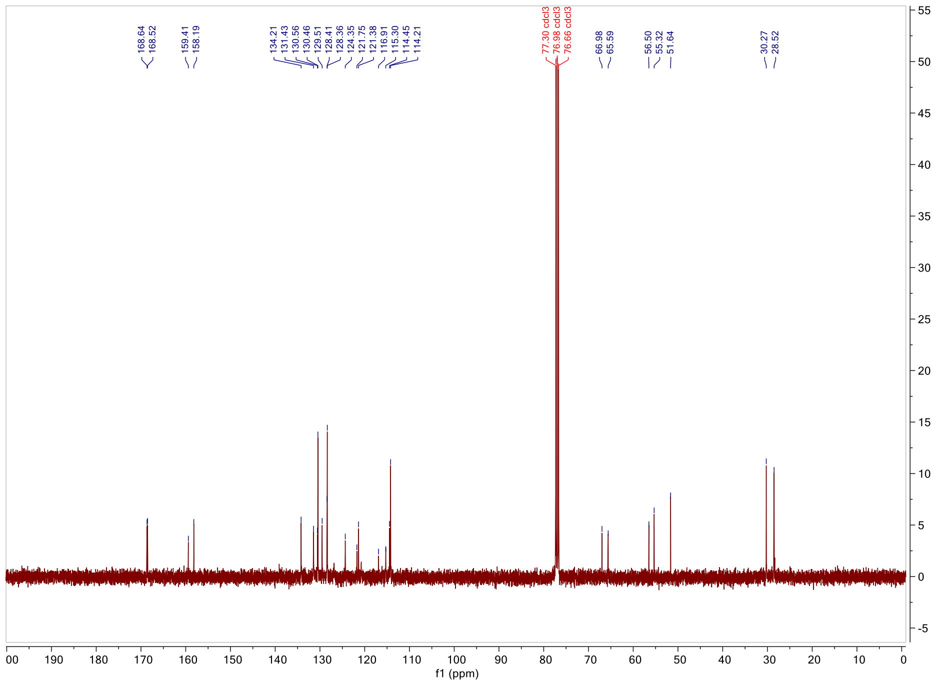
168.64
168.52
159.41
158.19
77.30 cdcl3
76.98 cdcl3
76.66 cdcl3
66.98
65.59
56.50
55.32
51.64
30.27
28.52
f1 (ppm)

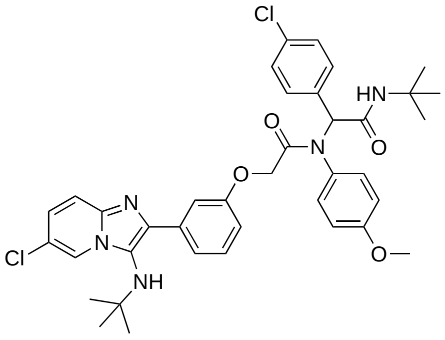

7b

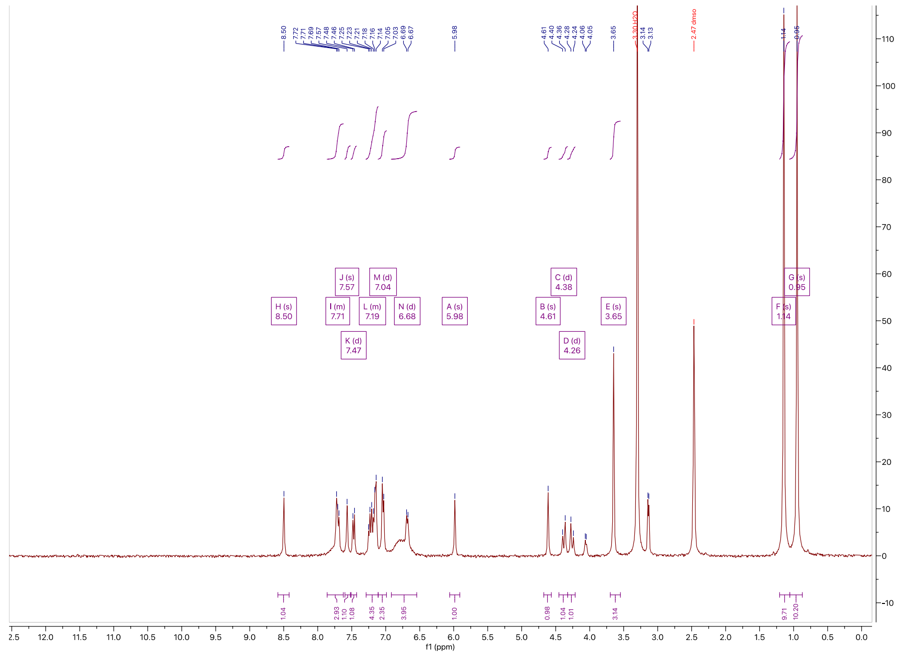

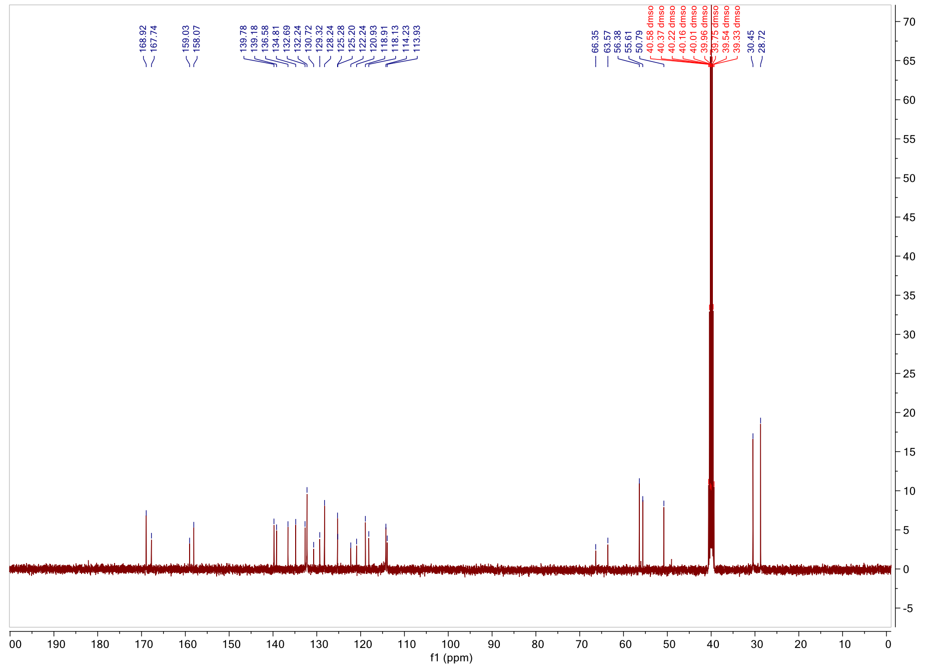

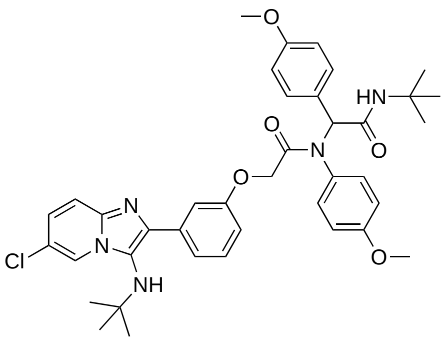
O
HN
O
N
O
O
N
N
Cl
NH
O

7c

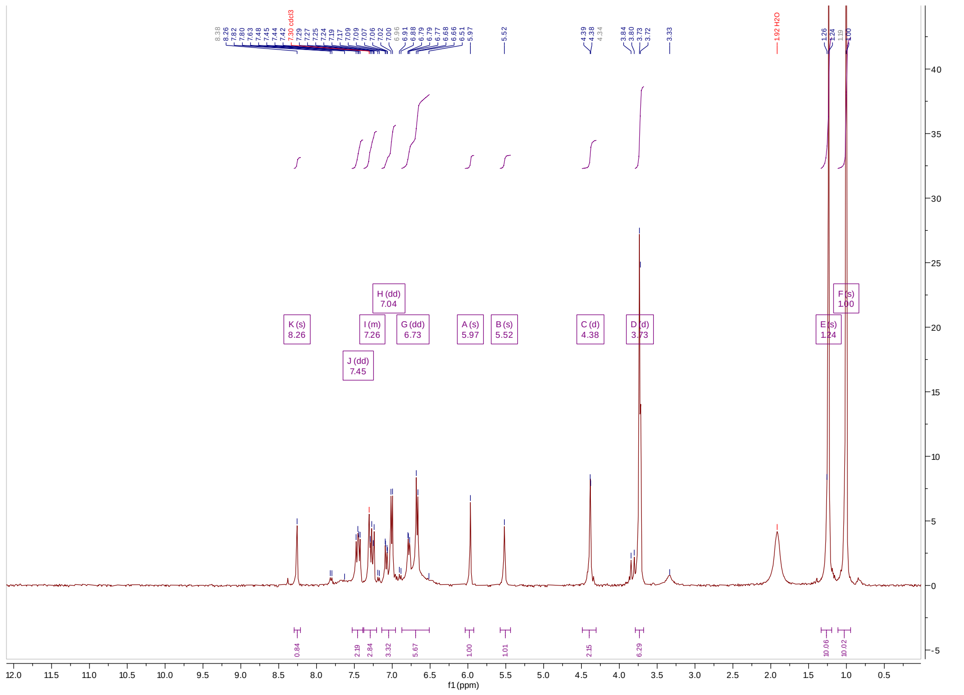
K (s)
8.26
I (m)
7.26
J (dd)
7.45
H (dd)
7.04
G (dd)
6.73
A (s)
5.97
B (s)
5.52
C (d)
4.38
D (d)
3.73
E (s)
1.24
F (s)
1.00
f1 (ppm)

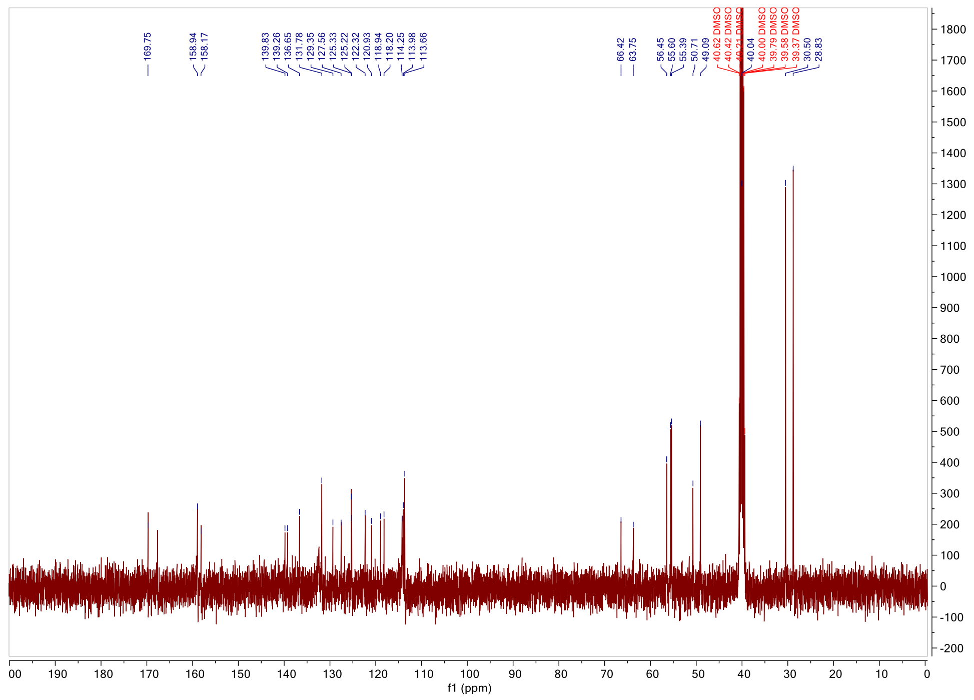
169.75
158.94
158.17
139.83
139.26
136.65
131.78
129.35
127.56
125.33
125.22
122.32
120.93
118.94
118.20
114.25
113.98
113.66
66.42
63.75
56.45
55.60
55.39
50.71
49.09
40.62 DMSO
40.42 DMSO
40.04
40.00 DMSO
39.79 DMSO
39.58 DMSO
39.37 DMSO
30.50
28.83
f1 (ppm)

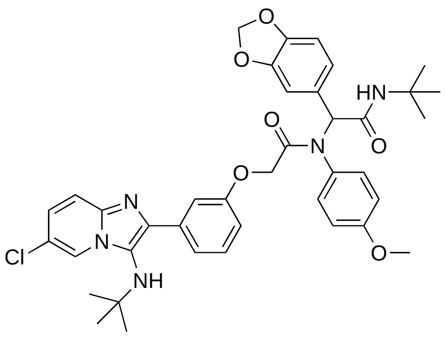

7d

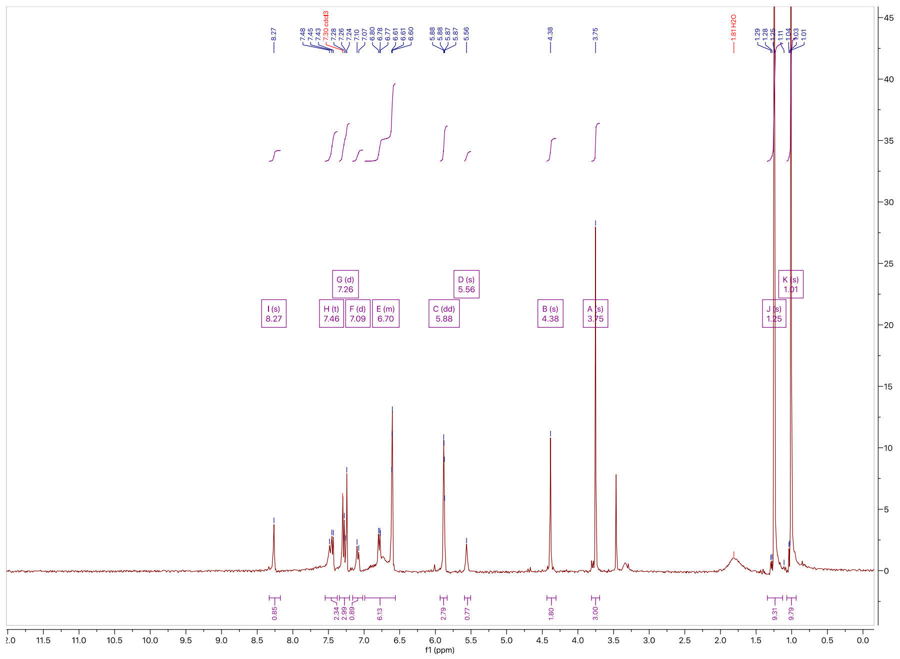

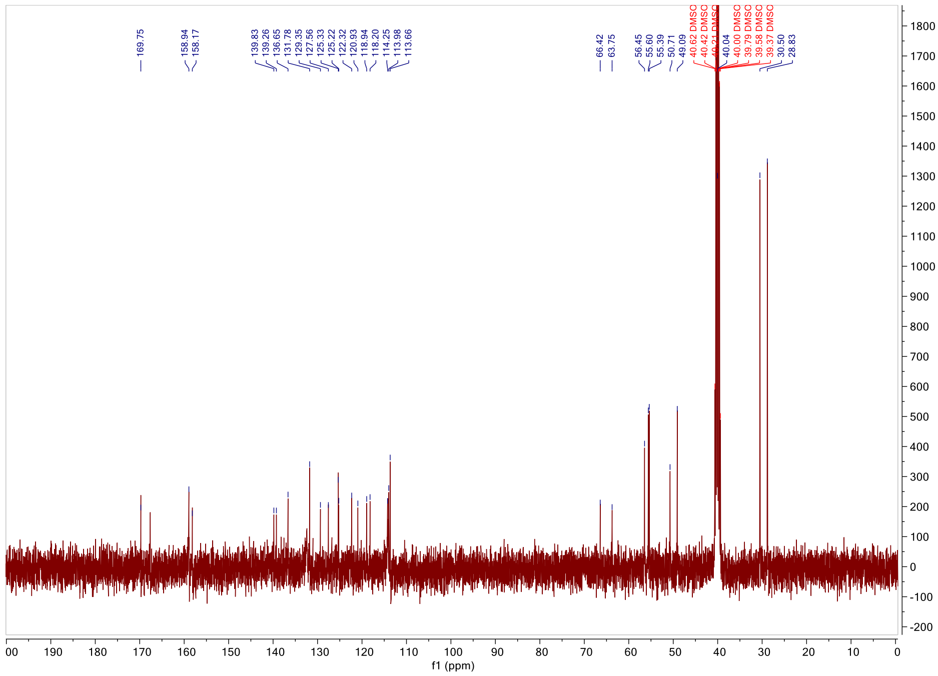

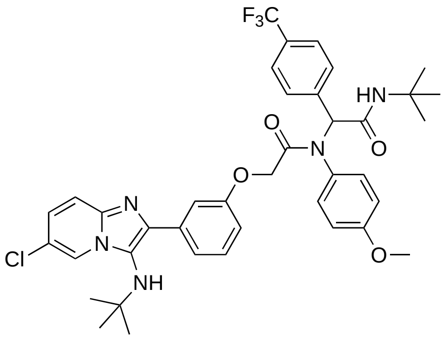

7e

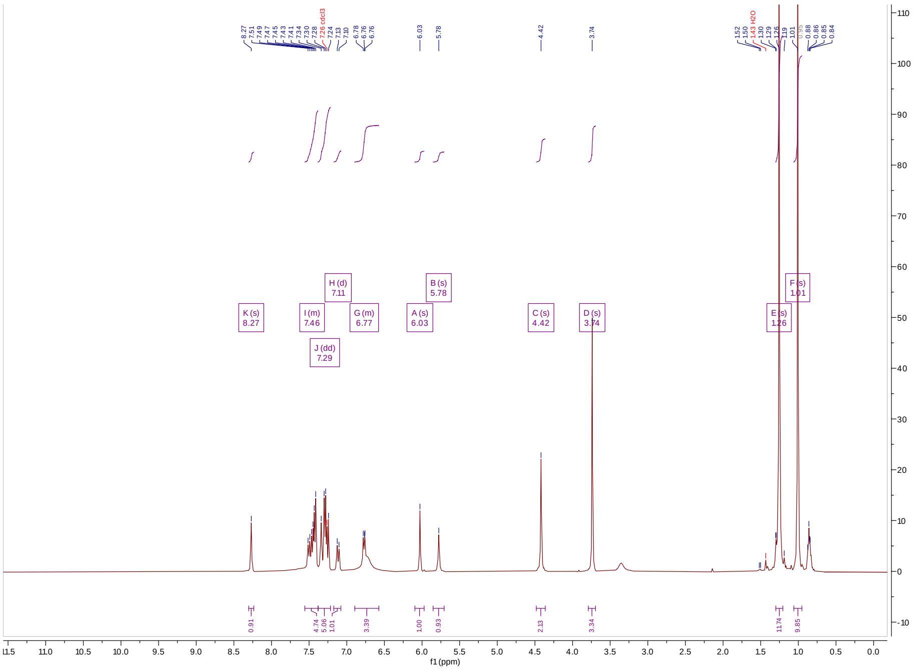

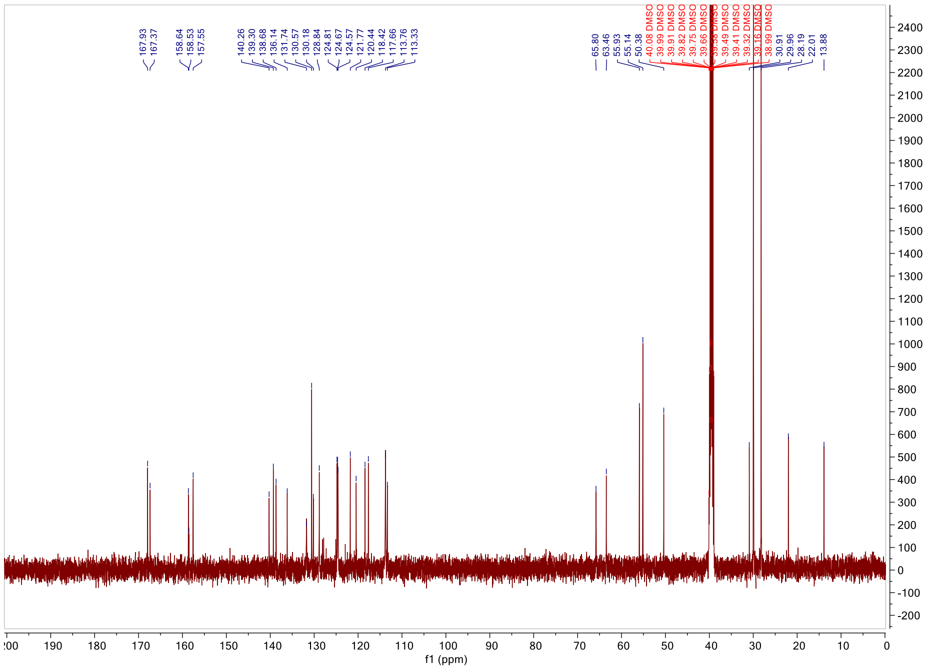

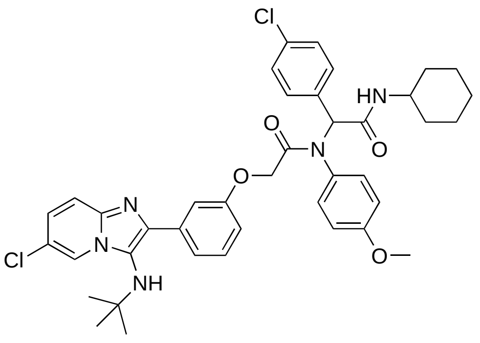

7f

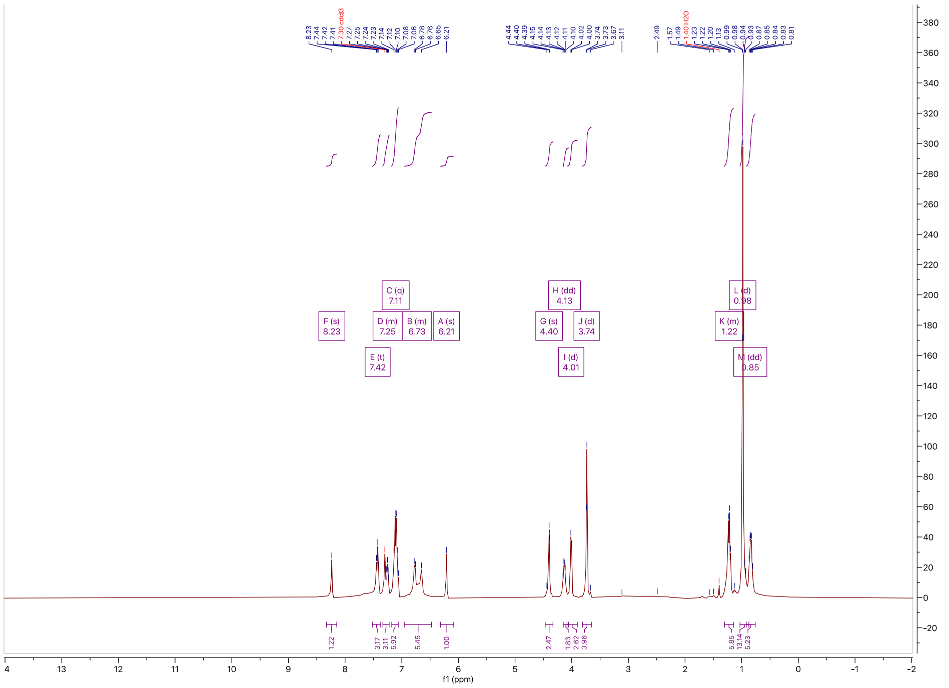

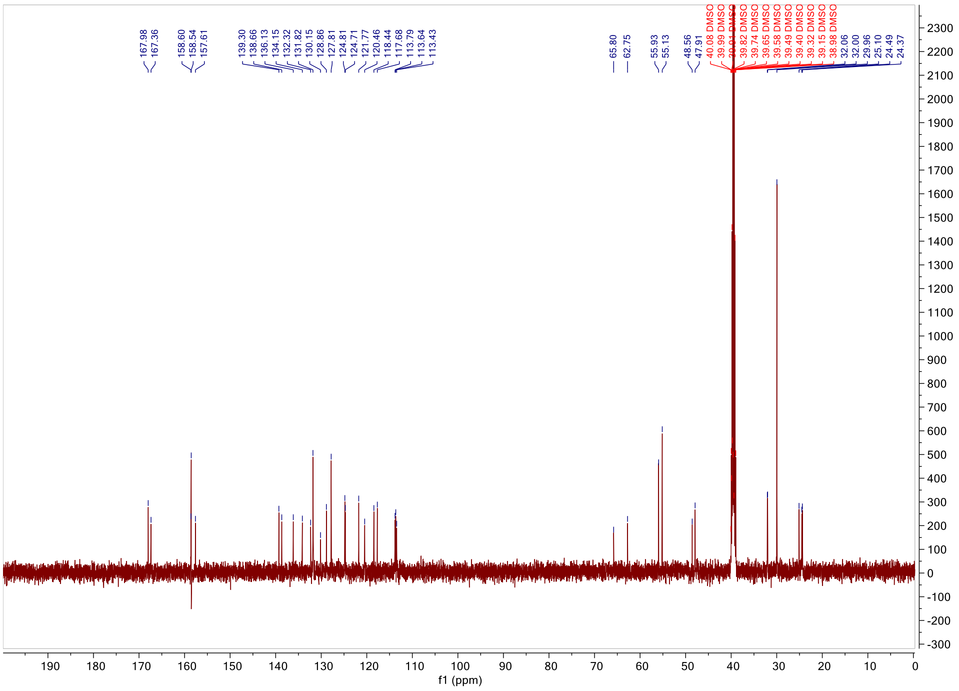

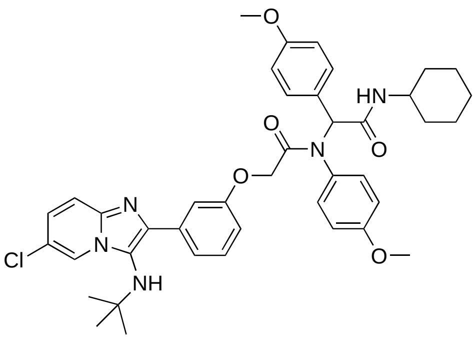

7g

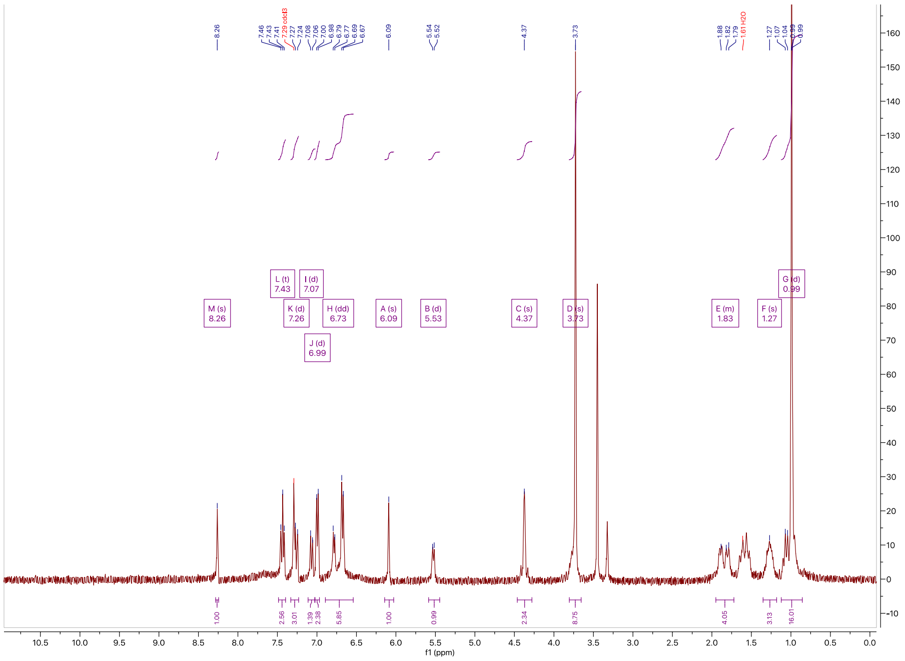

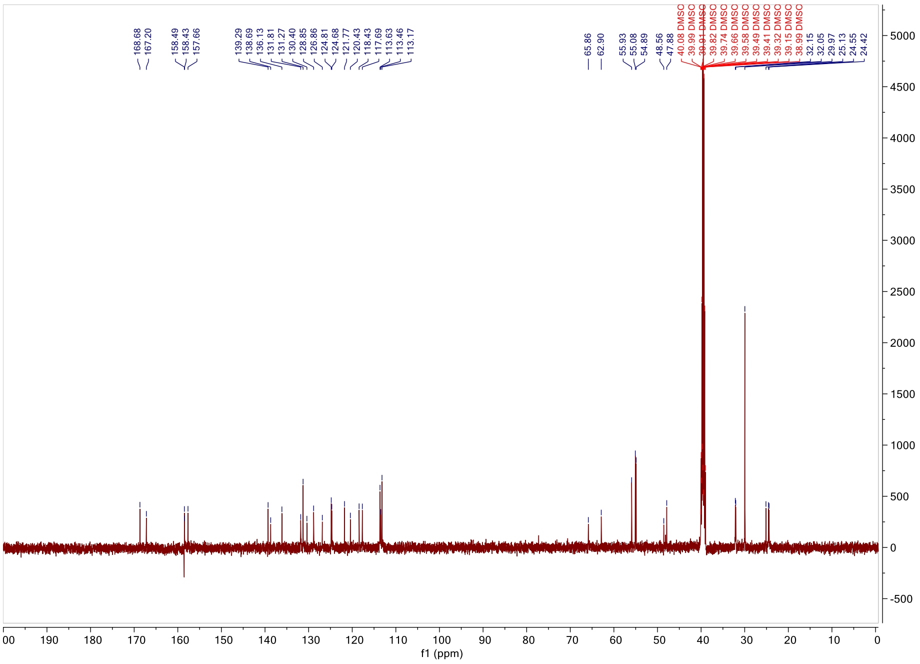

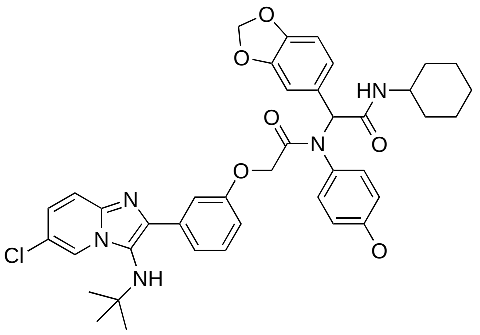

7h

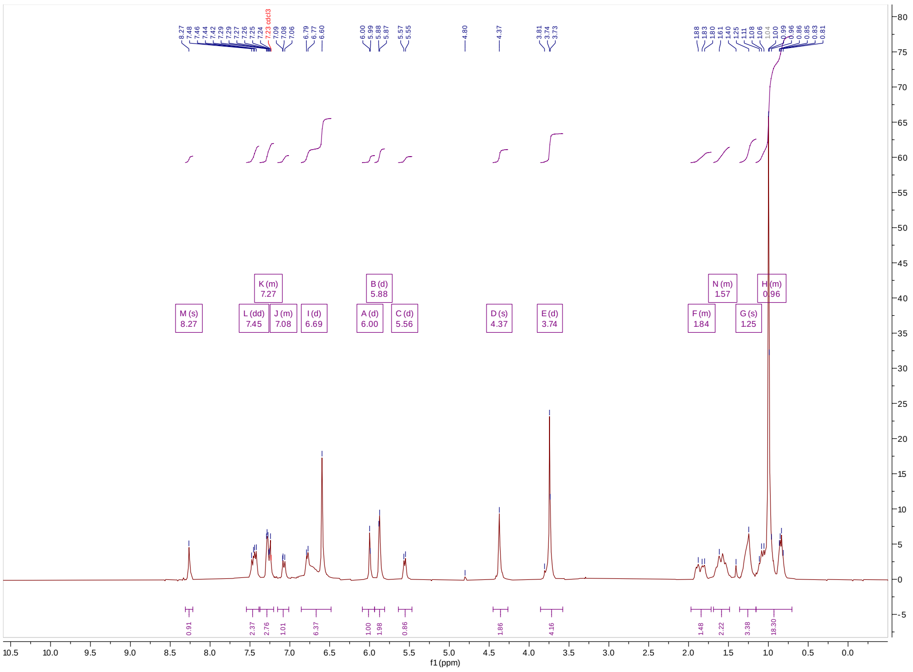

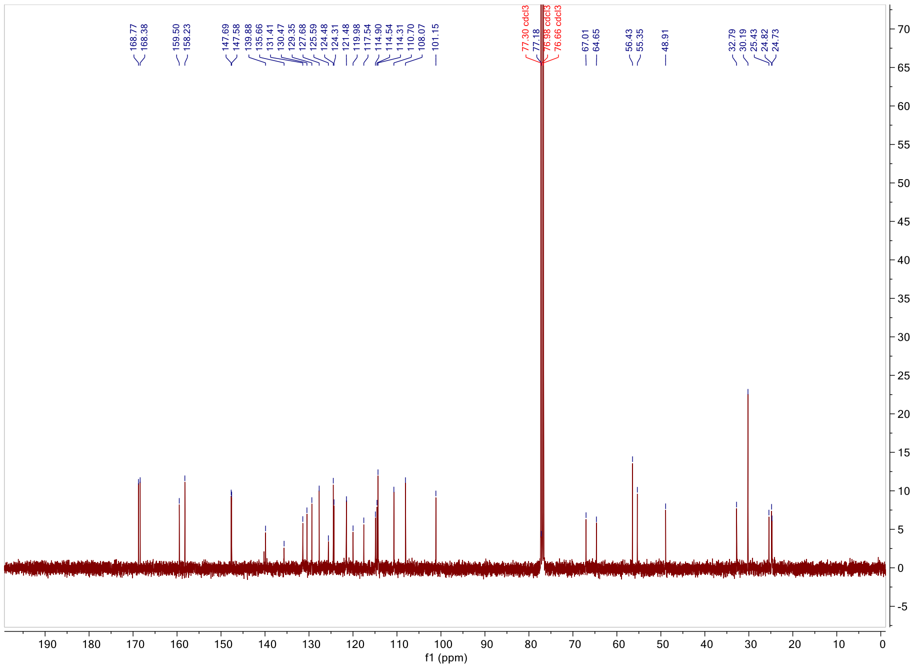

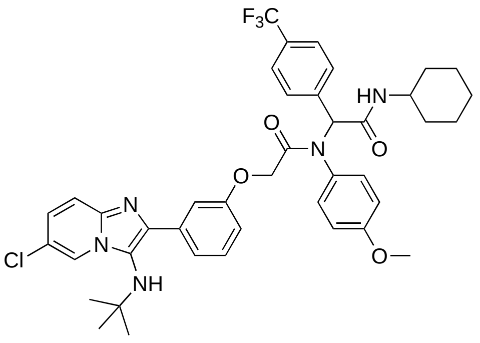

7i

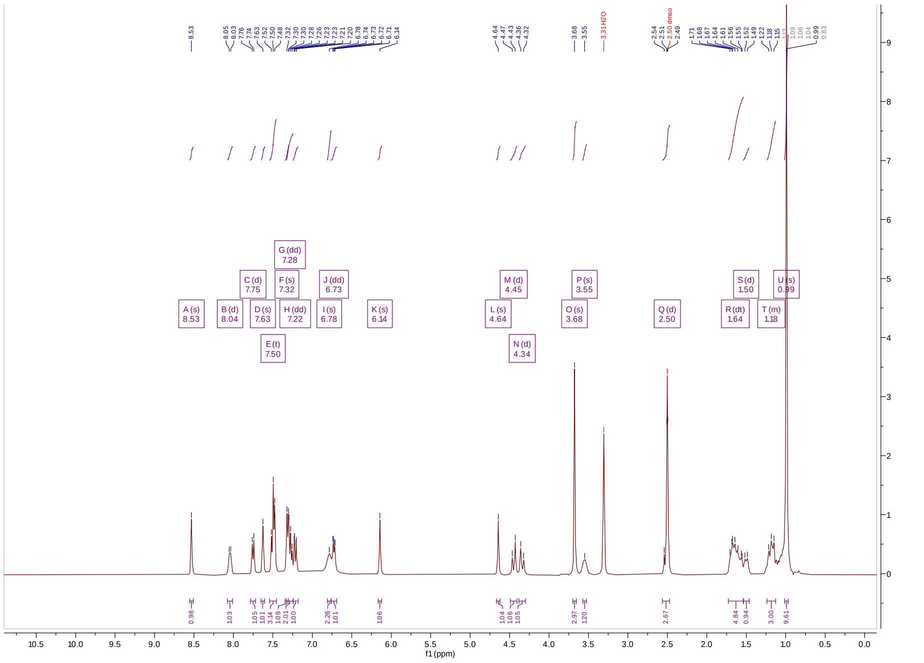

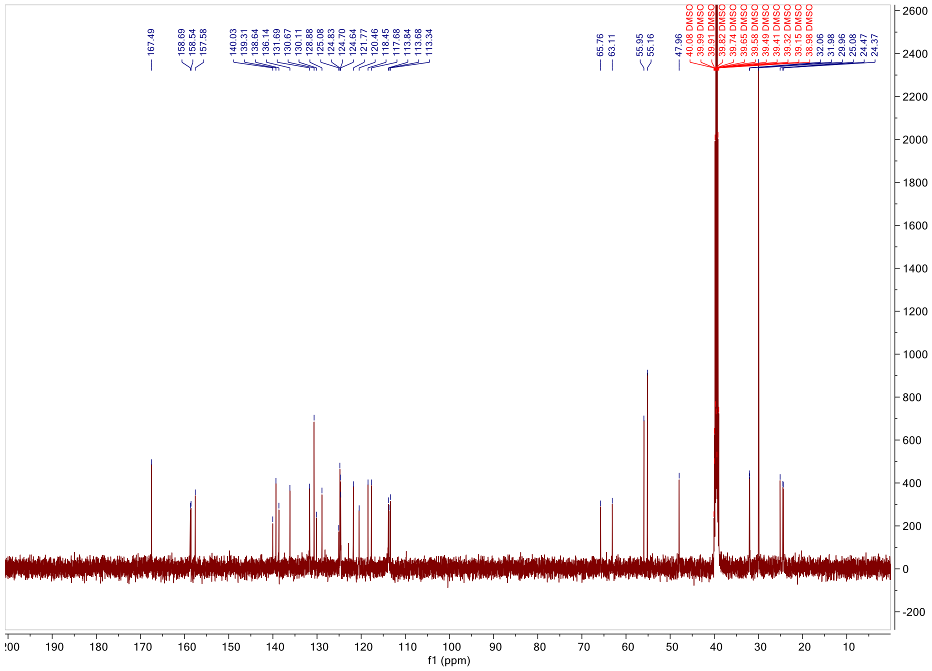

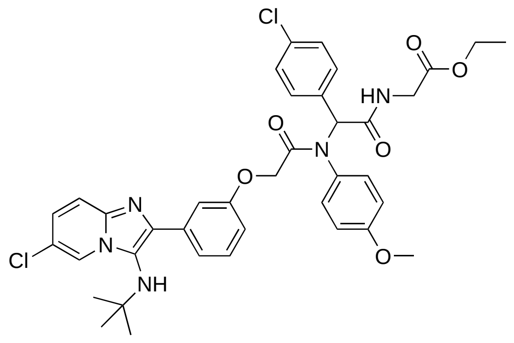

7j

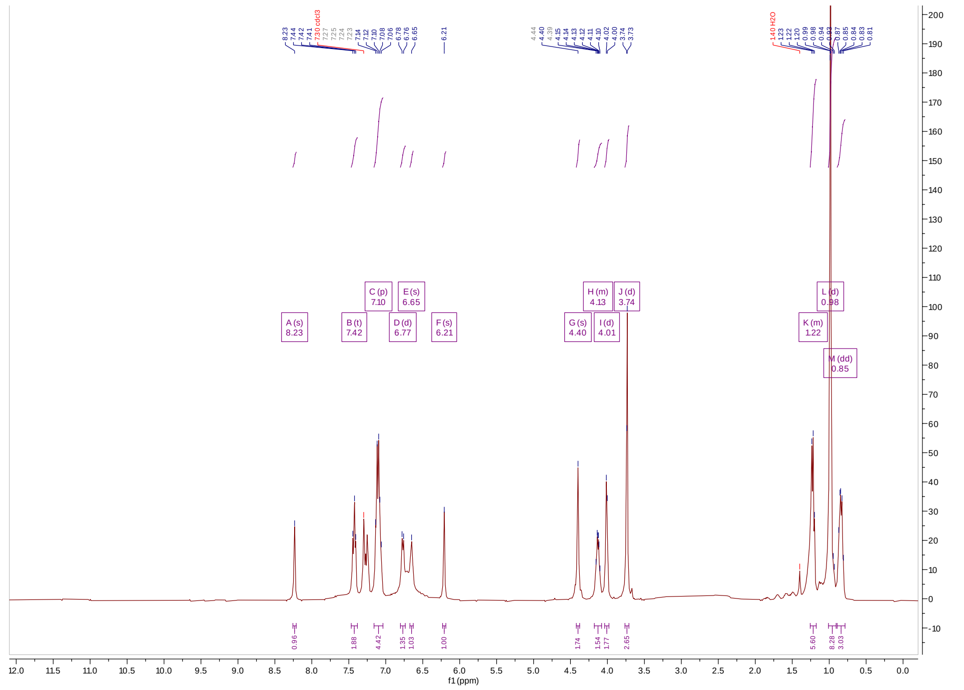

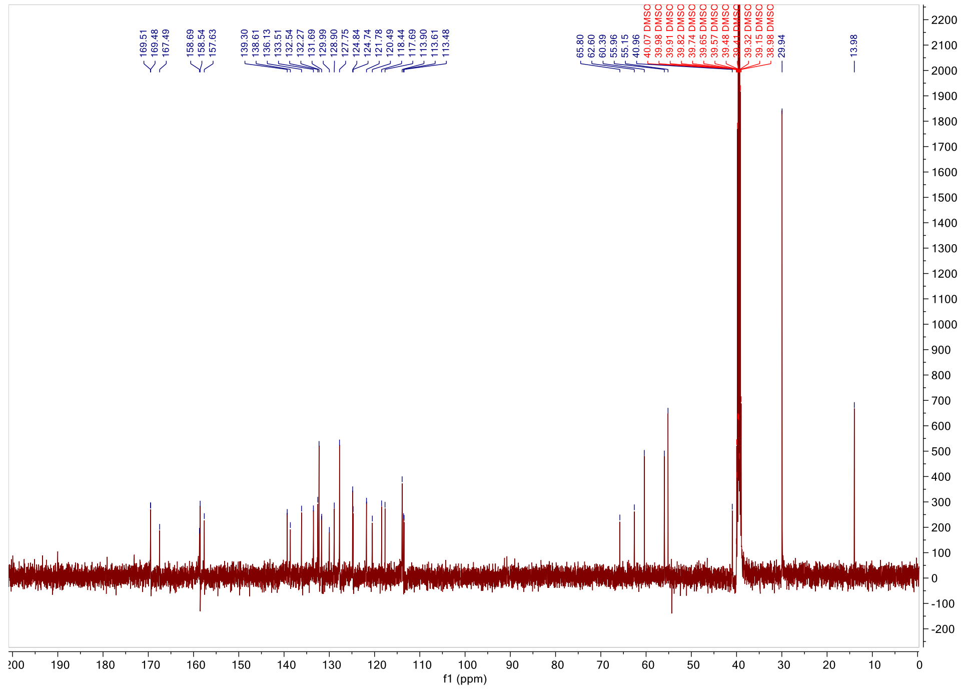

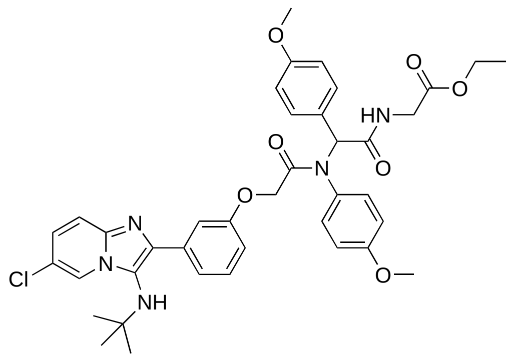

7k

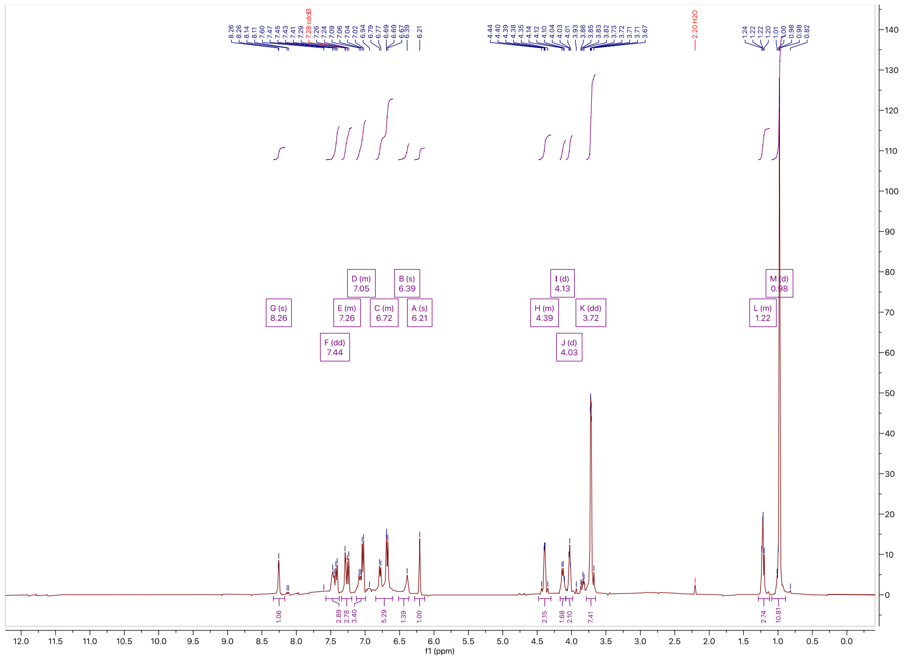

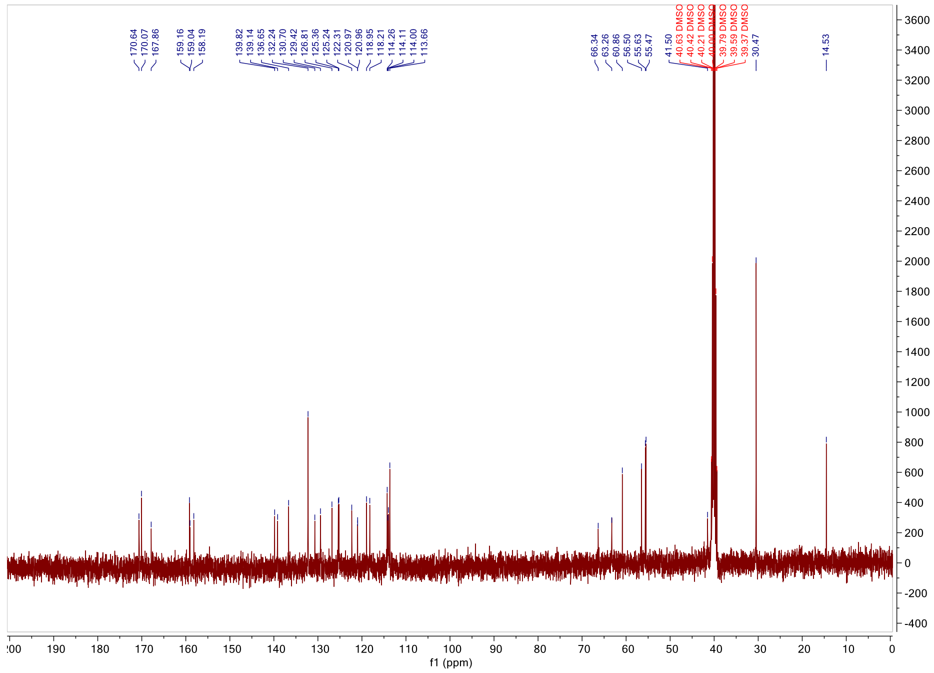

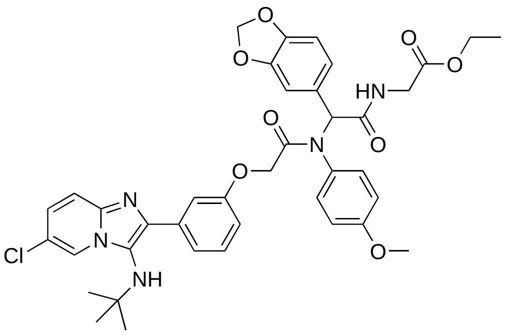

7l

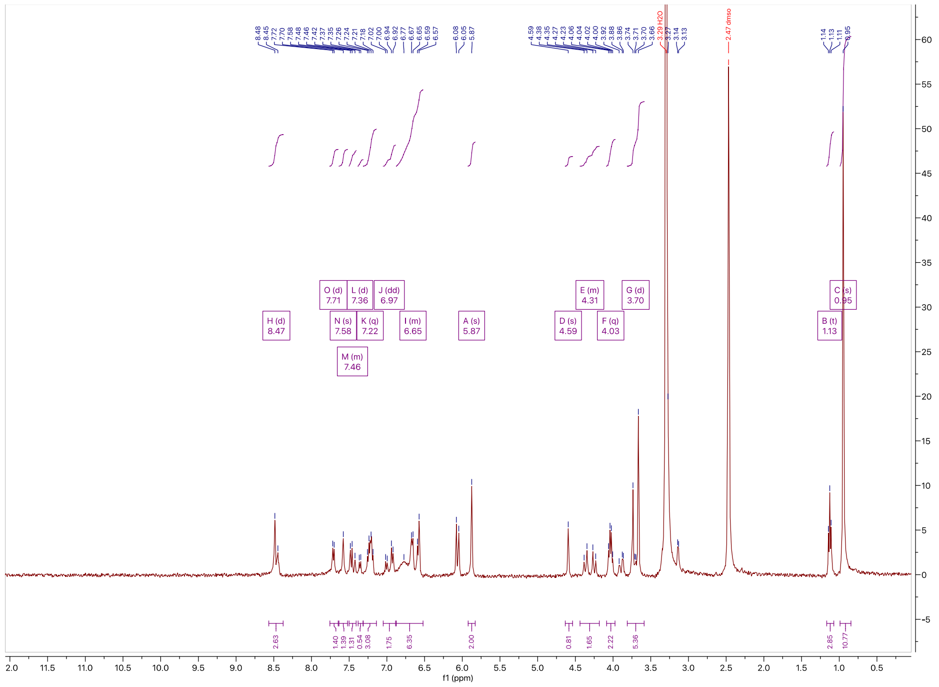

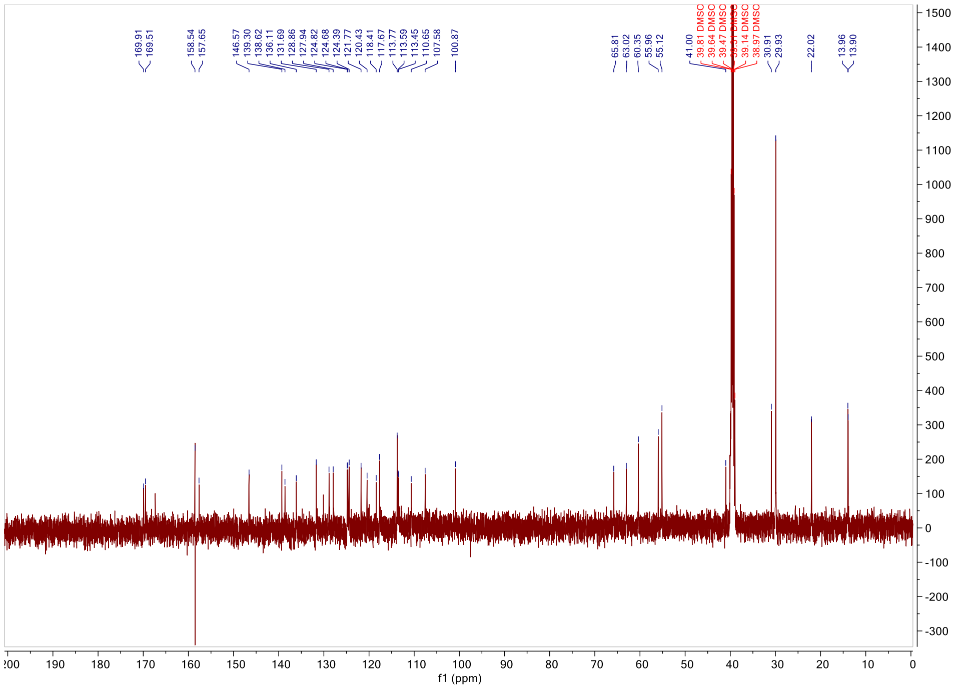

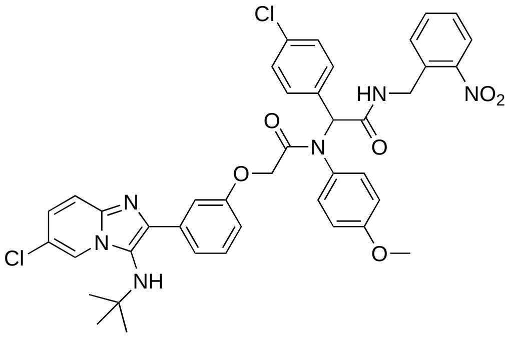

7m

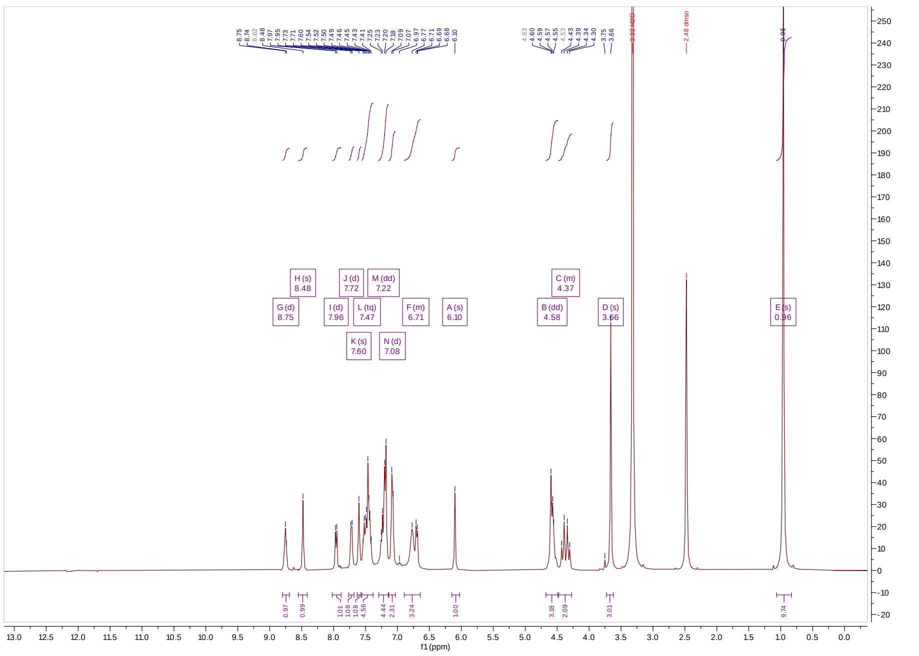
H (s) 8.48
J (d) 7.72
M (dd) 7.22
C (m) 4.37
G (d) 8.75
I (d) 7.96
L (tq) 7.47
F (m) 6.71
A (s) 6.10
B (dd) 4.58
D (s) 3.66
E (s) 0.96
K (s) 7.60
N (d) 7.08
3.33 H2O
2.48 dmso
f1 (ppm)

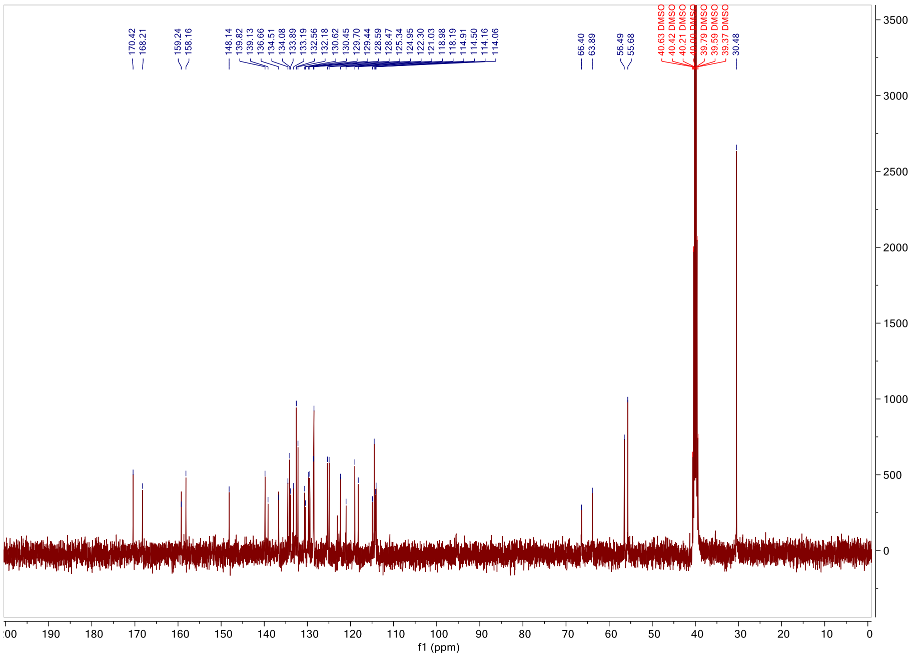
170.42
168.21
159.24
158.16
148.14
139.82
139.13
136.66
134.51
134.08
133.89
133.19
132.56
132.18
130.62
130.45
129.70
129.44
128.59
128.47
125.34
124.95
122.30
121.03
118.98
118.19
114.91
114.50
114.16
114.06
66.40
63.89
56.49
55.68
40.63 DMSO
40.42 DMSO
40.21 DMSO
40.00 DMSO
39.79 DMSO
39.58 DMSO
39.37 DMSO
30.48
f1 (ppm)

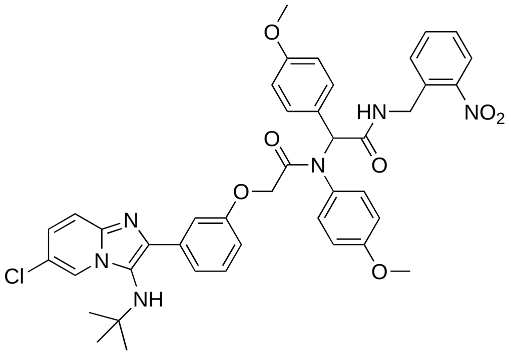

7n

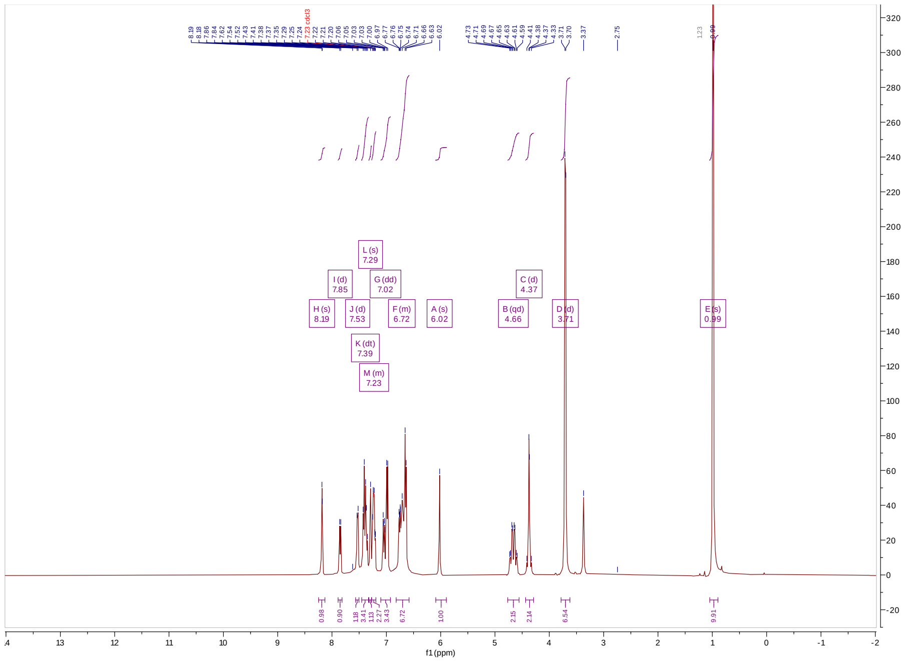

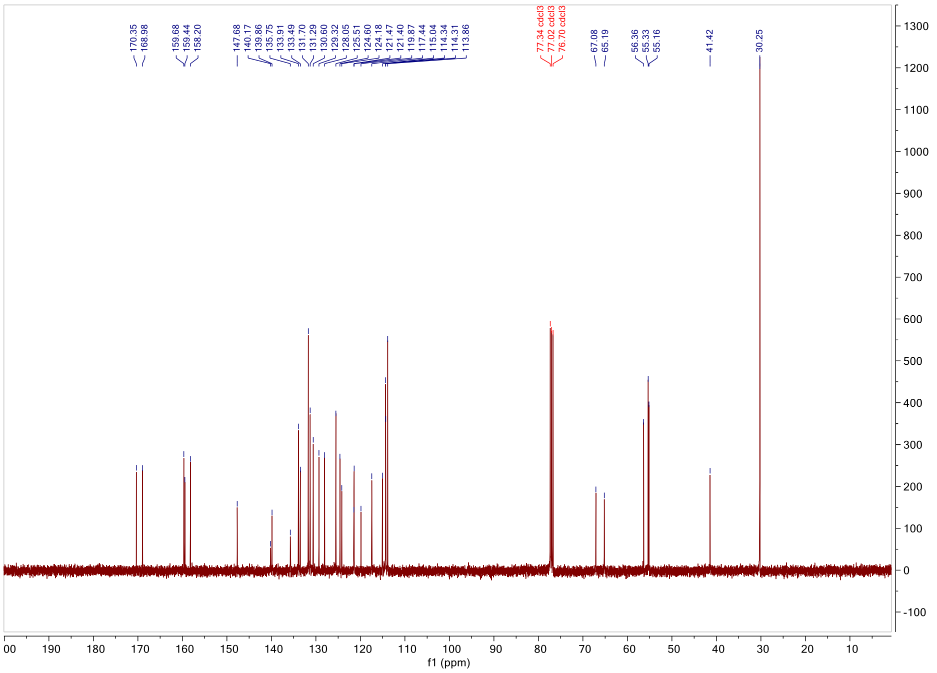

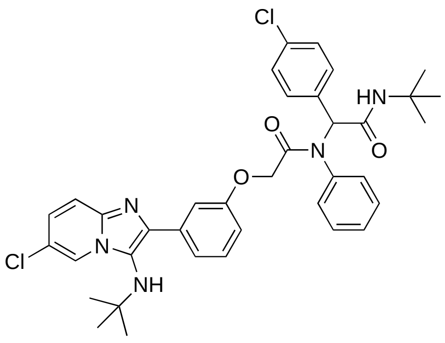

7o

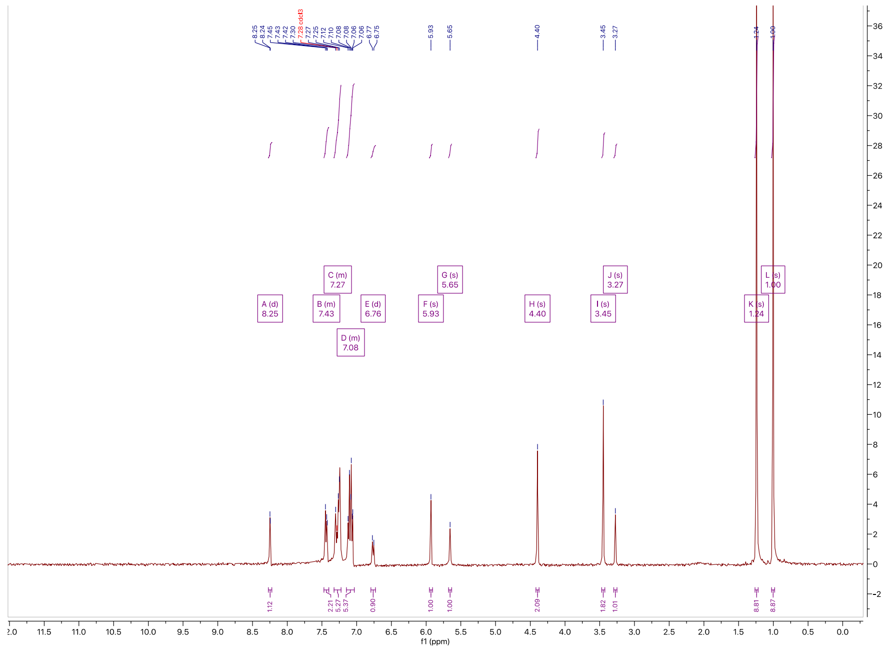

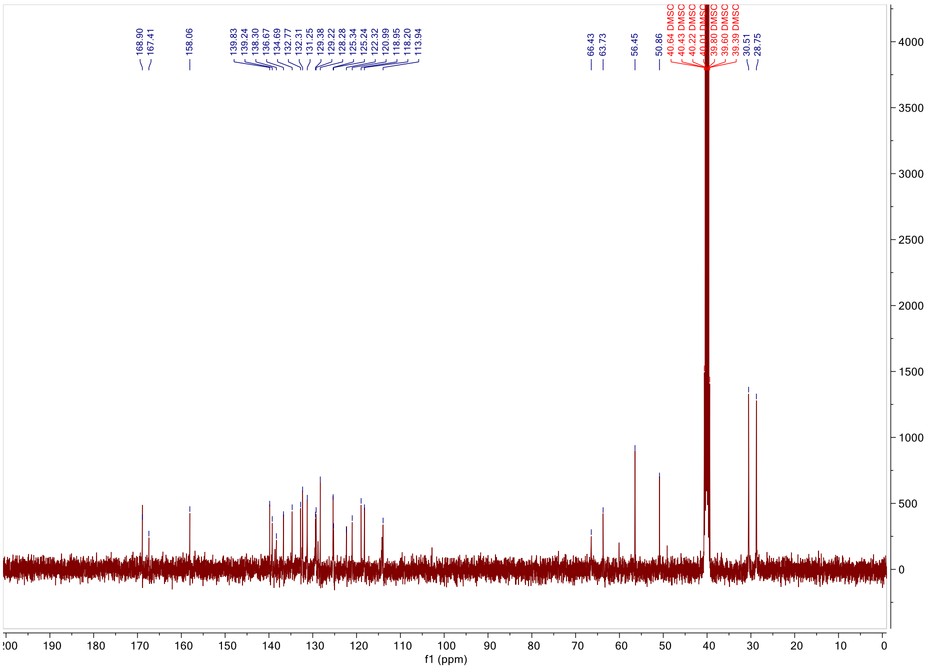

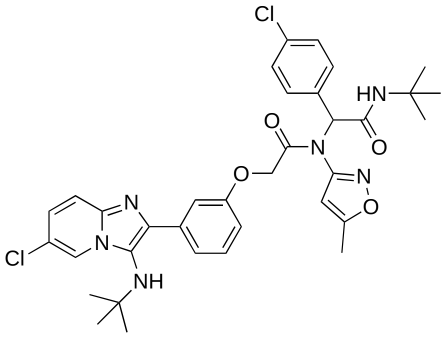

7p

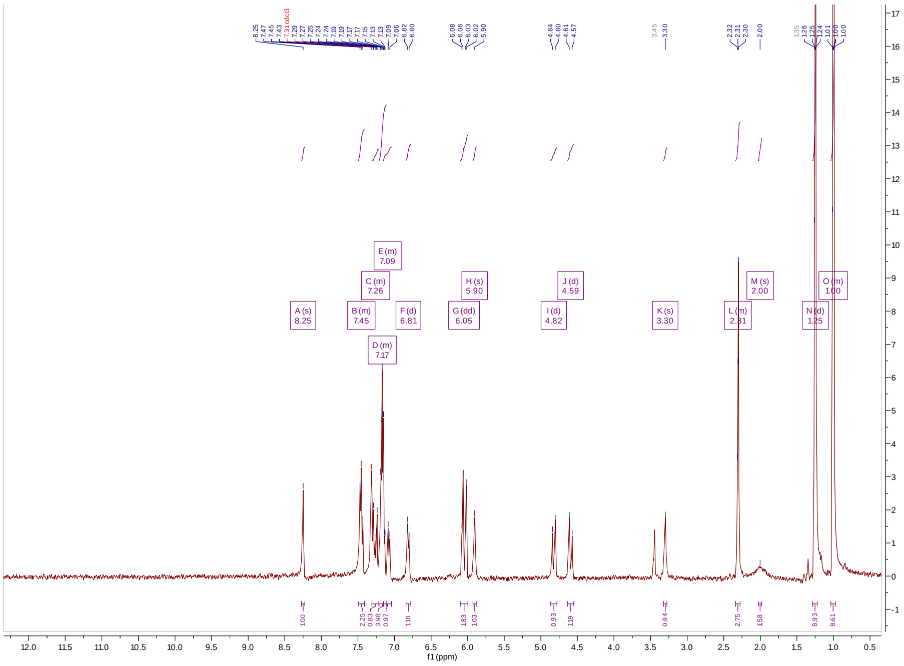

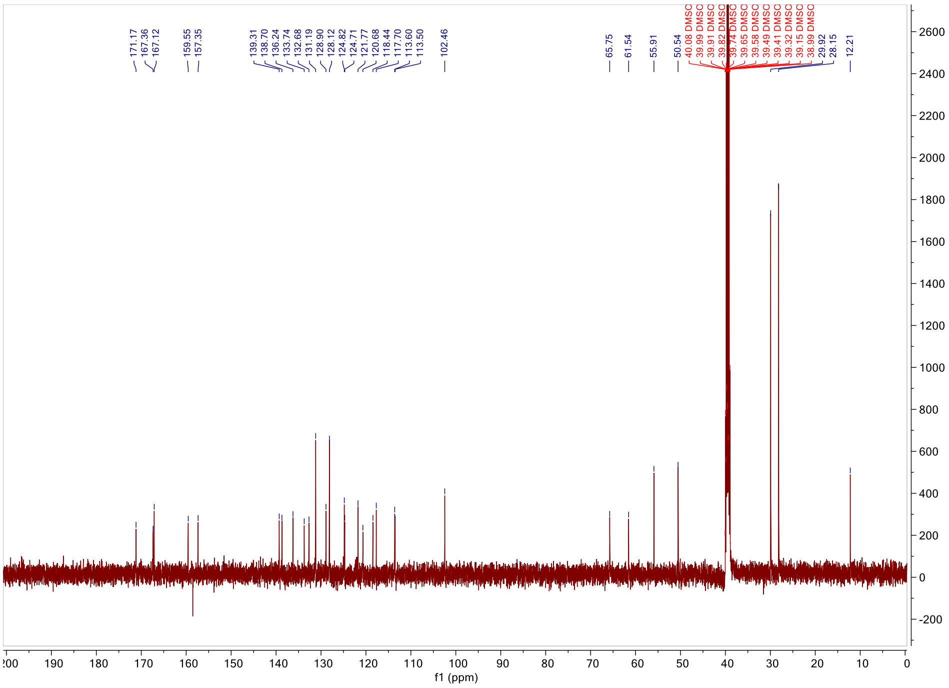

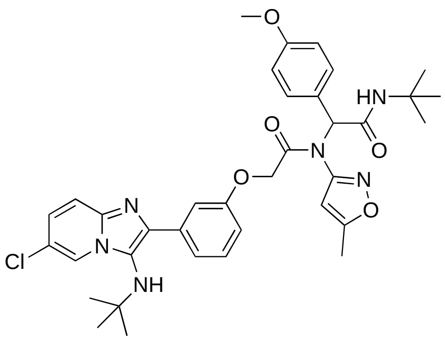

7q

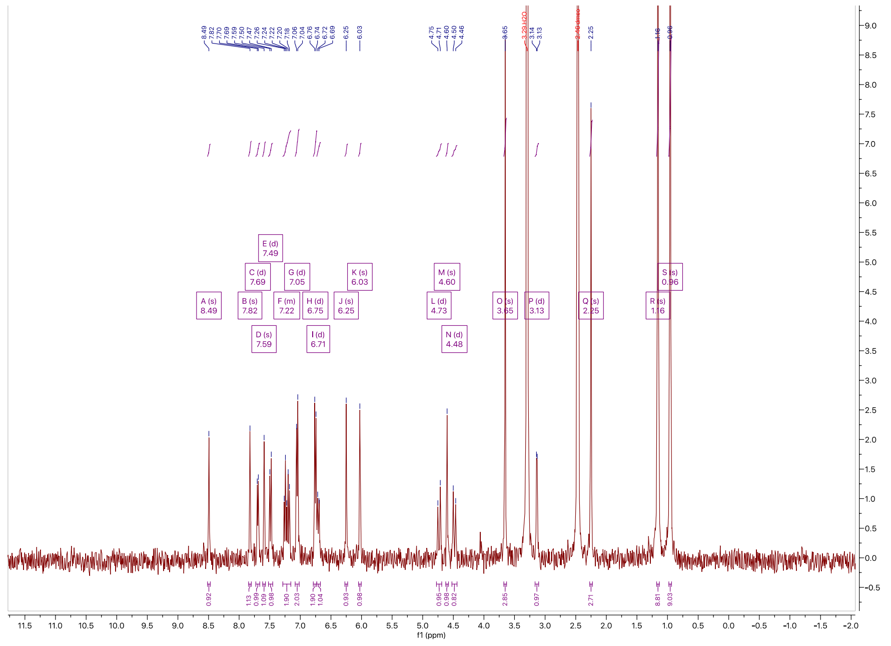

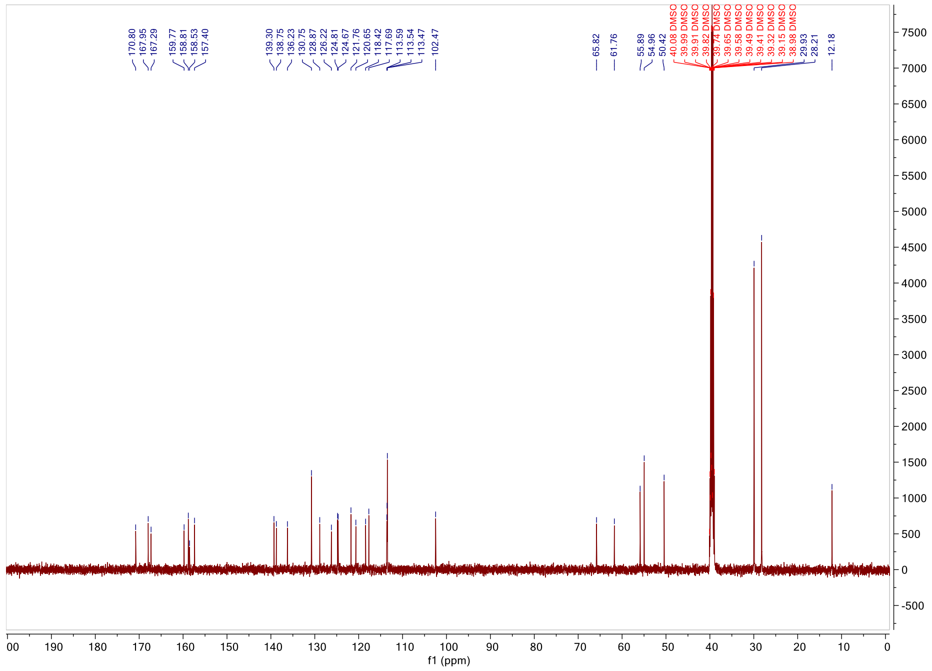

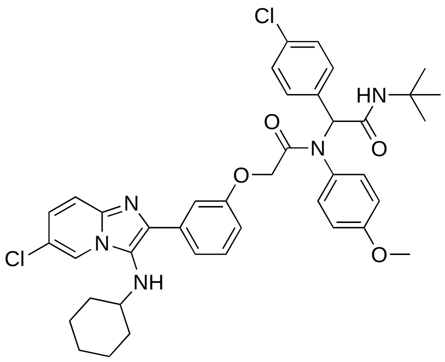

7r

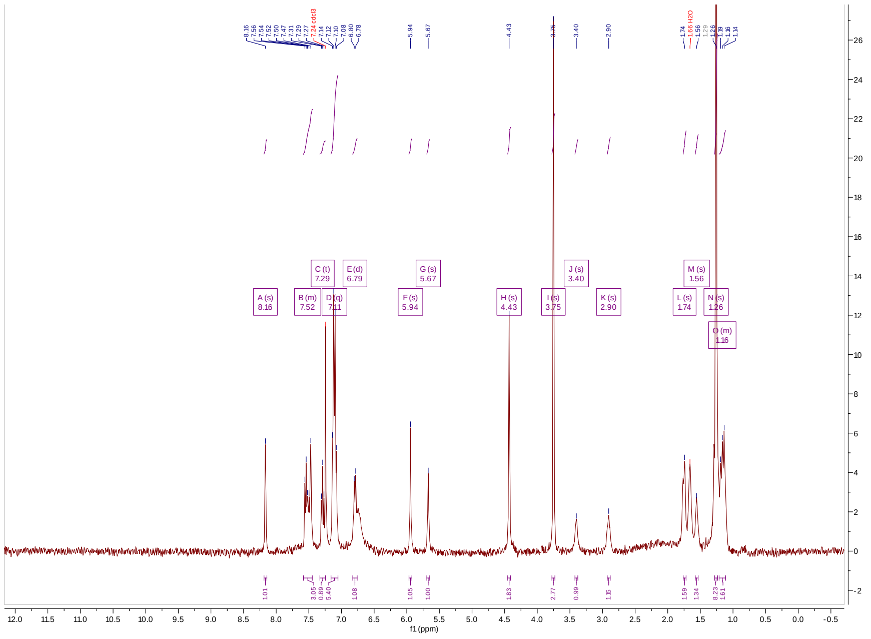

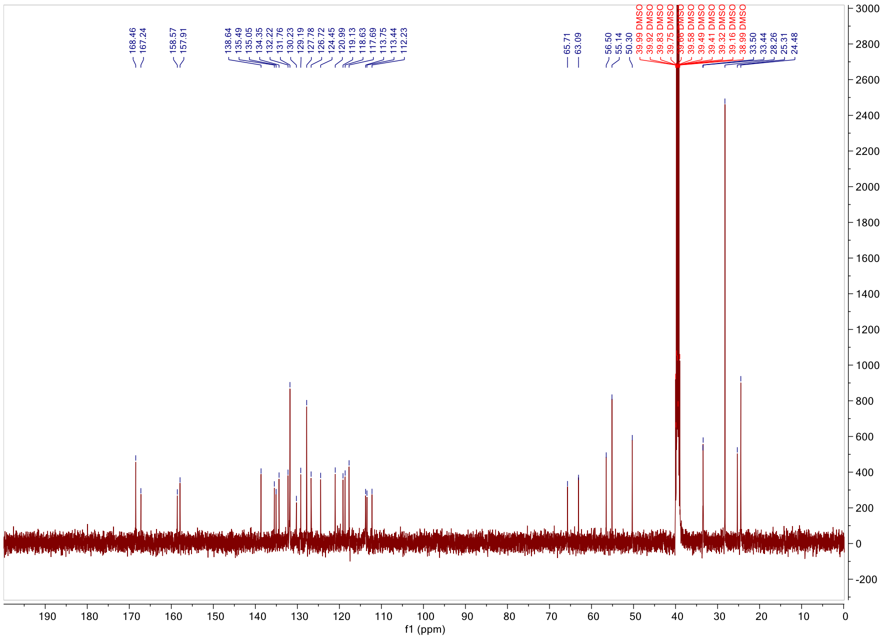

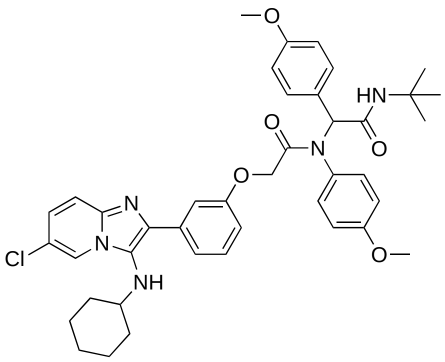

7s

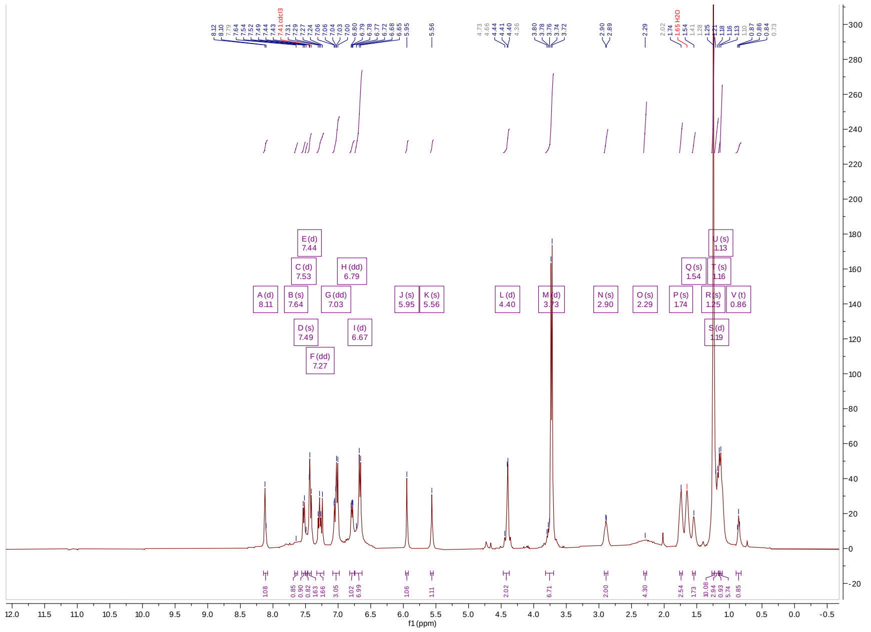

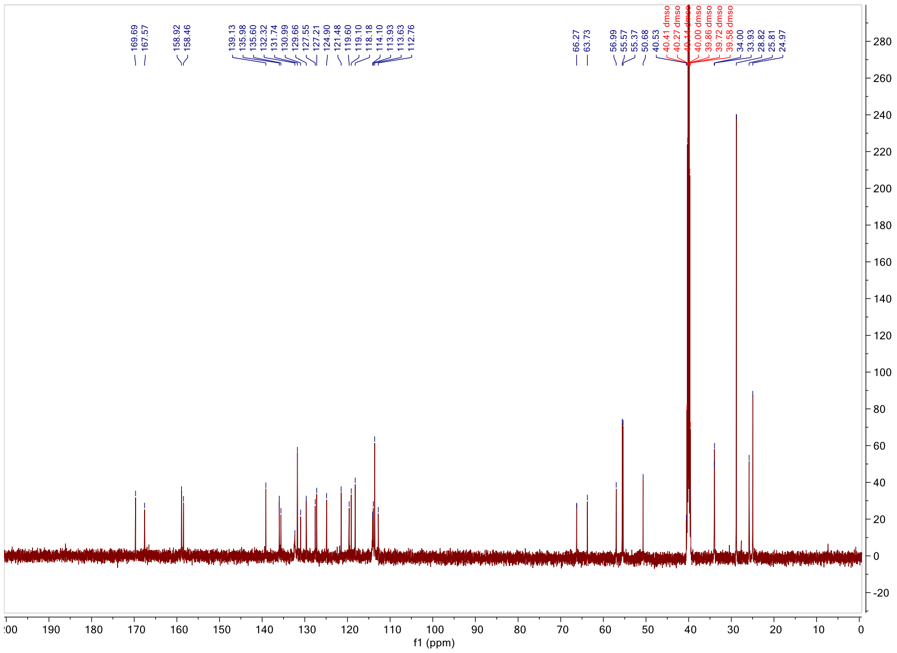

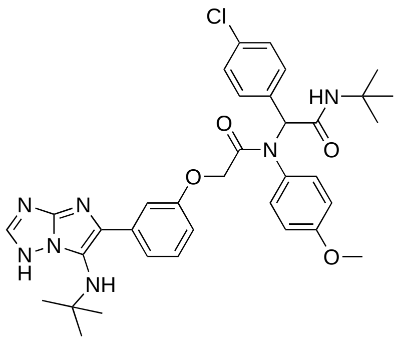
Cl
HN
O
O
O
N
N
N
N
N
H
NH
O

7t

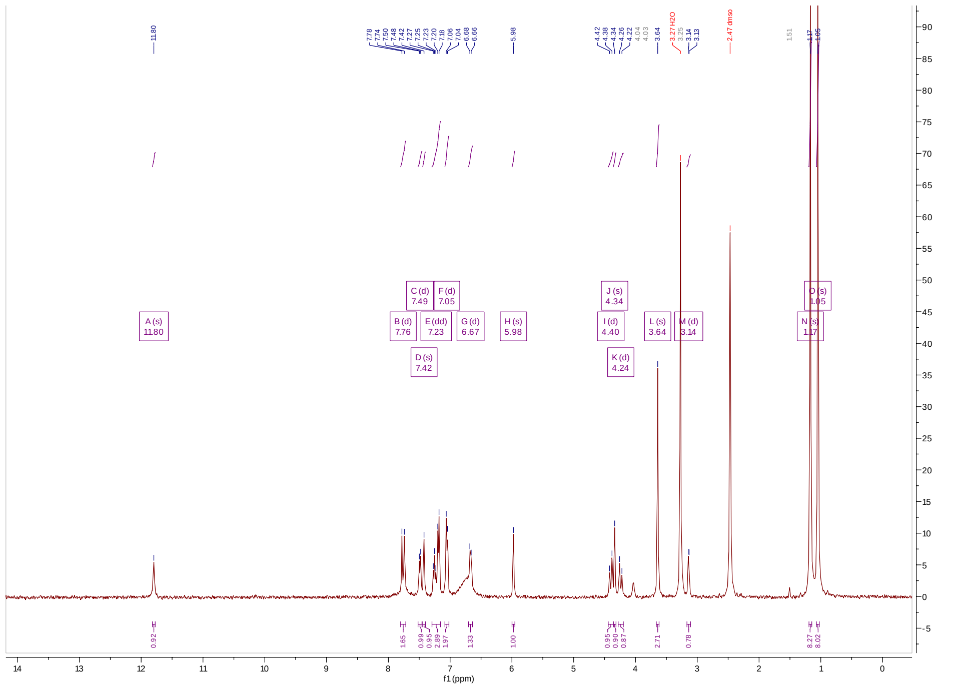
11.80
7.78 7.74 7.50 7.48 7.42 7.27 7.25 7.23 7.23 7.20 7.18 7.06 7.04 6.68 6.66
5.98
4.42 4.38 4.34 4.26 4.22 4.04 4.03
3.64
3.27 H2O
3.14 3.13
2.47 dmso
1.51
1.17 1.05
A (s) 11.80
B (d) 7.76
C (d) 7.49
D (s) 7.42
E (dd) 7.23
F (d) 7.05
G (d) 6.67
H (s) 5.98
I (d) 4.40
J (s) 4.34
K (d) 4.24
L (s) 3.64
M (d) 3.14
N (s) 1.17
O (s) 1.05
0.92
1.65
0.99 0.95 0.95 2.80 1.97
1.33
1.00
0.95 0.90 0.87
2.71
0.78
8.27 8.02
f1 (ppm)

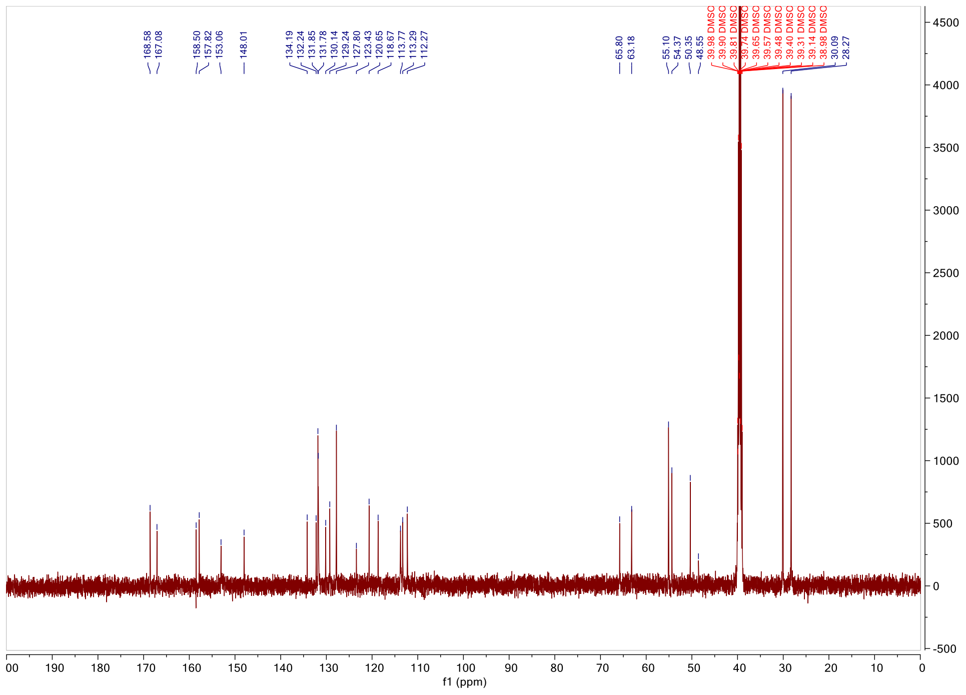
168.58
167.08
158.50
157.82
153.06
148.01
134.19
132.24
131.85
131.78
130.14
129.24
127.80
123.43
120.65
118.67
113.77
113.29
112.27
65.80
63.18
55.10
54.37
50.35
48.55
39.98 DMSO
39.90 DMSO
39.81 DMSO
39.74 DMSO
39.65 DMSO
39.57 DMSO
39.48 DMSO
39.40 DMSO
39.31 DMSO
39.14 DMSO
38.98 DMSO
30.09
28.27
f1 (ppm)

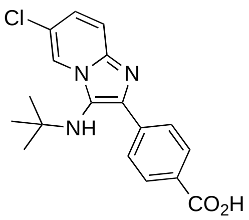

8a

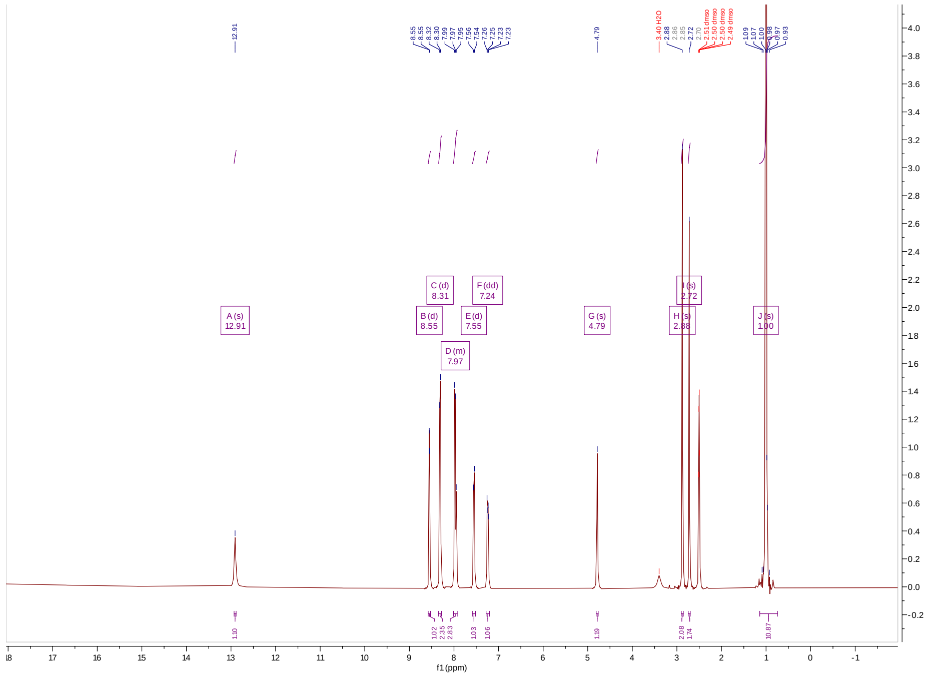

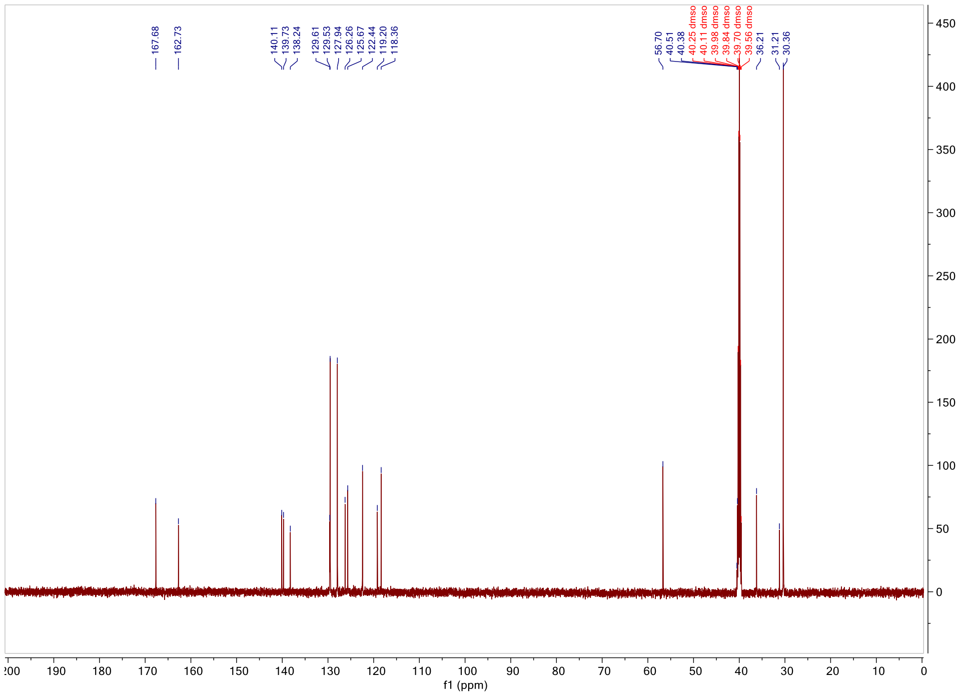

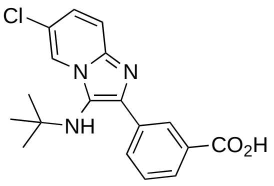

8b

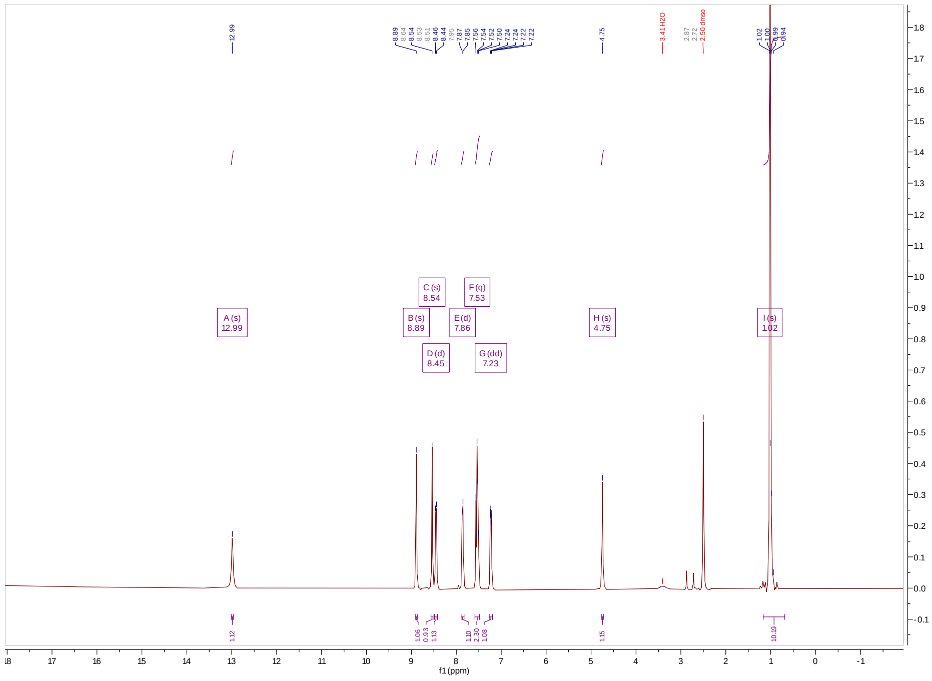

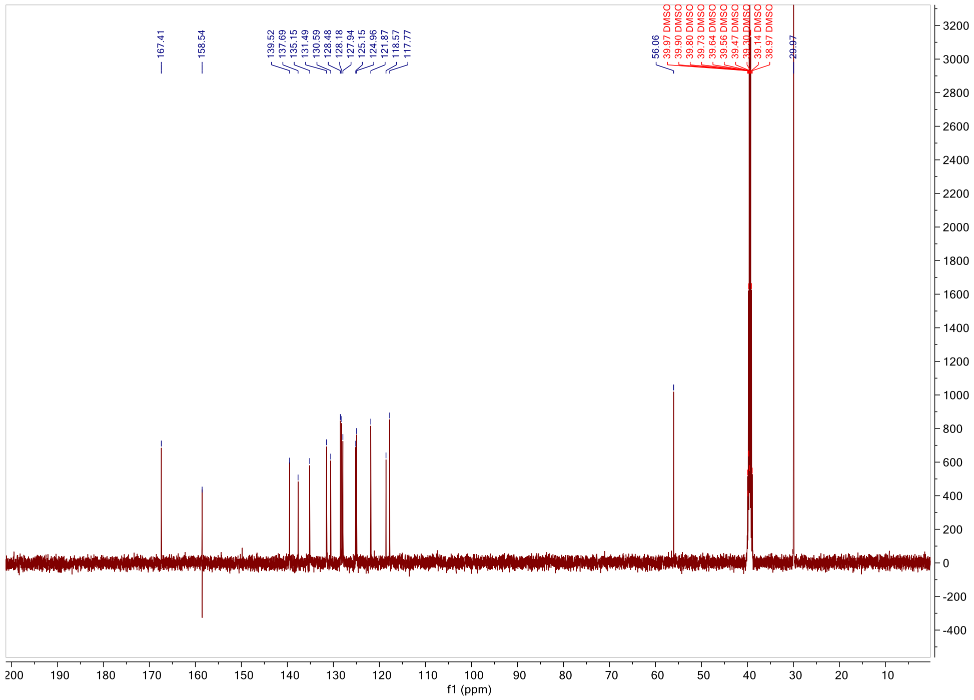

4. Measurement of antibacterial activity

To evaluate the spectrum of antibacterial action of compounds, the value of the minimum concentration that inhibited the growth of microorganisms (MIC) was determined by the micro method of serial two-fold dilutions in Mueller–Hinton Broth (Biolife Italiana Srl, Italy). Disposable polystyrene 96-well tablets for immunological studies (Biosigma, Italy) and mechanical 8-channel Proline Plus dispensers were used in the experiment. Analyzes were performed by CLSI (Clinical and Laboratory Standards Institute, USA) recommendations [3-6].

In the beginning, the studied compounds were introduced into the wells of the tablet, the concentration of which, in a total medium volume of 200 µl, was 500; 250; 125; 62.5; 31.25 and 15.6 µg/mL. Collection reference strains of bacteria were used as a culture test: Escherichia coli (strain ATCC 25922), Pseudomonas aeruginosa (strain ATCC 27853) – gram-negative cultures, Bacillus subtilis (strain ATCC 6633), Staphylococcus aureus (strain ATCC 25923) – gram-positive cultures. To prepare the inoculum from isolated colonies of microorganisms that were in the exponential phase of growth, a suspension was prepared according to the McFarland standard (McFarland) 0.5 in physiological solution. The suspension was diluted in Muller–Hinton broth to a concentration of 105 CFU/mL and 100.0 µL was added to wells of the tablet containing 100.0 µL of the appropriate dilutions of the compounds under study, and to one well with 100.0 µL of nutrient broth without drugs as " negative control". The sterility of the medium was monitored by the wells of the microtiter plate specially selected for this purpose, in which solutions of compounds and microbial suspension are not introduced, the inoculated plates were incubated for 18–24 hours at 37 °C. Each experiment was carried out in triplicate.

The results were recorded visually and spectrophotometrically, comparing the growth of the microorganism in the presence of the studied compound with the growth of the culture in the well without it. The minimum bacteriostatic concentration (MPC) was taken to be the smallest amount of substance, expressed in μg/ml, in the presence of which suppression (inhibition) of culture growth occurred (no visible growth of microorganisms). The minimum bactericidal concentration was determined by sowing the contents of test tubes with no signs of growth on meat-peptone agar in Petri dishes. The optical density of the medium during the growth of cultures was measured using a Sunrise RS microplate photometer (Tecan Austria GmbH, Switzerland) at a wavelength of 620 nm.

As a standard of comparison, a drug of quinoline structure - nitroxoline was used.

References

1. Gao, H.; Ling, Y.; Xu, T.; Zhu, W.; Jing, H.; Sheng, W.; Li, Q. X.; Li, J. *J. Agric. Food Chem.* **2006**, *54*, 5284–5291.

2. Grychowska, K.; Olejarz-Maciej, A.; Blicharz, K.; Pietruś, W.; Karcz, T.; Kurczab, R.; Koczurkiewicz, P.; Doroz-Płonka, A.; Latacz, G.; Keeri, A. R.; Piska, K.; Satała, G.; Pęgiel, J.; Trybała, W.; Jastrzębska-Więsek, M.; Bojarski, A. J.; Lamaty, F.; Partyka, A.; Walczak, M.; Krawczyk, M.; Malikowska-Racia, N.; Popik, P.; Zajdel, P. *Eur. J. Med. Chem.* **2022**, *236*, 114329.

3. Cornaglia, G.; Hryniewicz, W.; Jarlier, V.; Kahlmeter, G.; Mittermayer, H.; Stratchounski, L.; Baquero, F. *Clin. Microbiol. Infect*. 2004, 10, 349–383.

4. CLSI. *Methods for Dilution Antimicrobial Susceptibility Tests for Bacteria That Grow Aerobically; Approved Standard—Tenth Edition*. CLSI document M07-A10. Wayne, PA: Clinical and Laboratory Standards Institute; **2015**.

5. ISO 20776-1:2006. *Clinical laboratory testing and in vitro diagnostic test systems - susceptibility test devices - Part 1: Reference method for testing the in vitro activity of antimicrobial agents against rapidly growing aerobic bacteria involved in infectious diseases*; **2006**.

6. Eucast Definitive Document. *Methods for the determination of susceptibility of bacteria to antimicrobial agents. Terminology. Clin. Microbiol. Infect*. **1998**, 4(5), 291-296.
